# Intermolecular N–N Coupling of a Dinitrosyl Iron Complex Induced by Hydrogen Bond Donors in the Secondary Coordination Sphere

**DOI:** 10.1021/jacs.4c12787

**Published:** 2025-02-19

**Authors:** Kayla M. Fugami, Gabriel S. Black, Tim Kowalczyk, Takele Seda, John D. Gilbertson

**Affiliations:** † Department of Chemistry, Western Washington University, Bellingham, Washington 98225, United States; ‡ Department of Physics, Western Washington University, Bellingham, Washington 98225, United States

## Abstract

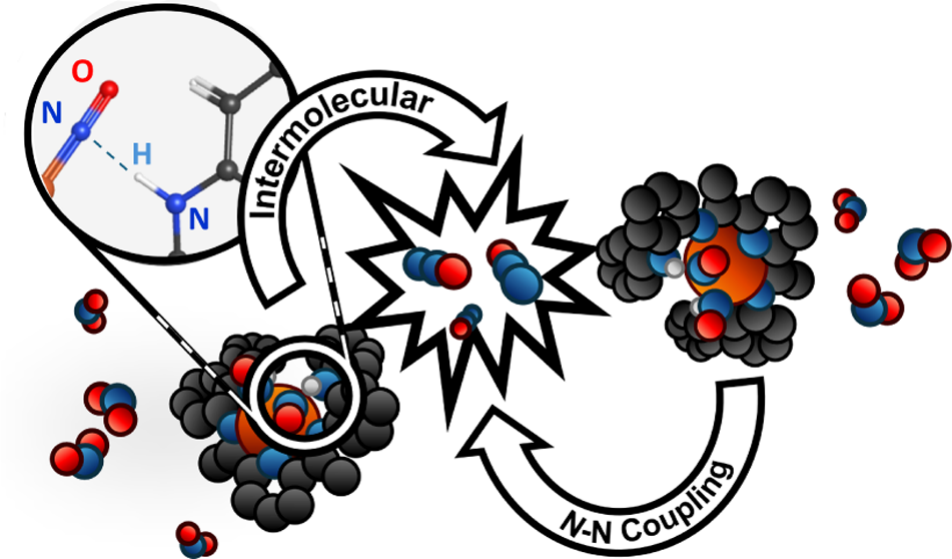

The intermolecular N–N coupling of NO in a dinitrosyl iron complex (DNIC) induced by hydrogen bond donors in the secondary coordination sphere to form N_2_O is reported. A family of complexes containing pendant anilines in the secondary coordination sphere were synthesized and characterized. Reduction of the {Fe(NO)_2_}^9^ complex [Fe(^PhNH^PDI)(NO)_2_][BPh_4_] (**3**) to the {Fe(NO)_2_}^10^ Fe(^PhNH^PDI)(NO)_2_ (**4**) results in intermolecular N–N coupling to form N_2_O. Similar reactions of the control {Fe(NO)_2_}^9^ complex [Fe(^PhNMe^PDI)(NO)_2_][BPh_4_] (**7**), which does not have H-bonding groups in the secondary coordination sphere, do not result in N_2_O formation. The hydrogen bonding capabilities of the complexes were explored spectroscopically and computationally.

## Introduction

Crop overfertilization has resulted in large anthropogenic sinks of nitrous oxide (N_2_O), which in the global nitrogen cycle is a byproduct of dissimilatory denitrification of nitrate (NO_3_^–^) to dinitrogen (N_2_).^1^ N_2_O is one of the leading causes of ozone depletion and a potent greenhouse gas (∼300 times larger warming potential than CO_2_).^2,3^ Elucidation of the mechanism of N_2_O formation is therefore extremely important and vital in our understanding of N–N bond forming reactions that result in N_2_O. In nature, the reductive coupling of nitric oxide (NO) to produce N_2_O in response to nitrosative stress in pathogenic bacteria and fungi is facilitated by nitric oxide reductases (NORs) and flavodiiron nitric oxide reductases (FNORs).^4−10^

**Figure 1:**
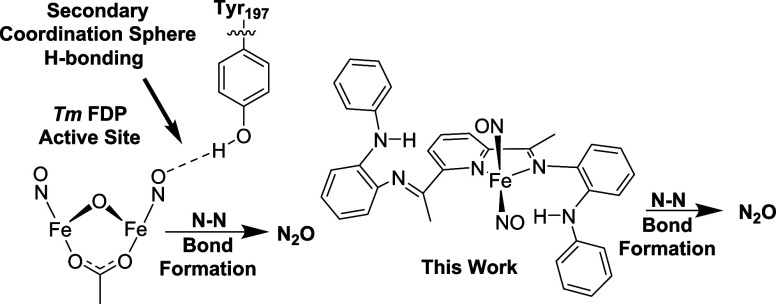
Active site of the *Tm* FDP highlighting the H-bonding interaction in the secondary coordination sphere (left) and the complexes described in this work with N–H groups in the secondary coordination sphere (right). Both systems display N–N coupling of NO to form N_2_O.

H-bonding tyrosine residues in the secondary coordination sphere of the flavodiiron protein (FDP) from *Thermotoga maritima* (*Tm*) are postulated to play a role in N_2_O formation from NO (Figure 1, left). *Tm* turns over NO to N_2_O at significant rates,^11^ and it has been shown that removal of the H-bonding residue Tyr_197_ in the secondary coordination sphere inhibits catalysis.^12^ In addition, computational studies indicate that the transient secondary sphere H-bonding lowers the activation barrier of ligand rotation required for catalysis.^200^

Dinitrosyl iron complexes (DNICs), on the other hand, are notoriously inert to N–N coupling, as indicated by the recent report of N–N coupling and release of N_2_O on the [(NacNac^Ar^)Fe(NO)_2_]^−^ (Ar = mesityl,2,6-diisopropylphenyl) DNICs.^13^ In that work, over the course of 5 days, a strongly Lewis acidic rare-earth, organometallic fragment is postulated to sterically force the nitrogen atoms into the correct conformation to allow NO intramolecular coupling (which is a spin-forbidden reaction)^14,15^ and subsequent N_2_O release. Given these results, and the fact that it has been hypothesized recently that H-bonding to NO ligands in DNICs may invoke reactivity,^16^ we decided to investigate the role that H-bond donors might play in the coupling of NO to form N_2_O. The work reported here (Figure 1, right) exploits pendant N–H groups in the secondary coordination sphere for the intermolecular N–N coupling of NO to form N_2_O.

## Results and Discussion

**Figure 2:**
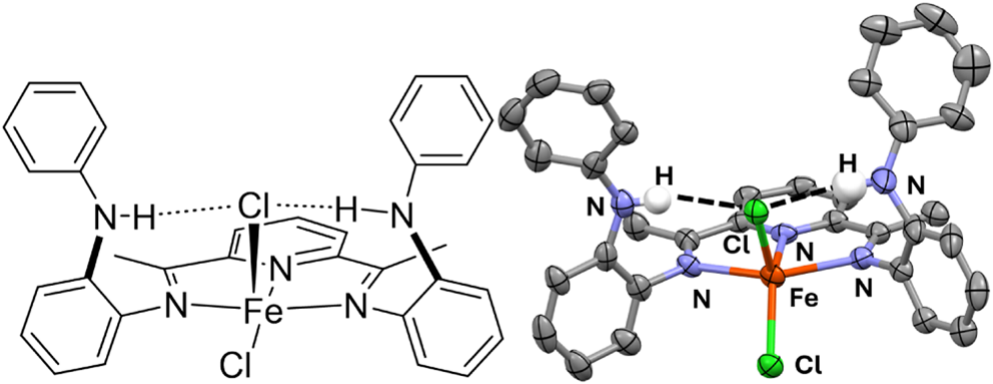
Chemdraw (left) and solid state structure (right) of Fe(^PhNH^PDI)Cl_2_ (**1**). Selected bond lengths (Å) and angles (deg): Fe(1)–N(1) 2.217(1), Fe(1)–N(2) 2.0084(9), Fe(1)–N(3) 2.240(1), Fe(1)–Cl(1) 2.299(4), Fe(1)–Cl(2) 2.316(5), N(1)–C(2) 1.286(1), N(3)–C(8) 1.290(1), C(2)–C(3) 1.487(2), C(7)–C(8) 1.486(2), and N(1)–Fe(1)–N(3) 142.88(4), N(2)–Fe(1)–Cl(1) 141.39(3).

The complex Fe(^PhNH^PDI)Cl_2_ (**1**, Figure 2) was synthesized from the Fe-mediated Schiff base condensation of 2,6-diacetylpyridine with *N*-phenyl-*o*-phenylenediamine in the presence of FeCl_2_ in EtOH in 81% yield (see Supporting Information for details). Single crystals of the blue product were obtained from the layering of diethyl ether onto a concentrated solution of CH_2_Cl_2_. An ORTEP view of **1** is shown in Figure 2 (right). The unit cell contains 5 independent molecules. The iron center(s) in the unit cell are five-coordinate with distorted square-pyramidal geometries (τ_5_ ranging from 0.15 to 0.27).^17^ The nitrogen atoms of the PDI ring, along with one chlorine atom, make up the basal plane, while the other chlorine atom occupies the apical position. The solid-state structure of **1** contains two intramolecular Metal Halogen Hydrogen Bonds (MHHBs) between the phenylaniline groups and the apical chlorine atom. The hydrogen atoms were located and refined. In a representative example, analysis of one independent molecule reveals the N–H(N)···Cl distances of 2.41(8) Å, and a N(4)···Cl(1) distance of 3.378(5) Å, consistent with an intramolecular H-bond.^18^ The NH groups are directed toward the Cl atoms [the N–H···Cl angles are both 163(6)°], which is also indicative of moderate intramolecular H-bonding.^19^ The ν_NH_ in the solid-state infrared spectrum is observed at 3273 cm^–1^. The room temperature zero-field Mössbauer parameters (Figure S3) [δ = 0.886(6) mm/s; Δ*E*_Q_ = 2.00(1) mm/s] support the assignment of an *S* = 2 iron center with a neutral ^PhNH^PDI ligand.^20,21^

**Figure 3:**
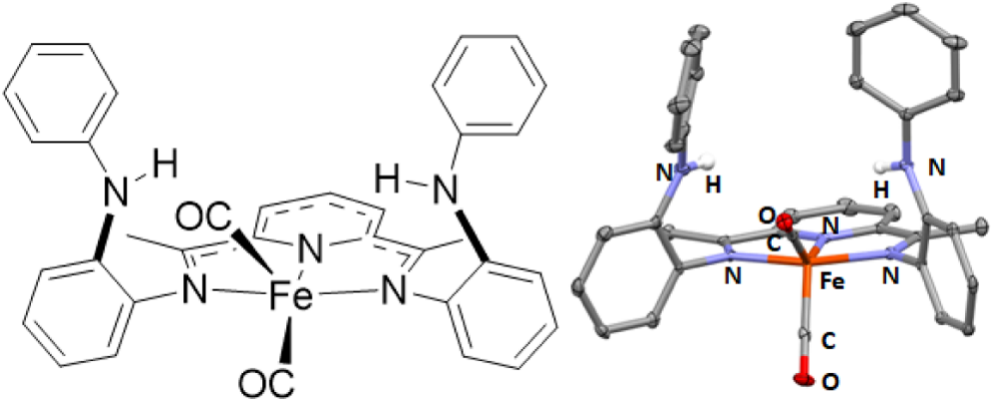
Chemdraw (left) and solid state structure (right) of one independent molecule of Fe(^PhNH^PDI)(CO)_2_ (**2**) in the unit cell. Selected bond lengths (Å) and angles (deg): Fe(1)–N(1) 1.960(9), Fe(1)–N(2) 1.847(8), Fe(1)–N(3) 1.943(7), Fe(1)–C(36) 1.78(1), Fe(1)–C(37) 1.77(1), N(1)–C(2) 1.34(1), N(3)–C(8) 1.33(1), C(2)–C(3) 1.42(1), C(7)–C(8) 1.42(1), and N(1)–Fe(1)–N(3) 156.3(3), N(2)–Fe(1)–C(36) 150.9(4).

**1** was reduced at low temperature with a solution of SmI_2_ in THF under a CO atmosphere to produce the deep green, direduced dicarbonyl Fe(^PhNH^PDI)(CO)_2_ (**2**), which can be isolated in 64% yield via slow evaporation in diethyl ether. An ORTEP view of **2** is shown in Figure 3 (right), which contains eight independent molecules per unit cell. The Fe center(s) in **2** remain five coordinate, distorted square pyramidal (τ_5_ = 0.15–0.06). The PDI ligand in **2** is in the direduced form, as indicated by the average C_imine_–N_imine_ bond lengths in **2**, which are elongated to 1.341 and 1.331 Å, and the C_imine_–C_ipso_ bond lengths are contracted to 1.419 and 1.420 Å, indicative of a direduced species. The room temperature zero-field Mössbauer parameters (Figure S7) [δ = −0.083(3); Δ*E*_Q_ = 1.337(5) mm/s], and the fact that the complex is diamagnetic in solid state and solution, support the assignment of an *S* = 0 iron center with a doubly reduced ^PhNH^PDI ligand.^22,23^

As shown in Figure 3, the N–H groups in **2** are oriented in the general direction of the basal CO ligand. However, the average N···C(O) distances of 3.245 and 3.205 Å, in addition to the average N–H···C bond angles of 109 and 118°, place these interactions in the weak category of mainly dispersion/electrostatic interactions. In addition, the ν_NH_ in the solid-state infrared spectrum of **2** is shifted to 3373 cm^–1^ and sharpened significantly (Figure S4).

Similar to our previously reported synthesis of {Fe(NO)_2_}^9^ DNICs on the PDI ligand core,^24,25^ the complex [Fe(^PhNH^PDI)(NO)_2_][X] (**3**) was synthesized by the reaction of **2** with two equivalents of NO_2_^–^ and four equivalents of H^+^ (where *X* = BPh_4_^–^ or PF_6_^–^). The infrared (IR) spectrum of **3** (Figure S9) shows two ν_NO_ at 1792 and 1718 cm^–1^, which shift to 1755 and 1687 cm^–1^ upon labeling with ^15^NO (utilizing ^15^N-labeled NO_2_^–^ in the synthesis of **3**). The Δν_NO_ (cm^–1^ between NO vibrations) of 74 cm^–1^ is in the range typical of a five-coordinate DNIC.^26^ The zero-field Mössbauer (Figure S11) parameters at room temperature are δ = 0.35(1); Δ*E*_Q_ = 0.89(2) mm/s: in the range of other reported {Fe(NO)_2_}^9^ DNICs.^27^ The ν_NH_ in the solid-state infrared spectrum of **3** is shifted to 3361 cm^–1^ and broadened significantly from that in **2**.

**Scheme 1:**
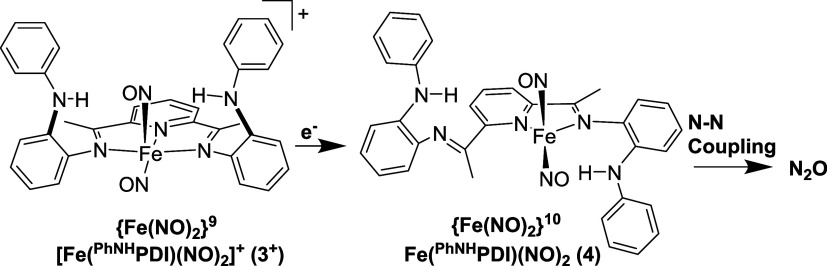
Reduction of the {Fe(NO)_2_}^9^ Complex [Fe(^PhNH^PDI)(NO)_2_]^+^ ([**3**]^+^; Counterion Omitted for Clarity) to the {Fe(NO)_2_}^10^ Fe(^PhNH^PDI)(NO)_2_ (**4**) with CoCp_2_ and Subsequent N–N Coupling to Form N_2_O

**Figure 4:**
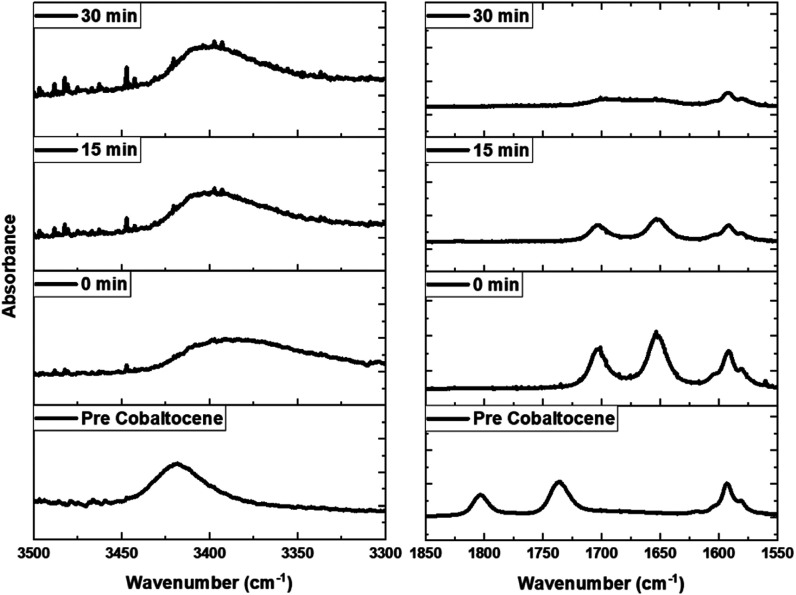
Solution cell FTIR spectra of the ν_NO_ region (right) and ν_NH_ region (left) of the reaction of [Fe(^PhNH^PDI)(NO)_2_]^+^ (**3**^**+**^) with CoCp_2_ in DCM showing the formation and subsequent disappearance of Fe(^PhNH^PDI)(NO)_2_ (**4**).

Reduction of the {Fe(NO)_2_}^9^**3** to the {Fe(NO)_2_}^10^ DNIC was explored (Scheme 1). Reaction of a DCM solution of **3** with one equivalent of CoCp_2_ results in a color change from brown to deep red. Solution phase Fourier transform infrared spectroscopy (FTIR) analysis (Figure 4) of the reaction mixture immediately upon addition of the cobaltocene displays a shift in the ν_NO_ from 1803 and 1736 cm^–1^ in the {Fe(NO)_2_}^9^**3** to 1700 and 1653 cm^–1^ (leaving the ν_C=N_ band at 1591 cm^–1^ due to the imine unchanged). This shift in the ν_NO_ is indicative of the formation of the four-coordinate {Fe(NO)_2_}^10^; Fe(^PhNH^PDI)(NO)_2_ (**4**). The Δν_NO_ of 47 cm^–1^ supports this assignment^26^ (Table 1).

**Table 1:** Selected Spectroscopic Data for Fe(PDI) DNICs

	**3**	**4**	**7**	**8**	**13**
aniline^a^	PhNH	PhNH	PhNMe	PhNMe	MeNH
ν_NO_ (cm^–1^)	1792, 1720	1700, 1653	1799, 1726	1686, 1636	1791, 1719
Δν_NO_ (cm^–1^)	72	47	73	50	72
*E*_1/2_ (V)^b^	–0.827, −1.53	-	–0.777, −1.58	-	–0.965, −1.70
δ (mm/s)	0.35(1)	-	0.45(3)	-	-
Δ*E*_Q_ (mm/s)	0.89(2)	-	1.05(6)	-	-

a Group located in the secondary coordination sphere.

b CH_3_CN vs Fc^+^/Fc, 0.1MTBAPF_6_, GC WE, Pt CE, Ag/Ag^+^ RE.

If the reaction is allowed to proceed for 2 h, the color changes from deep red to blue. Solution phase FTIR of the final reaction mixture does not display any ν_NO_ bands (Figure 4) and the imine band is still present. Inspection of the N–H region of the solution FTIR during the reaction reveals a shift from 3418 cm^–1^ for **3** to 3399 cm^–1^ immediately upon reduction. The N–H band broadens significantly without a loss in intensity, indicative of an N–H interaction in **4**. Over the course of the reaction (30 min) the N–H peaks shift to 3403 cm^–1^ with some slight sharpening. FTIR of the post reaction workup show the N–H peaks still visible in the product mixture at 3365 cm^–1^ and sharpened significantly (Figure S15).

Inspection of the reaction headspace shows the formation of N_2_O in ∼50% yield, indicative of N–N coupling. In order to determine if the N_2_O was formed intramolecularly or intermolecularly, a 1:1 mixture of ^14^N-labeled **3** and ^15^N-labeled **3** (synthesized from **2** and Na^15^NO_2_; see Supporting Information for details) was allowed to react with CoCp_2_ in DCM. As shown in Figure S42, the headspace of the reaction contains a mixture of ^14^N_2_O, ^15^N_2_O, ^14^N^15^NO, and ^15^N^14^NO isotopes indicative of intermolecular N–N coupling. In addition, the 1:1 mixture of ^14^N-**3** and ^15^N-**3** does not produce any mixed isotope **3**, indicating that the NO ligands in **3** are not labile.

FTIR analysis of the crude reaction mixture (Figure S13) from the N_2_O forming reaction reveals a υ_NO_ band at 1660 cm^–1^, which shifts to 1622 cm^–1^ upon ^15^N isotopic substitution, suggesting the presence of a mononitrosyl iron complex (MNIC). In addition, zero-field Mössbauer spectra of the same reaction Figure S14 show two iron sites in a roughly 50:50 occupancy ratio. Site A (52%) has the parameters δ = 0.38(1) mm/s; Δ*E*_Q_ = 0.895(8), consistent with the formation of an Fe_*x*_O_*y*_ species.^25,28^ The site B (48%) parameters δ = 0.040(4) mm/s; Δ*E*_Q_ = 0.946(5) are similar to the parameters reported for FePDI MNICs in the reduced form,^29^ consistent with the observed FePDI MNIC. These results align with the blue color of mixture, and the maximum yield of N_2_O detected at ∼50%. Unfortunately, the formed MNIC is fleeting, as attempts at purification and isolation were unsuccessful (FTIR analysis of the worked up reaction mixture shows no υ_NO_ bands; Figure S15).

In order to probe the role of the secondary sphere N–H groups in the N–N coupling reaction described above, the control complexes Fe(^PhNMe^PDI)Cl_2_, Fe(^PhNMe^PDI)(CO)_2_, and [Fe(^PhNMe^PDI)(NO)_2_][*X*], (**5**–**7**) were synthesized and characterized (See SI for details). In **5**–**7**, the N–H groups are replaced with N–CH_3_. These complexes are ideal surrogates as indicated by the ν_CO_ of **2** and **6** (1950 and 1894 cm^–1^ in **2**, and 1952 and 1882 cm^–1^ in **6**, Figure S19) and the ν_NO_ of **3** and **7** (1792 and 1718 cm^–1^ in **3**, and 1799 and 1726 cm^–1^ in **7**, Figure S20). In addition, the cyclic voltammograms of **3** and **7** in CH_3_CN exhibit nearly identical {Fe(NO)_2_}^9/10^ (−0.827 V in **3** and −0.777 V in **7**) and PDI^0/–^ couples vs Fc^+/0^ (−1.53 V in **3** and −1.58 V in **7**). Lastly, the zero-field Mössbauer (Figure S22) parameters at room temperature of δ = 0.45(3); Δ*E*_Q_ = 1.05(6) mm/s, are in line with those in **3**. These data indicate that replacing the N–H group for N–CH_3_ in the secondary coordination sphere does not alter the electronics of the complexes (Table 1).

**Scheme 2:**
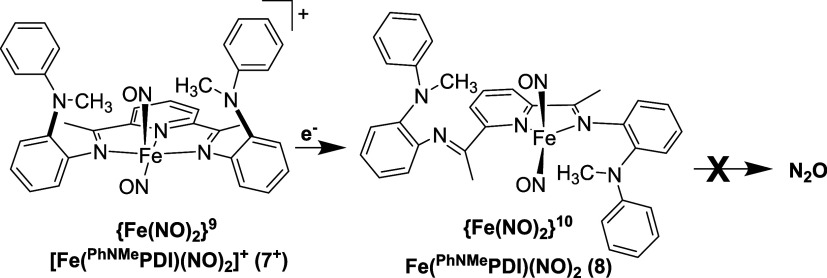
Reduction of the {Fe(NO)_2_}^9^ Complex [Fe(^PhNMe^PDI)(NO)_2_]^+^ ([**7**]^**+**^, Counterion Omitted for Clarity) to the {Fe(NO)_2_}^10^ Fe(^PhNMe^PDI)(NO)_2_ (**8**) with CoCp_2_ No subsequent N–N coupling to N_2_O is observed.

Reduction of the {Fe(NO)_2_}^9^**7** to the {Fe(NO)_2_}^10^ DNIC (and subsequent N–N coupling) was explored (Scheme 2). Similar to **3**, reaction of a DCM solution of **7** with one equivalent of CoCp_2_ results in the same color change from brown to deep red. Solution phase FTIR analysis of the reaction mixture immediately upon addition of the cobaltocene also displays a shift in the ν_NO_ from 1802 and 1734 cm^–1^ in the {Fe(NO)_2_}^9^**7** to 1700 and 1649 cm^–1^ (Figure S38). This shift is indicative of the formation of the four-coordinate {Fe(NO)_2_}^10^; Fe(^PhNMe^PDI)(NO)_2_ (**8**). The Δν_NO_ of 51 cm^–1^ supports this assignment. Contrary to the observations in **3**, allowing the reaction to proceed for 2 h, no color change to blue occurs and only trace N_2_O is observed in the headspace. Solution phase FTIR of the final reaction mixture still displays the ν_NO_ bands due to the existence of **8**. These data indicate that N–N coupling does not occur with **8**. In addition, control reactions of **8** and exogenous diphenylamine (See Supporting Information for details) do not decompose **8** and do not produce any N_2_O. These experiments demonstrate that the N–H group in the secondary coordination sphere is crucial in the N–N coupling reaction.

Additionally, we explored a complex capable of forming H-bonds in the secondary coordination sphere to see if it could induce N–N bond formation. The {Fe(NO)_2_}^10^ DNIC Fe(didpa)(NO)_2_ (**9**), contains the pendant base diisopropylamine in the secondary coordination sphere.^25^ We have previously shown that the didpa ligand scaffold is capable of being protonated and forming H-bonds in the secondary coordination sphere.^30^ As shown in eq 1, reaction of **9** with one equivalent of [HNEt_3_][BPh_4_] in DCM does not result in the formation of N_2_O, rather oxidation of **9** to the {Fe(NO)_2_}^9^; [Fe(didpa)(NO)_2_]^+^ (**10**^**+**^) with the concomitant formation of H_2_ results instead. This experiment highlights the importance of the p*K*_a_ of the N–H group in the secondary coordination sphere. The R_3_N–H^+^ group in diisopropylamine (p*K*_a_ = 18.8; CH_3_CN)^31^ is too acidic for the reduced {Fe(NO)_2_}^10^, whereas *N*-phenylaniline (p*K*_a_ = 5.97; CH_3_CN) lies in the range that does not induce oxidation of the {Fe(NO)_2_}^10^, allowing subsequent N–N bond formation to occur.
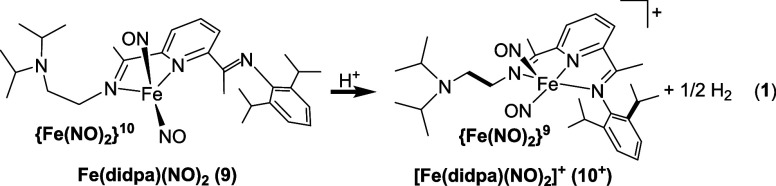
1

**Figure 5:**
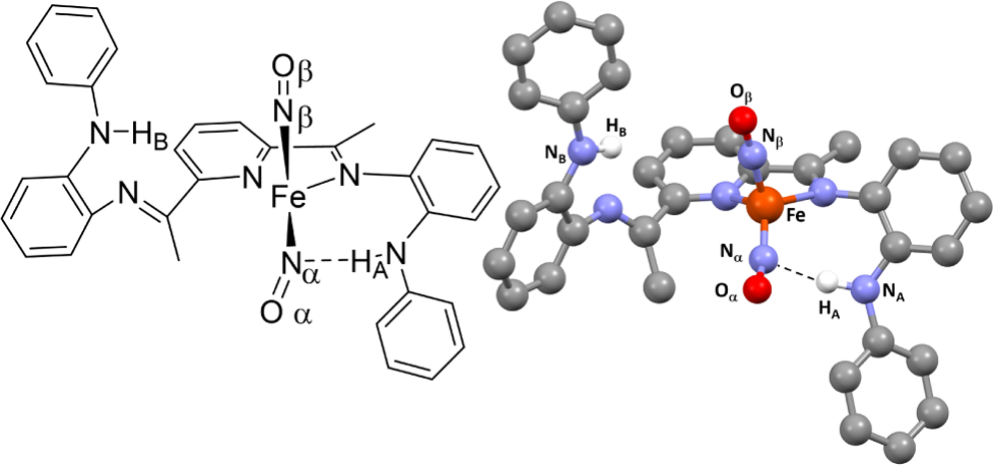
Chemdraw (left) and computed structure (right) of 4* showing the H_A_–N_α_(O) hydrogen bonding interaction.

Given the fleeting nature of **4**, which induces *inter*molecular N–N coupling to form N_2_O, we turned to computations to glean insight into the nature of any hydrogen bonding that may be favored in the complex. As shown in Figure 5, geometry optimization of **4** at the PBE-D3/def2-TZVP level^32^ resulted in conformation 4* in which one of the N–H groups is directed toward the Fe(NO)_2_ unit. The calculations assume {Fe(NO)_2_}^10^ character with singlet spin symmetry. In the computed structure, the two Fe–N–O bond angles differ from each other by ∼15°, where the Fe–N_α_–O_α_ is 164°, and the Fe–N_β_–O_β_ is much more linear, at 178° (Table S2). (The α represents the group in close proximity to the H-bond donor.) The N_A_–N_α_(O) distance of 3.247 Å and the H_A_–N_α_(O) distance of 2.367 Å would indicate a weak interaction, while the N–H–N bond angle of 143.8° would be categorized as moderate.

The bond dissociation energy for the H_A_–N_α_(O) interaction in 4* was examined through relaxed coordinate scans along the postulated hydrogen-bonded N–H distance. Starting from the minimum-energy configuration of 4*, the potential energy was assessed in regular increments to beyond 5 Å. All other degrees of freedom were unconstrained during these scans. As shown in Figure S60, the PBE-D3/TZVP scan shows a clear plateau between 3–4 Å associated with weakening of the H-bond interaction. The well depth of 4.4 kJ/mol relative to the plateau suggests moderate-to-weak H-bonding interaction.

Typically {Fe(NO)_2_}^10^ DNICs are stable given their closed shell, 10 d/π electron configuration in the Enemark-Feltham notation.^33^ N–N coupling in these systems is well documented to be spin-forbidden, given the antiferromagnetic coupling between iron center and the nitrosyl ligands.^14,15^ In our previous work^25^ demonstrating intermolecular N–N coupling of dinuclear DNICs, it was clear that N–N bond formation was precluded by the formation of an electron deficient {Fe(NO)_2_}^10^ center. In the case of **4**, the ν_NO_ do not indicate an electron deficient {Fe(NO)_2_}^10^, as the bands for **4** and **8** are nearly identical. Given the shift and broadening of the N–H bands in **4**, the implication is that a hydrogen bonding interaction is likely responsible for inducing N–N bond formation. While we cannot completely rule out the role of the N–H group as an H atom donor (which could potentially act as an HNO surrogate),^34^ the control reaction with an excess of Ph_2_NH and **8** does not show any reaction (Figure S43) or N_2_O formation. The N–H BDFE in Ph_2_NH is tabulated at 79.0 kcal/mol in the gas phase and 80.0 kcal/mol in solution (C_6_H_6_).^35^ In addition, reaction of **8** with 9,10-dihydroanthrocene (DHA) does not react to produce N_2_O or H_2_ either (Figure S45). The BDFE of the C–H bond in DHA is calculated to be 72.9 kcal/mol (DMSO). Lastly, the deuterated analog [Fe(^PhND^PDI)(NO)_2_][BPh_4_] (**3**^**ND**^), where the N–H group is replaced by N-D, was synthesized by stirring [Fe(^PhNH^PDI)(NO)_2_][BPh_4_] (**3**) in a mixture of CD_3_CN and D_2_O for 2 days. As indicated by the FTIR spectrum (Figure S49), the N–H stretch at 3361 cm^–1^ is replaced by a band at 2497 cm^–1^, corresponding to the N-D stretch (2555 cm^–1^ predicted). As shown in Figure S50, in the N_2_O forming reaction, [Fe(^PhND^PDI)(NO)_2_][BPh_4_] (**3**^**ND**^) proceeds at the same rate as the proteo- analog [Fe(^PhNH^PDI)(NO)_2_][BPh_4_] (**3**), further indicating that proton transfer is not likely occurring.

**Figure 6:**
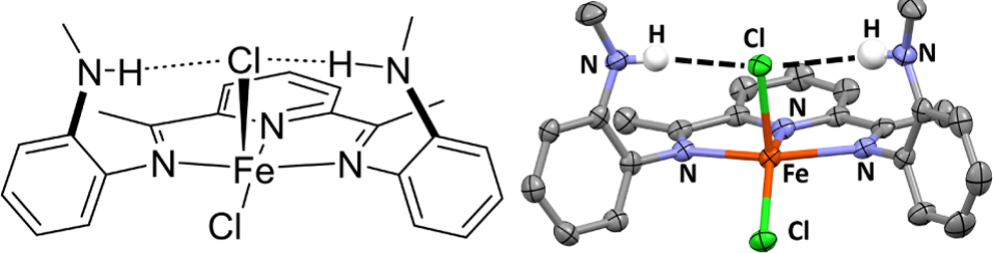
Chemdraw (left) and solid state structure (right) of one independent molecule in the unit cell of Fe(^MeNH^PDI)Cl_2_ (**11**). Selected bond lengths (Å) and angles (deg): Fe(1)–N(1) 2.203(2), Fe(1)–N(2) 2.086(2), Fe(1)–N(3) 2.194(2), Fe(1)–Cl(1) 2.3537(7), Fe(1)–Cl(2) 2.2598(8), N(1)–C(2) 1.290(3), N(3)–C(8) 1.287(3), C(2)–C(3) 1.487(4), C(7)–C(8) 1.490(3), and N(1)–Fe(1)–N(3) 147.94(7), N(2)–Fe(1)–Cl(2) 140.76(6).

A final series of experiments also highlight the role of the pendant N–H groups. The -R group of the pendant aniline were changed from phenyl to methyl. The complexes Fe(^MeNH^PDI)Cl_2_, Fe(^MeNH^PDI)(CO)_2_, and [Fe(^MeNH^PDI)(NO)_2_][X], (**11**–**13**) were synthesized and characterized (See Supporting Information for details). The ^MeNH^PDI ligand is also capable of weak H-bonding to the Fe–Cl unit of **11**, as indicated by the two intramolecular MHHBs between the methylaniline groups and the apical chlorine atom (Figure 6). The hydrogen atoms were located and refined to yield N–H(N)···Cl distances of 2.49(3) and 2.418(3) Å, and N···Cl distances of 3.264(2) and 3.352(2) Å, consistent with a weak intramolecular H-bond.^19^ The NH groups are directed toward the Cl atoms [the N–H···Cl angles are 158(3) and 162(2)°], which is indicative of moderate intramolecular H-bonding. The ν_NH_ in the solid-state infrared spectrum is observed at 3315 cm^–1^, sharper and blue-shifted compared to **1**. This is consistent with the p*K*a of *N*-methylaniline (10.97; CH_3_CN) and BDFE (86.4; gas). Both indicate that the pendant group should be a somewhat poorer H-bond donor than the phenyl group.

The electronics of the metal centers in complexes (**11**–**13**) are nearly identical to the ^PhNH^PDI complexes (**1**–**3**) as indicated by the ν_CO_ of 1950 and 1890 cm^–1^ in Fe(^MeNH^PDI)(CO)_2_ (**12**) and the ν_NO_ of 1791 and 1719 cm^–1^ in the five coordinate {Fe(NO)_2_}^9^ [Fe(^MeNH^PDI)(NO)_2_][X] (**13**) (Table 1). Reduction of **13** to the four coordinate {Fe(NO)_2_}^10^ Fe(^MeNH^PDI)(NO)_2_ (**14**) also results in the formation of N_2_O in similar yields (50%). The time scale on which **14** undergoes N–N coupling to form N_2_O is near identical to **4** (Figure S48). This observation is counter to the predicted result if the N–H group were acting as an H atom donor. The disparate values of BDFE and p*K*a between Ph_2_NH and MePhNH would likely produce a difference in the rates of N_2_O formation. As in 4*, geometry optimizations of 14* at the PBE-D3/def2-TZVP level resulted in a conformation in which one of the N–H groups is directed toward the Fe(NO)_2_ unit. The N_A_–N_α_(O) distance of 3.236 Å and the H_A_–N_α_(O) distance of 2.432 Å, coupled with N–H–N bond angle of 135.2° (Table S2) would be categorized as a weaker interaction than in 4*. The relaxed coordinate scan analysis (Figure S60) at the PBE-D3/TZVP level shows a well depth of 5.2 kJ/mol relative to the plateau. The energy difference is smaller than the statistical accuracy of the underlying dispersion-corrected DFT energies, suggesting similar H-bonding interactions in both 4* and 14*.

Given the dearth of reports of N–N coupling in DNICs,^36^ we looked for precedence in N–N coupling in mononitrosyl iron complexes (MNICs) induced by H-bonds. Previous work has invoked hydrogen bonding as a facilitator to N–N coupling in MNICs.^37,38^ For example, in the case of the {Co(NO)}^9^ [Co(CTPPMe)(NO)] (where CTPPMe is an *N*-confused porphyrin), MeOH was shown to form an H-bonded species which resulted in a hyponitrite [N_2_O_2_]-bridged dimer and subsequently N_2_O. In addition, the chelating ligand bearing a secondary sphere hydrogen bonding functionality, (PV-tmpa, where PV = pivalamido) was shown to facilitate NO coupling by the [Cu(PV-tmpa)]^+^ complex.^39^ H-bond stabilization of the hyponitrite bridged intermediate by the pivalamido group was followed by proton transfer to form N_2_O and the deprotonated complex. H-bonding interactions have also been shown to induce back-donation into the 2π* orbital of NO to facilitate NO cleavage in heterogeneous surface catalysts.^40^ Lastly, previous work on the Fe(OEP)(NO)– complex (where OEP = octaethylporphyrin) has shown that the protonated Fe(OEP)(HNO) is in equilibrium with the H-bonded complex Fe(OEP)(NO)–HOPh, demonstrating the utility of H-bonding to the N atom of the NO ligand.^41^ In all of these cases, polarization of the N–O bond is a direct consequence of H-bonding.

Among the possible mechanisms for N–N bond formation; two types may be possible in the current work. The first is the NOR type reaction^42^ (2NO + 2e^–^ + 2H^+^ → N_2_O + H_2_O) and the second is NO disproportionation (3NO → N_2_O + NO_2_). Headspace analysis of the N_2_O reaction mixture does not show any bands indicative of NO_2_ or free NO. A CoTPP trapping experiment (Figure S55) was also negative for free NO. These results would indicate that NO disproportionation is unlikely. It should be noted here, however, that our previous results demonstrating intermolecular N–N coupling in dinuclear DNICs showed that any NO generated in situ will also induce N_2_O formation (through a presumed trinitrosyl intermediate), so it cannot be ruled out completely.^25^

**Scheme 3:**
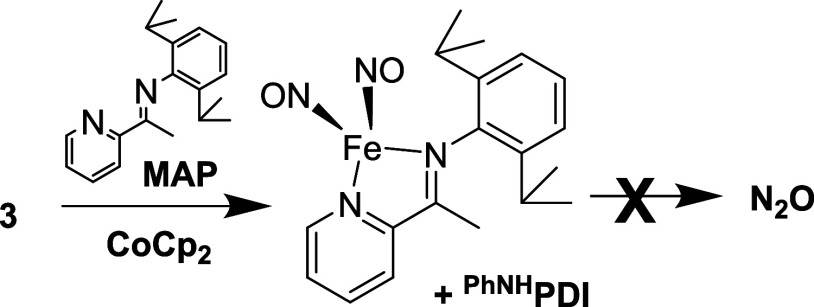
Reduction of [Fe(^PhNH^PDI)(NO)_2_][BPh_4_] (**3**) in the Presence of Excess MAP Ligand and Subsequent Fe(NO)_2_ Unit Scrubbing, which Inhibits N_2_O Formation

**Scheme 4:**
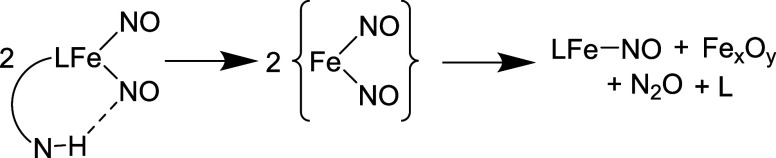
Proposed Mechanism of H-Bond Induced Fe(NO)_2_ Lability which Results in N–N Bond Formation *L* = ^PhNH^PDI is abbreviated for clarity.

In terms of the mechanism of N–N bond formation, previous work has shown that in some cases, the {Fe(NO)_2_}^10^ unit can be labile.^43^ In order to probe the role of {Fe(NO)_2_}^10^ unit lability in N_2_O formation, the reactions were carried out in the presence of the chelating monoiminopyridine (MAP) ligand shown in Scheme 3. We have previously shown that the MAP ligand can form stable {Fe(NO)_2_}^9^ and {Fe(NO)_2_}^10^ DNICs,^25^ and that those DNICs do not couple to form N_2_O. We first examined if MAP could remove the {Fe(NO)_2_}^9^ unit from [Fe(^PhNH^PDI)(NO)_2_][BPh_4_] (**3**) by reacting four equivalents of MAP with [Fe(^PhNH^PDI)(NO)_2_][BPh_4_] (**3**) and monitoring the ^1^H NMR and FTIR spectra. Figure S53 shows that MAP does not remove the {Fe(NO)_2_}^9^ unit (no change in the υ_NO_). Similar results are observed in the reaction of MAP with the {Fe(NO)_2_}^10^ unit in Fe(^PhNMe^PDI)(NO)_2_ (**8**) which also indicate that the {Fe(NO)_2_}^10^ unit is not labile (Figure S51). However, when [Fe(^PhNH^PDI)(NO)_2_][BPh_4_] (**3**) is reduced with CoCp_2_ in the presence of four equivalents of MAP, no N–N coupling is observed (Figure S52). In addition, the FTIR spectrum shows the formation of the {Fe(NO)_2_}^10^ complex, Fe(MAP)(NO)_2_ (Figure S53). The results indicate that the Fe(NO)_2_ unit in the formed Fe(^PhNH^PDI)(NO)_2_ (**4**) is labile, while the Fe(NO)_2_ unit in Fe(^PhNMe^PDI)(NO)_2_ (**8**) is not. Clearly, Fe(NO)_2_ unit lability would explain the observation of intermolecular N–N coupling. While we cannot fully rule out the role of sterics in N–N bond formation (see below), the N–H group is likely responsible for Fe(NO)_2_ unit polarization that subsequently induces Fe(NO)_2_ unit lability, as proposed in Scheme 4.

Finally, given the observations of the N–N bond forming reaction described above is reliant on the secondary coordination sphere, we wanted to probe the role of the pendant group sterics in N_2_O formation. We synthesized the ^BA^PDI ligand shown in eq 2, which is a sterically unencumbered ligand that possesses a C–H bond in the place of the PhN-H group. As shown in Figures S58 and S59, the in situ generated Fe(^BA^PDI)(NO)_2_ (**15**) is a stable {Fe(NO)_2_}^10^ complex that does not undergo N–N coupling to form N_2_O. The ν_NO_ of 1703 and 1657 cm^–1^ and Δν_NO_ of 46 cm^–1^ confirm the four coordinate {Fe(NO)_2_}^10^ assignment. Given that Fe(^BA^PDI)(NO)_2_ (**15**) is stable in both solid state and solution, these results likely indicate that sterics are not playing a significant role in N_2_O formation.
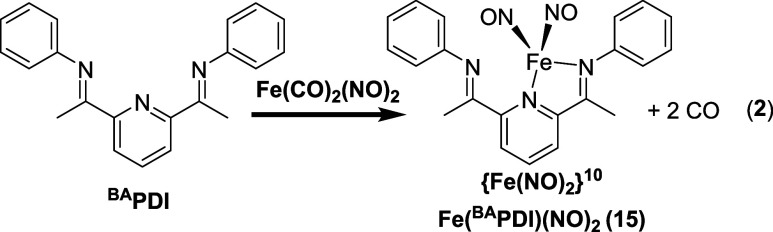
2

## Conclusions

In conclusion, we have presented a series of DNICs with N–H groups located in the secondary coordination sphere, which can be exploited for the intermolecular N–N coupling of NO to form N_2_O. Reduction of the {Fe(NO)_2_}^9^ [Fe(^PhNH^PDI)(NO)_2_][X] (**3**) to the {Fe(NO)_2_}^10^ Fe(^PhNH^PDI)(NO)_2_ (**4**) results in the intermolecular coupling of NO. The pendant aniline is vital for reactivity, as replacement of the N–H group with N-Me inhibits N–N bond formation. Reduction of the {Fe(NO)_2_}^9^ [Fe(^PhNMe^PDI)(NO)_2_][X] (**7**) to the {Fe(NO)_2_}^10^ Fe(^PhNMe^PDI)(NO)_2_ (**8**) does not form N_2_O. Close proximity of the N–H group to the DNIC unit is also a requisite; exogenous *N*-phenylaniline does not induce N–N bond formation in the {Fe(NO)_2_}^10^ Fe(^PhNMe^PDI)(NO)_2_ (**8**). The addition of weak C–H-bond donors does not induce N–N bond formation either, making H atom transfer to form HNO, and subsequently N_2_O unlikely. In addition, no kinetic isotope effect is observed when [Fe(^PhND^PDI)(NO)_2_][BPh_4_] (**3**^**ND**^) is used in place of (**3**).

Computational analysis of the N–H···N(O) interactions indicate that a weak/moderate interaction is possible. This interaction is likely responsible for Fe(NO)_2_ unit polarization that subsequently induces Fe(NO)_2_ unit lability in the *inter*molecular N–N coupling reaction. Sterics are not likely playing a role as indicated by the formation of the stable {Fe(NO)_2_}^10^ Fe(^BA^PDI)(NO)_2_ (**15**). This work demonstrates that secondary interactions are important in N–N coupling. In addition, this is a novel reaction demonstrating the potential role of DNICs in the coupling of NO to form N_2_O.

## Supporting Information Available

Experimental section; synthetic procedures; characterization data; N_2_O formation studies (PDF)
