# Supplementary material for: Intermolecular N–N Coupling of a Dinitrosyl Iron Complex Induced by Hydrogen Bond Donors in the Secondary Coordination Sphere
Source: J Am Chem Soc. 2025 Feb 19;147(9):7274–81. doi: 10.1021/jacs.4c12787 (PMC11887047; doi:10.1021/jacs.4c12787)
Supplement: Supplementary file 1 — ja4c12787_si_001.pdf [file ja4c12787_si_001.pdf]

Supporting Information for Intermolecular N-N Coupling of a Dinitrosyl Iron Complex Induced by Hydrogen Bond Donors in the Secondary Coordination Sphere

Kayla M. Fugami, † Gabriel S. Black,† Tim Kowalczyk,† Takele Seda,‡ and John D. Gilbertson*†

†Department of Chemistry, Western Washington University, Bellingham, Washington 98225, United States
‡Department of Physics, Western Washington University, Bellingham, Washington 98225, United States

Table of Contents

Supplemental Experimental S3- S7

Fe(^{PhNH}PDI)Cl$_2$ (1)
- FT-IR S8
- ^{1}H NMR S8
- Zero-field Mössbauer S9

Fe(^{PhNH}PDI)(CO)$_2$ (2)
- FT-IR S9
- ^{1}H NMR S10
- ^{13}C NMR S10
- Zero-field Mössbauer S11
- Cyclic voltammogram S11

[Fe(^{PhNH}PDI)(NO)$_2$][BPh$_4$] (3)
- FT-IR S12
- ^{1}H NMR S12
- Zero-field Mössbauer S13
- Cyclic voltammogram S13

Crude Reaction Mixture from the N-N Coupling Reaction of [Fe(^{PhNH}PDI)(NO)$_2$] (4)
- FT-IR S14
- Zero-field Mössbauer S14

Post Filtration Blue Product from the N-N Coupling Reaction of [Fe(^{PhNH}PDI)(NO)$_2$] (4)
- FT-IR S15

Fe(^{PhNMe}PDI)Cl$_2$ (5)
- FT-IR S15
- ^{1}H NMR S16
- Chemdraw and XRD Crystal Structure S16

Fe(^{PhNMe}PDI)(CO)$_2$ (6)
- FT-IR S17

[Fe(^{PhNMe}PDI)(NO)$_2$][BPh$_4$] (7)
- FT-IR S17
- ^{1}H NMR S18
- Zero-field Mossbauer S18

Cyclic voltammogram S19
Fe(^{PhNMe}PDI)(NO)$_2$ (8)
FT-IR S19
^{1}H NMR S20
^{13}C NMR S20
[Fe(^{PhNH}PDI)(^{15}NO)$_2$][BPh$_4$] (3-^{15}N)
FT-IR S21
Fe(^{MeNH}PDI)Cl$_2$ (11)
FT-IR S21
^{1}H NMR S22
Fe(^{MeNH}PDI)(CO)$_2$ (12)
FT-IR S22
^{1}H NMR S23
^{13}C NMR S23
Cyclic voltammogram S24
[Fe(^{MeNH}PDI)(NO)$_2$][BPh$_4$] (13)
FT-IR S24
^{1}H NMR S25
Cyclic voltammogram S25
Blue Product from the N-N Coupling Reaction of [Fe(^{MeNH}PDI)(NO)$_2$][BPh$_4$] (13)
FT-IR S26
Reduction of [Fe(^{PhNMe}PDI)(NO)$_2$][BPh$_4$] (7) with Cobaltocene
Solution Phase FT-IR S26
Overlayed FT-IR spectra of Crude Reaction Mixture from N-N Coupling Reaction of [Fe(^{PhNH}PDI)(NO)$_2$][BPh$_4$] (3) and [Fe(^{PhNH}PDI)(^{15}NO)$_2$][BPh$_4$] (3-^{15}N).
FT-IR S27
Overlayed FT-IR Spectra of [Fe(^{PhNH}PDI)(NO)$_2$][BPh$_4$] (3) and [Fe(^{PhNH}PDI)(^{15}NO)$_2$][BPh$_4$] (3-^{15}N) Reduction with Cobaltocene with Filtration Step
FT-IR S27
Gas Phase FT-IR S28
Gas Phase FT-IR Spectrum of [Fe(^{PhNH}PDI)(NO)$_2$][BPh$_4$] (3) and [Fe(^{PhNH}PDI)(^{15}NO)$_2$][BPh$_4$] (3-^{15}N) Mixed Isotope Reduction with Cobaltocene
Experimental S29
Gas Phase FT-IR S29
Reaction of Fe(^{PhNMe}PDI)(NO)$_2$ (8) with Excess Ph$_2$NH
Experimental S30
Gas Phase FT-IR S30
^{1}H NMR S31
Scheme 1 S31
Reaction of Fe(^{PhNMe}PDI)(NO)$_2$ (8) with Excess 9,10-Dihydroanthracene (DHA)
Experimental S32
^{1}H NMR S32

Scheme 2 S32
Protonation of Fe(didpa)$(NO)_2$ (9) with $[HNEt_3][BPh_4]$
Experimental S33
Solution Phase FT-IR & Gas Phase FT-IR S33
Scheme 3 S33
Reduction of $[Fe(^{MeNH}PDI)(NO)_2][BPh_4]$ (13) with Cobaltocene
Gas Phase FT-IR S34
Solution Phase FT-IR S34
Reduction of $[Fe(^{PhND}PDI)(NO)_2][BPh_4]$ (3ND) with Cobaltocene
Experimental S35
FT-IR S35
Solution Phase FT-IR S36
Reduction of $[Fe(^{PhNH}PDI)(NO)_2][BPh_4]$ (3) and MAP with Cobaltocene
Experimental S36-S37
Scheme 4 S37
^{1}H NMR S37
Table S1 S38
Gas Phase FT-IR S38
Solution Phase FT-IR S39
Reduction of $[Fe(^{PhNH}PDI)(NO)_2][BPh_4]$ (3) with CoTPP & Cobaltocene
Experimental S39
Scheme 5 S40
FT-IR S40
UV-Vis S41
Synthesis of $Fe(^{BA}PDI)(NO)_2$ (15)
Experimental S41
Scheme 6 S42
Scheme 7 S42
FT-IR S42
^{1}H NMR S43
Solution Phase FT-IR S43
FT-IR S44
Crystallography Experimental S45
Crystallographic Data for $Fe(^{PhNH}PDI)Cl_2$ (**1**), $Fe(^{PhNH}PDI)(CO)_2$ (**2**), $Fe(^{PhNMe}PDI)Cl_2$ (**5**), and $Fe(^{MeNH}PDI)Cl_2$ (**9**) S46
Computational Experimental S46
Table S2 S47
Relaxed Coordinate Scans S47
Optimized geometries of $Fe(^{PhNH}PDI)(NO)_2$ (**4***), $Fe(^{PhNMe}PDI)(NO)_2$ (**8***), and $Fe(^{MeNH}PDI)NO_2$ (**14***). S48-S54
References S55

Materials and General Methods:

Unless otherwise noted, all reagents were purchased from commercial sources and used without further purification. The molarity of SmI_2 in THF was confirmed through analysis of the λ_{max} = 618 nm and ε = 877 L mol^{-1} cm^{-1} via UV-vis spectroscopy.[1] Dry and air-free solvents were obtained with a PureSolv solvent still (Vacuum Atmospheres Inc.). Gases were purchased from AirGas Inc. All air-sensitive reagents were handled in an N_2 filled inert atmosphere glovebox (operated at ambient temperature, typically 20-25 °C) or on a Schlenk line following standard air-free techniques. A polycarbonate blast shield was utilized for all reactions involving pressurized vessels. Fourier transform infrared spectra (FT-IR) were collected on a Thermo iS10 FT-IR spectrometer. Attenuated total reflectance (ATR) FT-IR spectra were collected on a single-bounce diamond ATR accessory. ATR FT-IR spectra were collected at four cm^{-1} resolution and 16 scans. Solution phase FT-IR spectra were collected in a liquid FT-IR cell equipped with CaF_2 windows. Gas phase FT-IR spectra were collected with a Pike Technologies short-path-length (100 mm) gas transmission cell fitted with CaF_2 windows. Both solution and gas phase FT-IR spectra were taken at 0.5 cm^{-1} resolution and 32 scans. All nuclear magnetic resonance (NMR) spectra were collected on a Bruker Advance III 500 MHz instrument operating at 499.75 MHz for ^{1}H spectra, and 125.66 MHz for ^{13}C{^{1}H} spectra. Spectra were referenced to CD_2Cl_2 for all ^{1}H (δ: 5.32 ppm) and ^{13}C{^{1}H} (δ: 53.42 ppm) experiments. Cyclic voltammetry (CV) spectra were collected with a Pine WaveNow potentiostat, glassy carbon working electrode, platinum wire counter electrode, and a nonaqueous silver nitrate reference electrode. The electrolyte utilized for all CV experiments was 0.1 M tetrabutylammonium hexafluorophosphate in acetonitrile. After initial CV collection, ~two mg of ferrocenium was added to the vial containing the complex in question to correct silver nitrate reference to ferrocenium. A constant-acceleration spectrometer (WissEl Gmbh, Germany) in a horizontal transmission mode using a 50 mCi^{57} Co source was utilized for Mössbauer spectra collection. All Mössbauer spectra were collected at room temperature with approximately 150 mg of sample loaded into an acrylic sample holder with minimal Paratone-N oil to prevent oxidation. Lorentzian line shape with the *NORMOS* (WissEl Gmbh) least-squares fitting program was used to fit spectra and isomer shifts were normalized to metallic iron. Fe(didpa)$(NO)_2$ (**9**) and the chelating monoiminopyridine (MAP) ligand were synthesized according to literature procedure.[2]

N_2O Quantification with Piloty's Acid. Piloty's acid (0.0186 g, 0.107 mmol) and a stir bar were added into a 20 mL scintillation vial and sealed with a 33 Suba-seal septum. The vial was schlenked by pulling vacuum for 30 seconds and refilled with N_2 gas for 10 seconds, this was repeated three times. At this point 10 mL of NaOH base with a pH of 13 was syringed into the vial. To ensure the completion of the reaction the vial was stirred overnight. Keeping the schlenking method consistent, the gas FT-IR cell was schlenked three times and used as the background. Then the vial's headspace was collected. Moles of N_2O formed by the complexes in question was quantified by relating the moles of N_2O formed and its peak integration.

Experimental:

Synthesis of Fe(^{PhNH}PDI)Cl_2 (1). In a 200 mL Schlenk flask, 2,6-diacetylpyridine (0.400 g, 2.45 mmol) and $FeCl_2$ (0.311 g, 2.45 mmol) were dissolved in ~20 mL of anhydrous EtOH and capped with a 45 Suba-seal septum. The flask was stirred at 50 °C for 30 minutes. In a 20 mL scintillation vial, *N*-Phenyl-*o*-phenylenediamine (0.903 g, 4.90 mmol) was dissolved in 10 mL of EtOH and capped with a size 33 Suba-seal septum. This solution was then slowly syringed into the flask and the solution was then heated to 80 °C and allowed to stir overnight. The solvent was removed *in vacuo,* resulting in a dark brown solid. The resulting solid was redissolved in ~20 mL of CH_2Cl_2 and filtered through celite. The solution was layered with ether for crystallization, resulting in dark brown crystals Fe(^{PhNH}PDI)Cl_2 (**1**). For X-ray quality crystals, the filtered CH_2Cl_2 solution was layered with pentane (1.24 g, 81.3% yield). FT-IR: ν_{NH} = 3273 cm^{-1}. $\nu_{C=N}$ = 1587 cm^{-1}, ^{1}H NMR (CD_2Cl_2) δ: 87.37, 17.74, 15.66, 9.86, 8.18, 7.73, 7.23, 3.04, -6.30, -6.84, -9.72, -11.75 ppm. Mössbauer: δ = 0.886(6) mm/s; ΔE_Q = 2.00(1) mm/s. Anal. Calcd for $C_{33}H_{29}Cl_2FeN_5$: C, 63.69; H, 4.70; N, 11.25. Found: C, 63.34; H, 4.63; N, 11.04

Synthesis of Fe(^{PhNH}PDI)(CO)$_2$ (2). In a 20 mL scintillation vial, Fe(^{PhNH}PDI)Cl_2 (**1**) (0.200 g, 0.321 mmol) was dissolved in 10 mL of THF and stirred for 10 minutes producing a dark brown solution. The solution was added to a 140 mL Fisher Porter tube equipped with a large stir bar. The Fisher Porter tube was placed in the liquid nitrogen cooled glovebox Coldwell to freeze the solution of Fe(^{PhNH}PDI)Cl_2 (**1**) in THF. Once frozen, 7.1 mL of 0.0944 molar solution of SmI_2 in THF was syringed into the tube dropwise and allowed to freeze. The tube was then capped with a head gauge, removed from the glovebox, charged with 40 psi of carbon monoxide, and allowed to thaw to room temperature whilst stirring. Once the contents of the tube melted, the dark brown solution became green while stirring overnight. The solvent was then removed *in vacuo* and redissolved in ~15 mL of Et_2O and filtered through an alumina plug. Slow evaporation of Et_2O yielded dark green X-ray quality crystals (^{PhNH}PDI)(CO)$_2$ (**2**) (0.125 g, 64.0% yield). FT-IR: ν_{NH} = 3373 cm^{-1}. $\nu_{C=O}$ = 1950, 1894 cm^{-1}, ^{1}H NMR (CD_2Cl_2) δ: 8.26, 8.25, 7.63, 7.61, 7.60, 7.36, 7.34, 7.33, 7.31, 7.23, 7.19, 7.14, 7.12, 7.06, 7.04, 6.96, 6.94, 5.08, 5.06, 2.49 ppm. ^{13}C NMR (CD_2Cl_2) δ: 217.10, 214.64, 211.46, 158.06, 157.98, 145.46, 145.33, 143.16, 143.04, 142.34, 142.25, 136.25, 135.84, 129.53, 126.93, 126.88, 124.11, 123.60, 122.42, 122.33, 121.87, 121.78, 120.60, 120.40, 119.83, 119.79, 118.28, 118.16, 115.03, 114.67, 15.68, 15.65 ppm. Mössbauer: δ = -0.083(3) mm/s; ΔE_Q = 1.337(5) mm/s. Anal. Calcd for $C_{35}H_{29}FeN_5O_2$: C, 69.20; H, 4.81; N, 11.53. Found: C, 69.32; H, 4.96; N, 11.32

Synthesis of [Fe(^{PhNH}PDI)(NO)$_2$][X] (X = BPh_4^- or PF_6^-) (3). In a 20 mL scintillation vial equipped with a stir bar, Fe(^{PhNH}PDI)(CO)$_2$ **(2)** (0.200 g, 0.321 mmol) and four equivalents of [$HNEt_3$][BPh_4] (0.555 g, 1.32 mmol) or [$HNEt_3$][PF_6] (0.205 g, 1.32 mmol) were dissolved in five mL of THF producing a green solution. In another vial, a stir bar and two equivalents of $NaNO_2$ (0.0454 g, 0.658 mmol) was dissolved in five mL of MeOH. Once the contents of the two vials were fully dissolved (requiring ~10 minutes of stirring) the $NaNO_2$ solution was added dropwise to the Fe(^{PhNH}PDI)(CO)$_2$ (**2**) and [$HNEt_3$][BPh_4] solution. The solution turned from green to maroon-red within 30 minutes of stirring. The solution was left to stir overnight, and the solvent was then removed *in vacuo.* The dark red solid was redissolved with 10 mL of CH_2Cl_2 and filtered through celite to remove $NaBPh_4$. The solution was layered with pentane for crystallization, resulting in a dark brown crystalline solid, [Fe(^{PhNH}PDI)(NO)$_2$]$^+$ (**3**$^+$) (0.0840 g, 42.7% yield). FT-IR: ν_{NH} = 3361 cm^{-1}. $\nu_{N=O}$ = 1792, 1718 cm^{-1}, ^{1}H NMR (CD_2Cl_2) δ: 7.38, 6.99, 3.67, 3.44, 2.27, 1.95, 1.82, 1.28, 1.16, 0.86 ppm. Mössbauer: δ = 0.35(1) mm/s; ΔE_Q = 0.89(2) mm/s. Anal. Calcd for $C_{57}H_{49}BFeN_7O_2$: C, 73.56; H, 5.31; N, 10.53. Found: C, 73.68; H, 5.48; N, 10.41

Reduction of [Fe(^{PhNH}PDI)(NO)$_2$][BPh_4] (3) to Fe(^{PhNH}PDI)(NO)$_2$ (4). In a 20 mL scintillation vial equipped with a stir bar, [Fe(^{PhNH}PDI)(NO)$_2$][BPh_4] (**3**) (0.0500 g, 0.0537 mmol) was dissolved in five mL of CH_2Cl_2, producing a dark red solution. The vial was capped with a size 33 Suba-seal septum. In another 20 mL vial, cobaltocene (0.0102 g, 0.0537 mmol) was dissolved in five mL of CH_2Cl_2. Both vials were left in a -35 °C freezer for 30 minutes. The vials were taken out of the freezer and the cobaltocene solution was immediately syringed into the [Fe(^{PhNH}PDI)(NO)$_2$]$^+$ (**3**$^+$) solution dropwise while stirring vigorously, producing a bright red solution. An initial solution phase FT-IR spectrum was collected to confirm the complete conversion of [Fe(^{PhNH}PDI)(NO)$_2$][BPh_4] (**3**) to Fe(^{PhNH}PDI)(NO)$_2$ (**4**). A color change from bright red to blue was observed over the course of the experiment. After confirming the decomposition of the nitrosyl peaks via solution phase FT-IR (typically ~60 minutes) the headspace was collected and analyzed via gas phase FT-IR. The solution was then removed *in vacuo*. The resulting blue solid was dissolved in ~15 mL of Et_2O and filtered through celite to remove all cobaltocenium. Slow evaporation yielded a blue solid that displays ν_{NH} in **Figure S15**. FT-IR: ν_{NH} = 3365 cm^{-1}.

Synthesis of Fe(^{PhNMe}PDI)Cl_2 (5). The procedure follows the same method for Fe(^{PhNH}PDI)Cl_2 (**1**) but instead of *N*-Phenyl-*o*-phenylenediamine, *N1*-methyl-*N*1-phenylbenzene-1,2-diamine (0.972 g, 4.90 mmol) was utilized in the reaction. The synthesis resulted in dark brown X-ray quality crystals Fe(^{PhNMe}PDI)Cl_2 (**5**) (1.27 g, 79.7% yield). FT-IR: $\nu_{C=N}$ = 1587 cm^{-1}. ^{1}H NMR (CD_2Cl_2) δ: 103.39, 37.35, 24.67, 23.90, 22.36, 22.08, 20.79, 19.62, 19.38, 7.94, 4.36, -35.41 ppm. Anal. Calcd for $C_{35}H_{33}Cl_2FeN_5$: C, 64.63; H, 5.11; N, 10.77. Found: C, 64.44; H, 5.18; N, 10.97

Synthesis of Fe(^{PhNMe}PDI)(CO)$_2$ (6). The procedure follows the same method for Fe(^{PhNH}PDI)(CO)$_2$ (**2**) but instead of Fe(^{PhNH}PDI)Cl$_2$ (**1**), Fe(^{PhNMe}PDI)Cl$_2$ (**5**) (0.200 g, 0.307 mmol) was utilized. Slow evaporation of the resultant Fe(^{PhNMe}PDI)Cl$_2$ (**5**) in Et$_2$O yielded dark green crystals of Fe(^{PhNMe}PDI)(CO)$_2$ (**6**) (0.124 g, 63.8% yield). Fe(^{PhNMe}PDI)(CO)$_2$ (**6**) was used without any further purification in the synthesis of [Fe(^{PhNMe}PDI)(NO)$_2$]$^+$ (**7**$^+$). FT-IR: $\nu_{C≡O}$ = 1952, 1882 cm^{-1}.

Synthesis of [Fe(^{PhNMe}PDI)(NO)$_2$][X] (X = BPh$_4^-$ or PF$_6^-$) (**7**). The procedure follows the same method for [Fe(^{PhNH}PDI)(NO)$_2$]$^+$ **(3$^+$)** but instead of adding Fe(^{PhNH}PDI)(CO)$_2$ (**2**), Fe(^{PhNMe}PDI)(CO)$_2$ (**6**) (0.200 g, 0.315 mmol) was utilized. The solution was layered with pentane for crystallization, producing a yellow-orange crystalline solid [Fe(^{PhNMe}PDI)(NO)$_2$]$^+$ (**7**$^+$) (0.175 g, 87.1% yield). FT-IR: $\nu_{N=O}$ = 1799, 1726 cm^{-1}. ^{1}H NMR (CD$_2$Cl$_2$) δ: 7.36, 7.00, 6.84, 6.62, 3.44, 2.12, 1.50, 1.30, 1.16, 0.89, 0.81 ppm. Mössbauer: δ = 0.45(3) mm/s; ΔE_Q = 1.05(6) mm/s. Anal. Calcd for C$_{59}$H$_{53}$BFeN$_7$O$_2$: C, 73.91; H, 5.57; N, 10.23. Found: C, 74.05; H, 5.54; N, 10.21

Reduction of [Fe(^{PhNMe}PDI)(NO)$_2$][BPh$_4$] (7) to form Fe(^{PhNMe}PDI)(NO)$_2$ (8). The procedure follows the same method for the reduction of [Fe(^{PhNH}PDI)(NO)$_2$][BPh$_4$] (**3**), but instead of adding [Fe(^{PhNH}PDI)(NO)$_2$][BPh$_4$] (**3**), [Fe(^{PhNMe}PDI)(NO)$_2$][BPh$_4$] (**7**) (0.05 g, 0.0782 mmol) was utilized. The solution was removed *in vacuo* two hours into the reaction. The red solid was filtered through celite with about 15 mL of Et$_2$O. Slow evaporation yielded an orange solid of Fe(^{PhNMe}PDI)(NO)$_2$ (**8**) (0.0204 g, 40.8% yield). FT-IR: $\nu_{N=O}$ = 1686, 1636 cm^{-1}. ^{1}H NMR (CD$_2$Cl$_2$) δ: 7.87, 7.85, 7.64, 7.63, 7.61, 7.28, 7.14, 7.08, 6.82, 6.80, 6.72, 6.50, 6.63, 3.15, 2.22 ppm. ^{13}C NMR (CD$_2$Cl$_2$) δ: 167.51, 155.53, 149.33, 148.26, 138.29, 136.85, 136.36, 129.31, 128.96, 128.44, 128.37, 127.72, 126.39, 124.99, 122.52, 120.86, 119.36, 117.80, 116.20, 114.57, 113.87, 79.52, 74.97, 66.07, 48.70, 40.86, 38.90, 30.10, 16.81, 15.51, 1.18 ppm. Anal. Calcd for C$_{35}$H$_{33}$FeN$_7$O$_2$: C, 65.73; H, 5.20; N, 15.33. Found: C, 65.31; H, 5.65; N, 15.11

Synthesis of ^{15}N Isotope [Fe(^{PhNH}PDI)(^{15}NO)$_2$][BPh$_4$] (3-^{15}N). The procedure follows the same method for the synthesis of [Fe(^{PhNH}PDI)(NO)$_2$][BPh$_4$] (**3**), but instead of adding NaNO$_2$, Na^{15}NO$_2$ was utilized. The dark red solid was redissolved with about 10 mL of CH$_2$Cl$_2$ and filtered through celite to remove NaBPh$_4$. The solution was layered with pentane for crystallization, resulting in a dark brown crystalline solid [Fe(^{PhNH}PDI)(^{15}NO)$_2$][BPh$_4$] (**3-^{15}N).** FT-IR: ν_{NH} = 3356 cm^{-1}, $\nu_{N=O}$ = 1755, 1687 cm^{-1}

Reduction of ^{15}N Isotope [Fe(^{PhNH}PDI)(^{15}NO)$_2$][BPh$_4$] (**3-^{15}N**). The procedure follows the same method for the reduction of [Fe(^{PhNH}PDI)(NO)$_2$][BPh$_4$] (**3**) to Fe(^{PhNH}PDI)(NO)$_2$ (**4**), but instead of adding [Fe(^{PhNH}PDI)(NO)$_2$][BPh$_4$] (**3**), [Fe(^{PhNH}PDI)(^{15}NO)$_2$][BPh$_4$] (**3-^{15}N**) (0.0500 g, 0.0537 mmol) was utilized. The solution was removed *in vacuo* after confirming the decomposition of the nitrosyl peaks via solution phase IR. This could also be confirmed via color change from red to blue. The blue solid was filtered through celite with about 15 mL of Et$_2$O. Slow evaporation yielded blue solid that displays ν_{NH}. FT-IR: ν_{NH} = 3373 cm^{-1}.

Synthesis of Fe(^{MeNH}PDI)Cl$_2$ (11). The procedure follows the same method for Fe(^{PhNH}PDI)Cl$_2$ (**1**) utilizing *N*-Methyl-1,2-phenylenediamine (0.598 g, 4.90 mmol) in the place of *N*-Phenyl-*o*-phenylenediamine. The procedure resulted in dark brown X-ray quality crystals of Fe(^{MeNH}PDI)Cl$_2$ (**11**) (1.20 g, 98.3% yield). FT-IR: ν_{NH} = 3315 cm^{-1}, $\nu_{C=N}$ = 1583 cm^{-1}. ^{1}H NMR (CD$_2$Cl$_2$) δ: 32.82, 21.61, 20.04, 15.22, 12.43, 9.56, 4.57, 3.43, 1.84, 1.14, -6.67, -10.43, -13.15, -13.61, -18.13, -19.14, -39.30 ppm. Anal. Calcd for C$_{23}$H$_{25}$ Cl$_2$FeN$_5$: C, 55.45; H, 5.06; N, 14.06. Found: C, 55.66; H, 4.99; N, 14.15

Synthesis of Fe(^{MeNH}PDI)(CO)$_2$ (**12**). The procedure follows the same method for Fe(^{PhNH}PDI)(CO)$_2$ (**2**) utilizing Fe(^{MeNH}PDI)Cl$_2$ (**11**) (0.200 g, 0.401 mmol) in the place of Fe(^{PhNH}PDI)Cl$_2$ (**1**). Slow evaporation of the resultant green solution in Et$_2$O yielded dark green crystals of Fe(^{MeNH}PDI)(CO)$_2$ (**12**) (0.132g, 68.3% yield). FT-IR: ν_{NH} = 3385 cm^{-1}, $\nu_{C=O}$ = 1950, 1890 cm^{-1}. ^{1}H NMR (CD$_2$Cl$_2$) δ: 8.24, 8.22, 7.60, 7.59, 7.57, 7.22, 7.20, 7.19, 6.97, 6.95, 6.94, 6.77, 6.76, 6.74, 3.46, 3.44, 3.43, 3.42, 3.12, 2.73, 2.72, 2.70, 2.38, 1.27, 1.17, 1.16, 1.14 ppm. ^{13}C NMR (CD$_2$Cl$_2$) δ: 216.48, 214.59, 212.65, 158.01, 145.09, 141.48, 141.26, 141.18, 127.31, 122.83, 122.70, 121.42, 121.32, 117.60, 117.56, 116.62, 116.48, 110.88, 66.05, 30.70, 15.50, 15.37, 15.32 ppm. Anal. Calcd for $C_{25}H_{25}FeN_5O_2$: C, 62.12; H, 5.21; N, 14.49. Found: C, 62.29; H, 5.40; N, 14.23

Synthesis of [Fe(^{MeNH}PDI)(NO)$_2$][X] (X = BPh$_4^-$ or PF$_6^-$) (**13**). The procedure follows the same method for [Fe(^{PhNH}PDI)(NO)$_2$]$^+$ (**3**$^+$) utilizing Fe(^{MeNH}PDI)(CO)$_2$ (**12**) (0.200 g, 0.414 mmol) in the place of Fe(^{PhNH}PDI)(CO)$_2$ (**2**). The resultant dark red solution was layered with pentane for crystallization, producing dark red crystalline [Fe(^{MeNH}PDI)(NO)$_2$]$^+$ (**13**$^+$) (0.102 g, 50.6% yield). FT-IR: ν_{NH} = 3425 cm^{-1}, $\nu_{N=O}$ = 1791, 1719 cm^{-1}, ^{1}H NMR (CD$_2$Cl$_2$) δ: 8.72, 8.15, 7.98, 6.93, 6.35, 6.20, 5.58, 5.50, 3.68, 3.43, 2.58, 2.54, 1.96, 1.81, 1.30, 1.15, 0.88 ppm. Anal. Calcd for $C_{23}H_{25}FeF_6PN_7O_2$: C, 43.69; H, 3.99; N, 15.51. Found: C, 43.59; H, 4.05; N, 14.69

Reduction of [Fe(^{MeNH}PDI)(NO)$_2$][BPh$_4$] (13) to form Fe (^{MeNH}PDI)(NO)$_2$ (**14**). The procedure follows the same method for the in-situ formation of Fe(^{PhNH}PDI)(NO)$_2$ (**4**) utilizing [Fe(^{MeNH}PDI)(NO)$_2$][BPh$_4$] (**13**) (0.05 g, 0.0620 mmol) in the place of [Fe(^{PhNH}PDI)(NO)$_2$][BPh$_4$] (**3**). The solvent was removed *in vacuo* after confirming the decomposition of the nitrosyl peaks via solution phase FT-IR. This was also confirmed via color change from red to blue and the detection of N$_2$O via gas phase analysis of the headspace of the reaction. The blue solid was dissolved in 15 mL of Et$_2$O and filtered through celite. Slow evaporation yielded a blue solid (0.0175 g, 35.0% yield) that displays ν_{NH}. FT-IR: ν_{NH} = 3360 cm^{-1}.

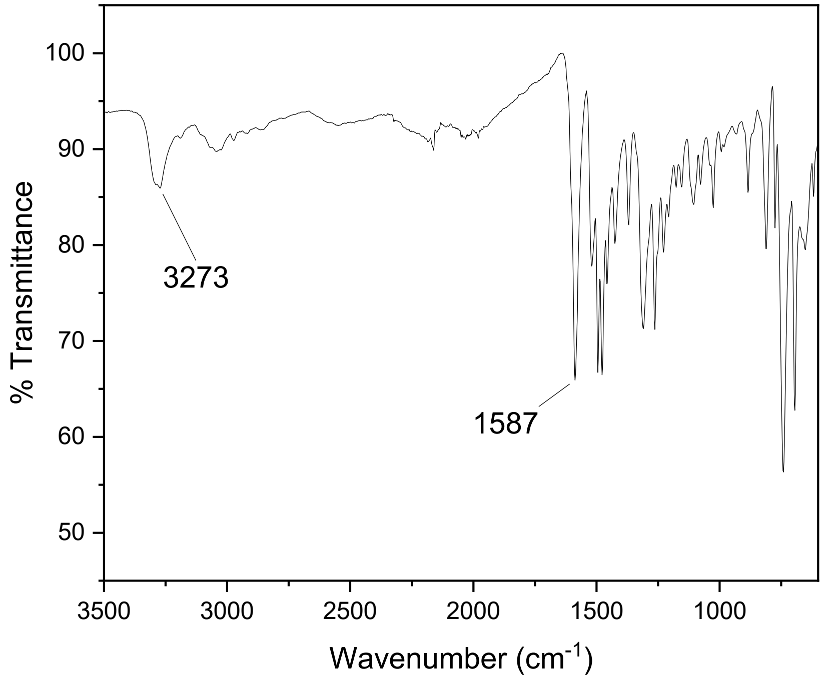

Figure S1. ATR FT-IR spectrum of Fe(^{PhNH}PDI)Cl_2 (**1**).

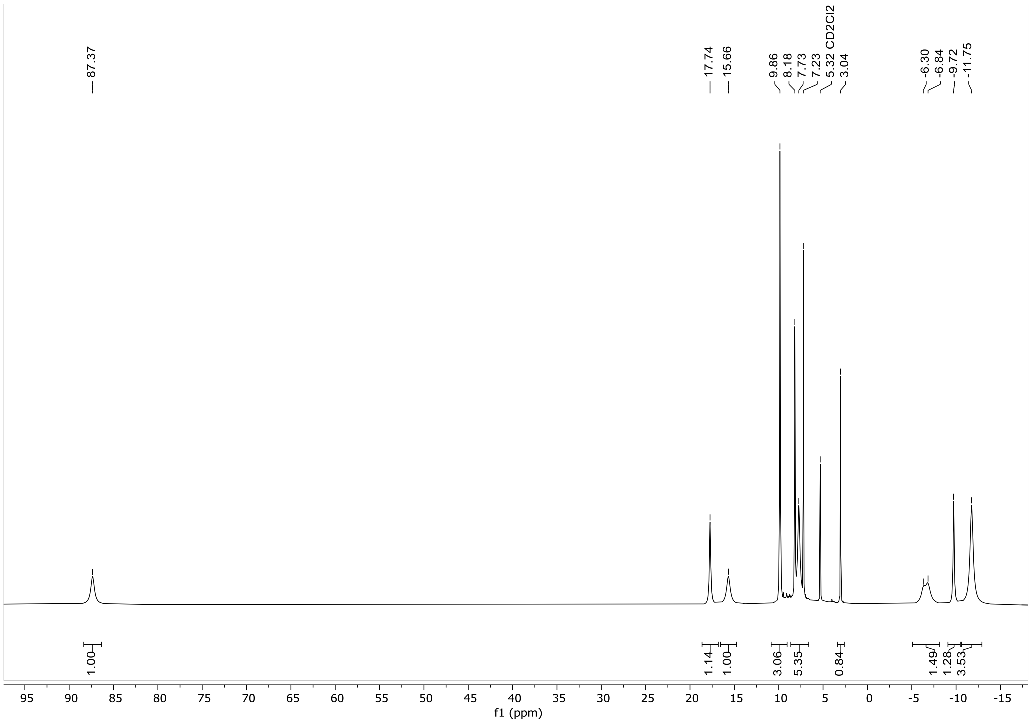

Figure S2. ^{1}H NMR spectrum of Fe(^{PhNH}PDI)Cl_2 (**1**) in CD_2Cl_2.

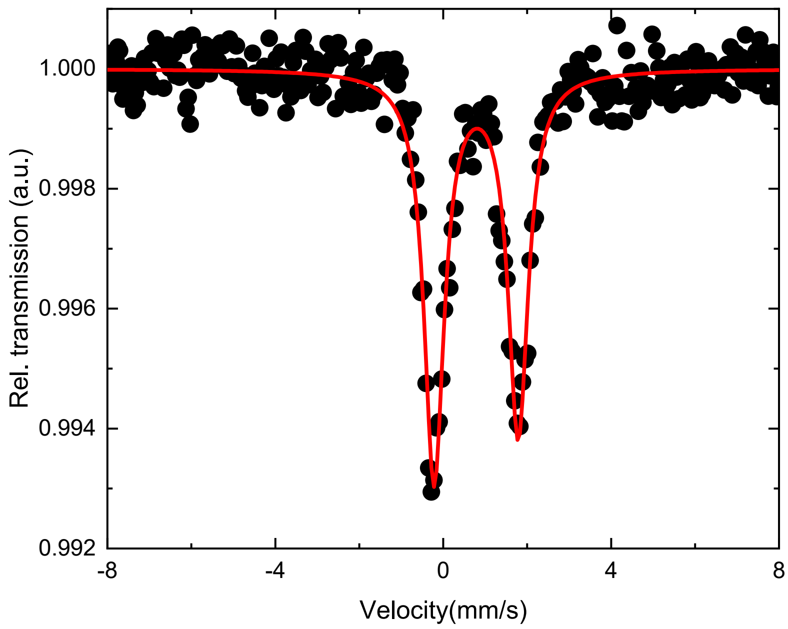

Figure S3. Zero-field Mössbauer spectrum of Fe(^{PhNH}PDI)Cl_2 (**1**). $\delta = 0.886(6)$ mm/s; $\Delta E_Q = 2.00(1)$

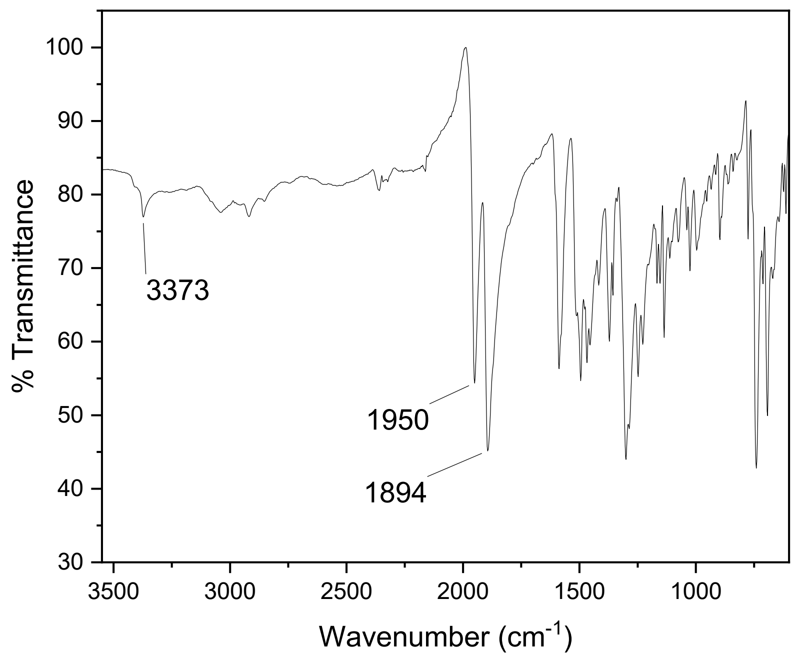

Figure S4. ATR FT-IR spectrum of Fe(^{PhNH}PDI)$(CO)_2$ (**2**).

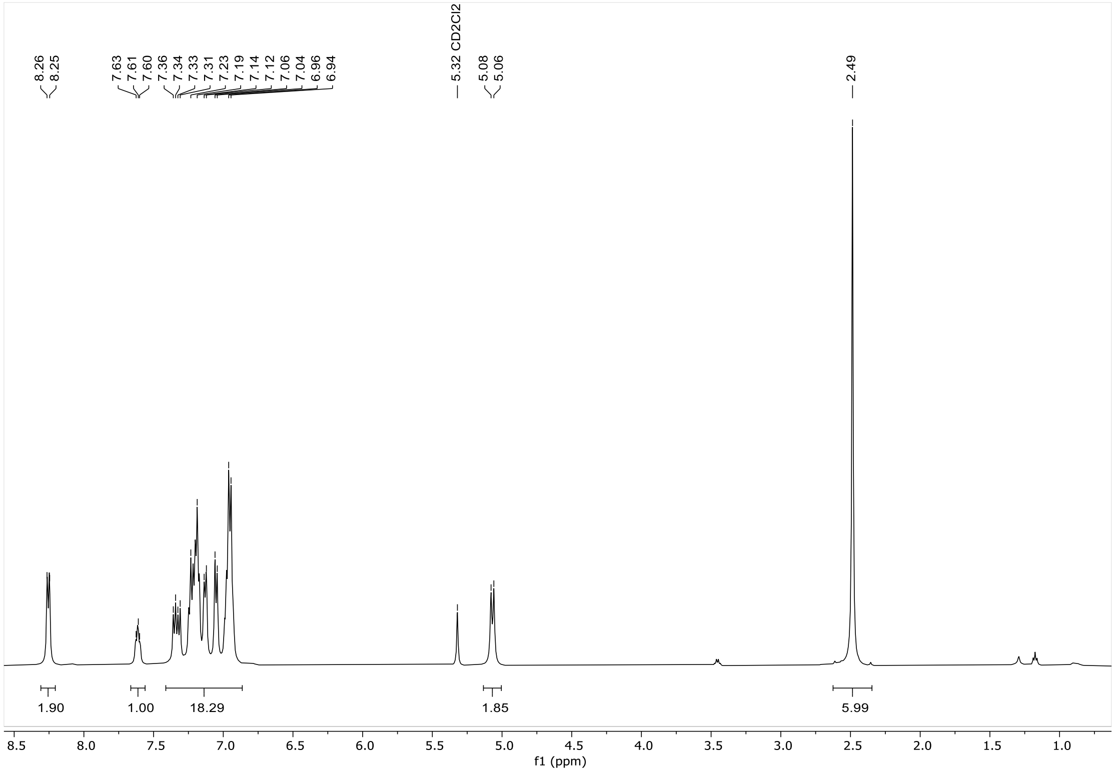

Figure S5. ^{1}H NMR spectrum of Fe(^{PhNH}PDI)(CO)$_2$ (**2**) in CD_2Cl_2.

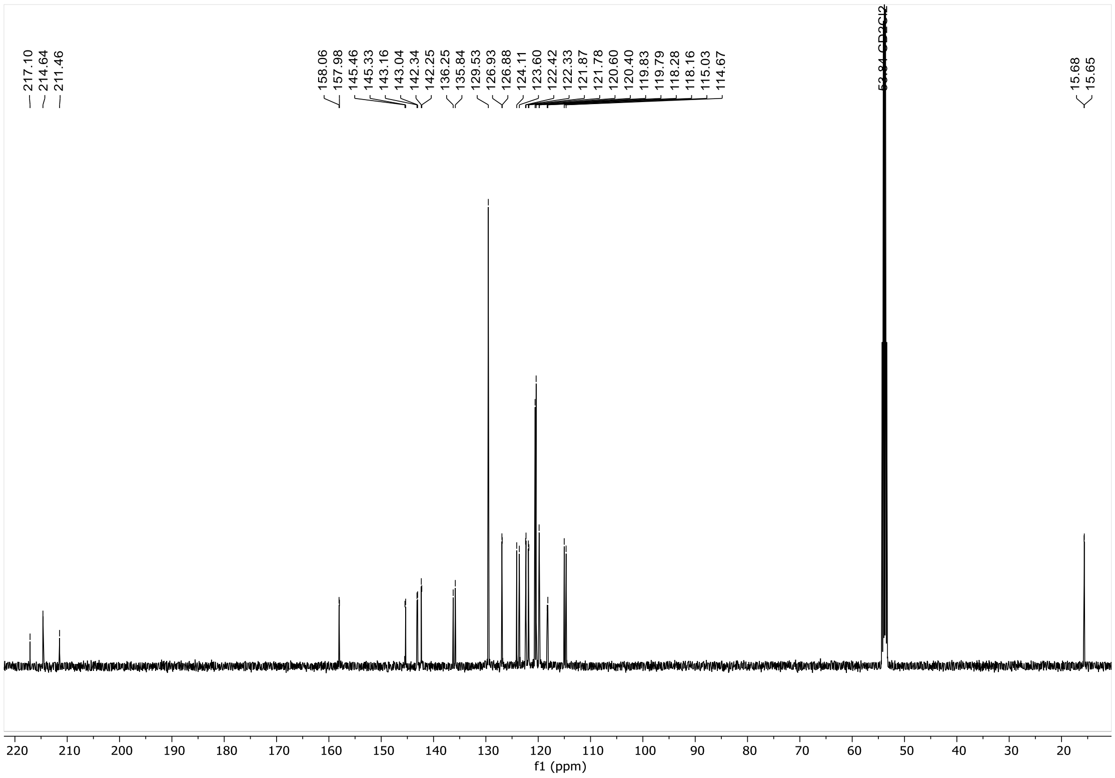

Figure S6. ^{13}C NMR spectrum of Fe(^{PhNH}PDI)(CO)$_2$ (**2**) in CD_2Cl_2.

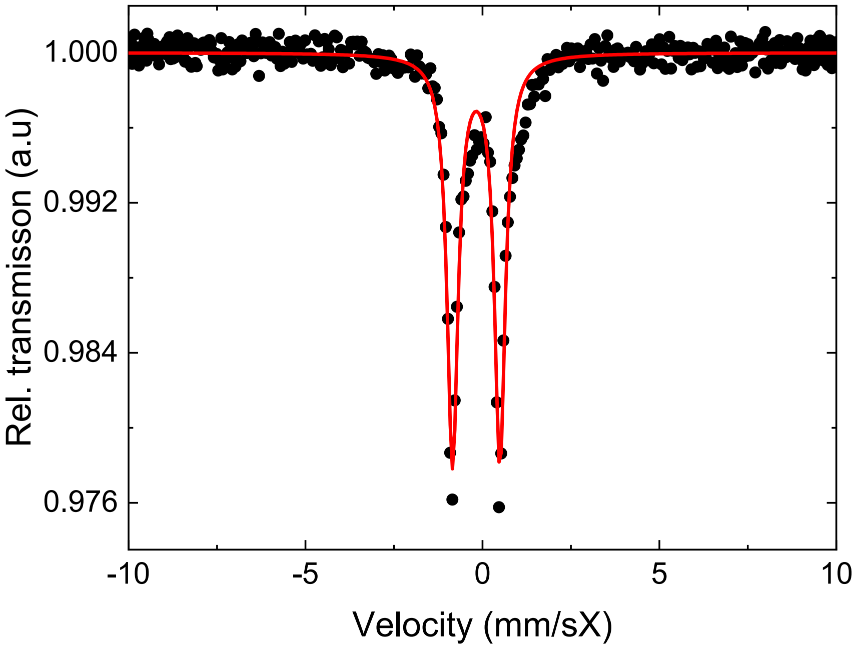

Figure S7. Zero-field Mössbauer spectrum of Fe(^{PhNH}PDI)(CO)$_2$ (**2**). δ = -0.083(3) mm/s; ΔE_Q = 1.337(5).

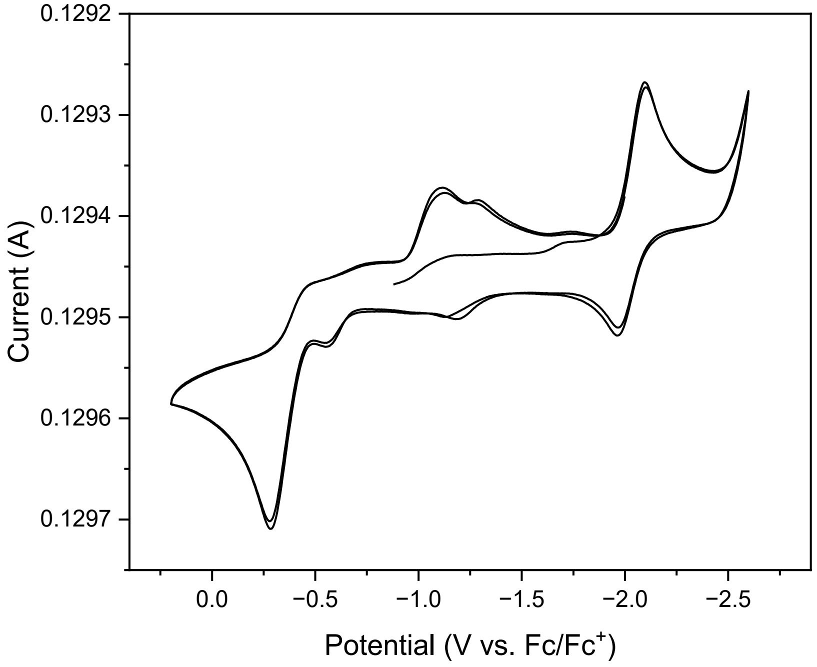

Figure S8. Cyclic voltammetry of Fe(^{PhNH}PDI)(CO)$_2$ (**2**). 1 mM in MeCN; glassy carbon WE, Pt wire CE, and Ag/AgNO$_3$ in MeCN RE, 100 mM TBAPF$_6$ electrolyte.

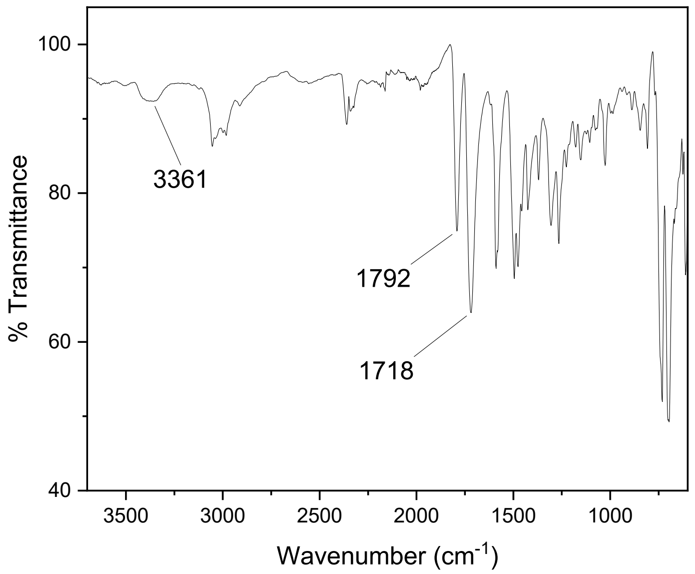

Figure S9. ATR FT-IR spectrum of $[Fe(^{PhNH}PDI)(NO)_2][BPh_4]$ (**3**).

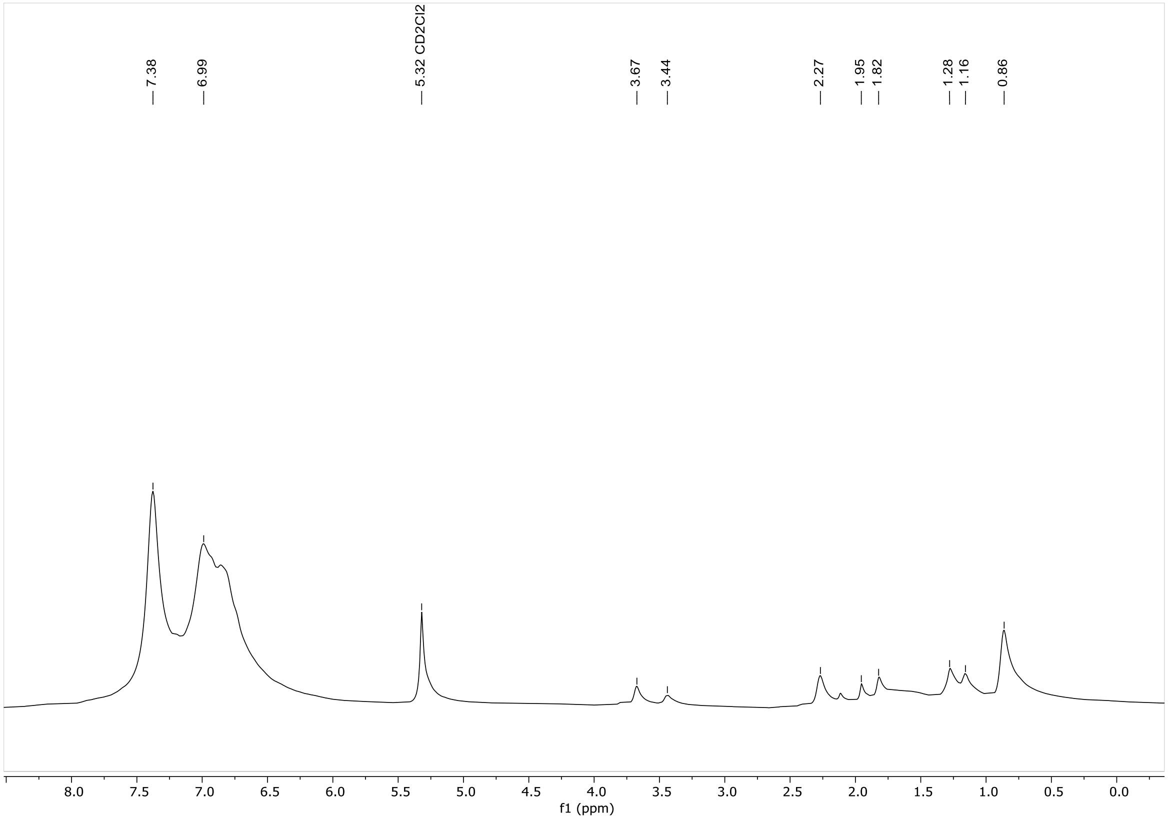

Figure S10. ^1H NMR spectrum of $[Fe(^{PhNH}PDI)(NO)_2][BPh_4]$ (**3**) in CD_2Cl_2.

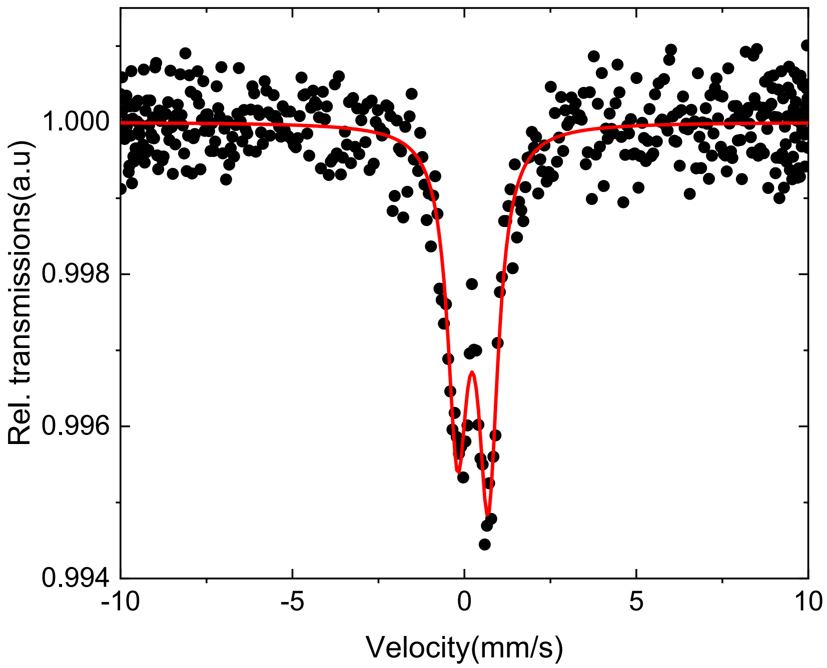

Figure S11. Zero-field Mössbauer spectrum of $[Fe(^{PhNH}PDI)(NO)_2][BPh_4]$ (**3**). $\delta = 0.35(1)$ mm/s; $\Delta E_Q = 0.89(2)$

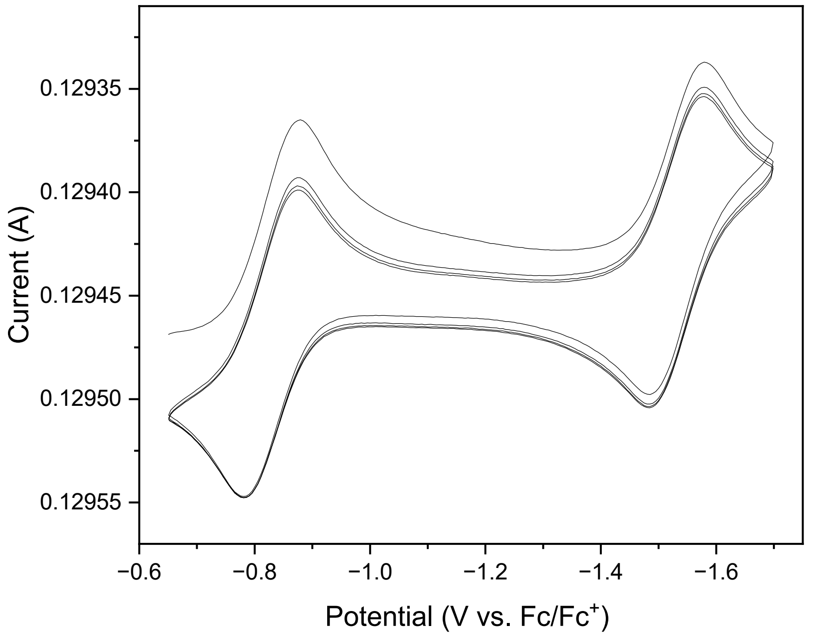

Figure S12. Cyclic voltammetry of $[Fe(^{PhNH}PDI)(NO)_2][BPh_4]$ (**3**). 1 mM in MeCN; glassy carbon WE, Pt wire CE, and $Ag/AgNO_3$ in MeCN RE, 100 mM $TBAPF_6$ electrolyte.

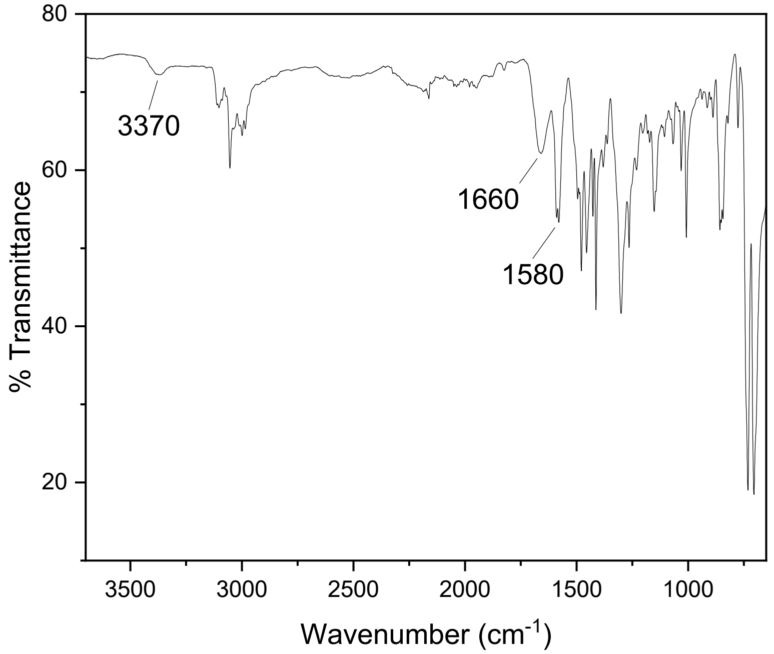

Figure S13. ATR FT-IR spectrum of the crude reaction mixture from the N-N coupling reaction of [Fe(^{PhNH}PDI)(NO)$_2$][BPh$_4$] (**4**).

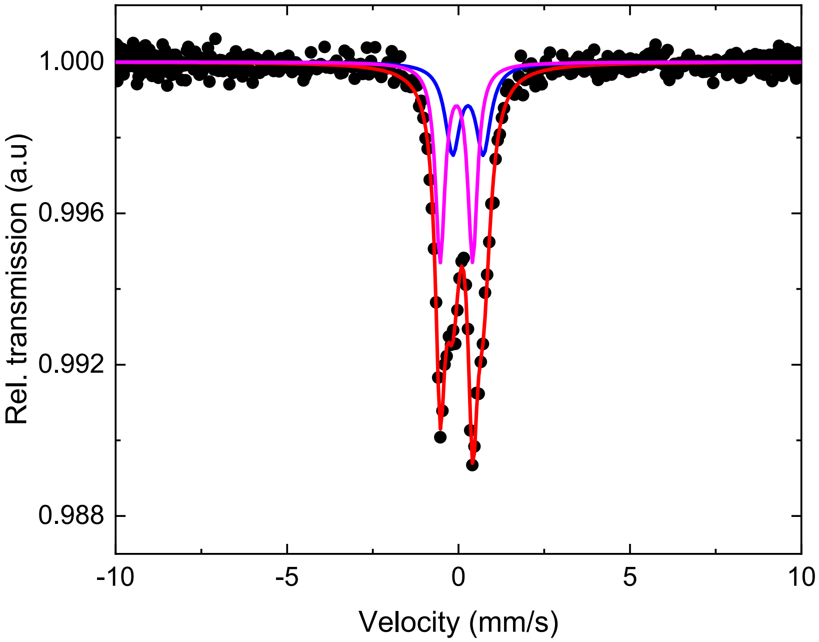

Figure S14. Zero-field Mössbauer spectrum of crude reaction mixture from the N-N coupling reaction of [Fe(^{PhNH}PDI)(NO)$_2$][BPh$_4$] (**4**). Pink curve (52%): $\delta = 0.38(1)$ mm/s; $\Delta E_Q = 0.895(8)$. Blue curve (48%): $\delta = 0.040(4)$ mm/s; $\Delta E_Q = 0.946(5)$.

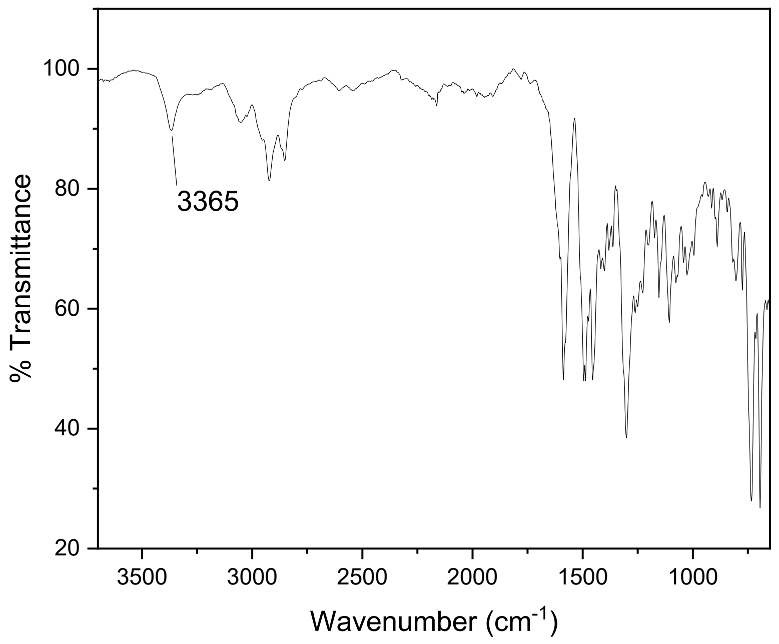

Figure S15. ATR FT-IR spectrum of the blue product from the N-N coupling reaction of $[Fe(^{PhNH}PDI)(NO)_2][BPh_4]$ (**4**) post filtration.

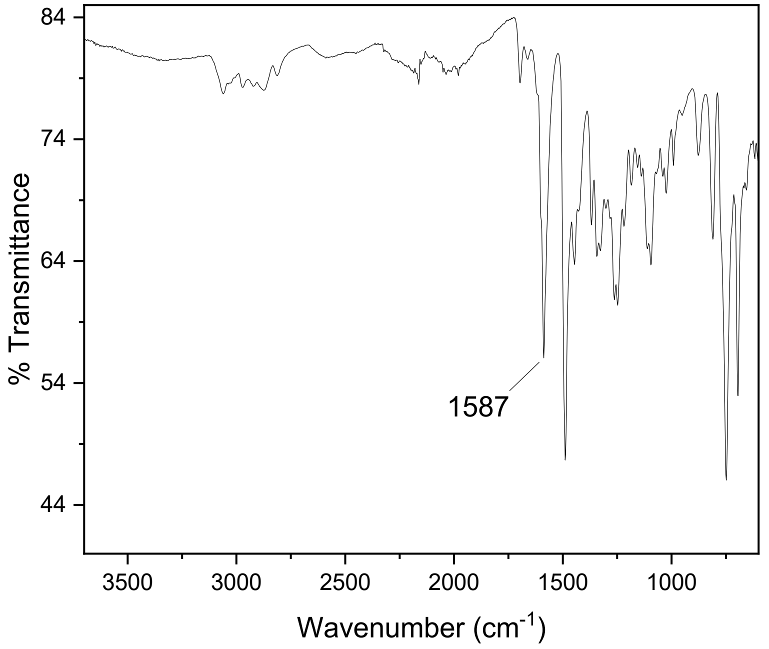

Figure S16. ATR FT-IR spectrum of $Fe(^{PhNMe}PDI)Cl_2$ (**5**)

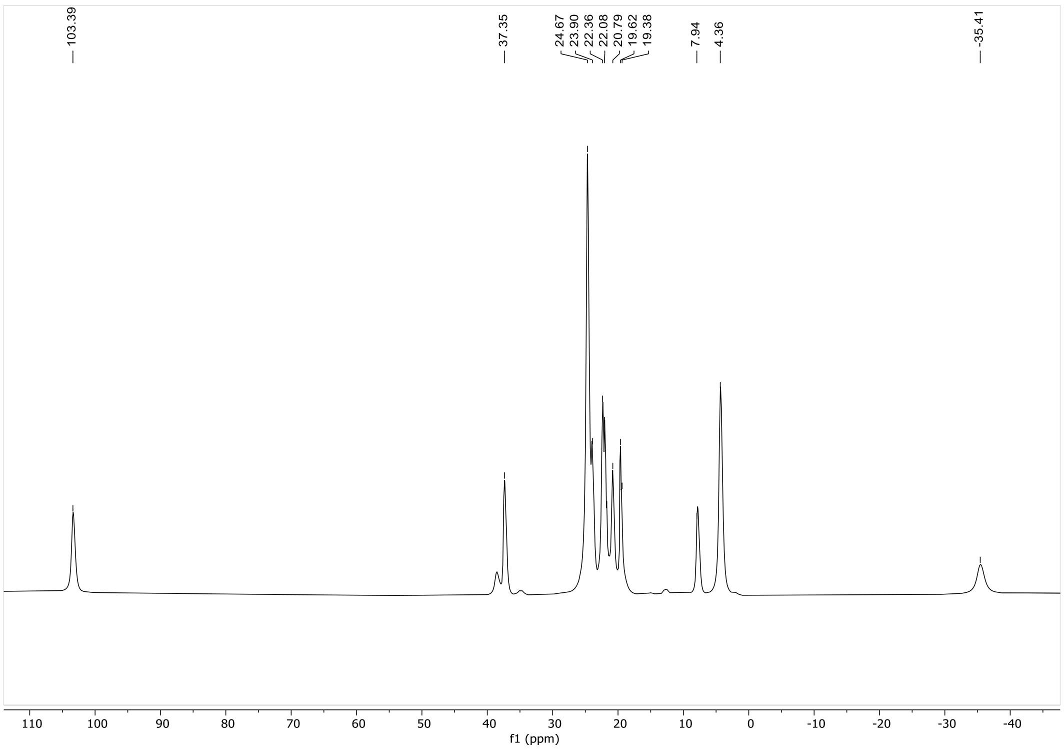

Figure S17. ^{1}H NMR spectrum of Fe(^{PhNMe}PDI)Cl_2 (**5**) in CD_2Cl_2.

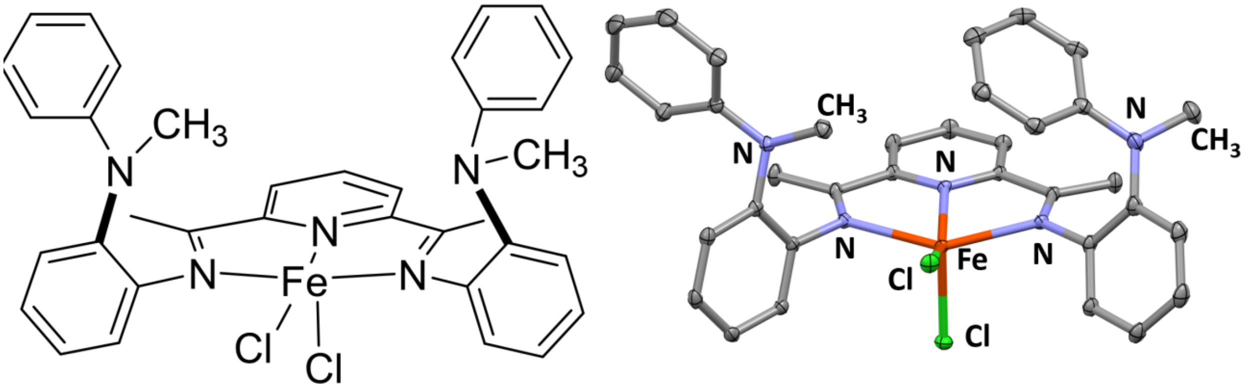

Figure S18. Chemdraw (left) and solid state structure (right) of Fe(^{PhNMe}PDI)Cl_2 (**5**). Selected bond lengths (Å) and angles (°): Fe(1)–N(1) 2.253(1), Fe(1)–N(2) 2.122(1), Fe(1)–N(3) 2.254(2), Fe(1)–Cl(1) 2.3372(5), Fe(1)-Cl(2) 2.2796(5), N(1)–C(2) 1.285(2), N(3)–C(8) 1.285(2), C(2)-C(3) 1.488(3), C(7)-C(8) 1.490(2), and N(1)–Fe(1)-N(3) 144.25(5), N(2)–Fe(1)-Cl(1) 151.35(4).

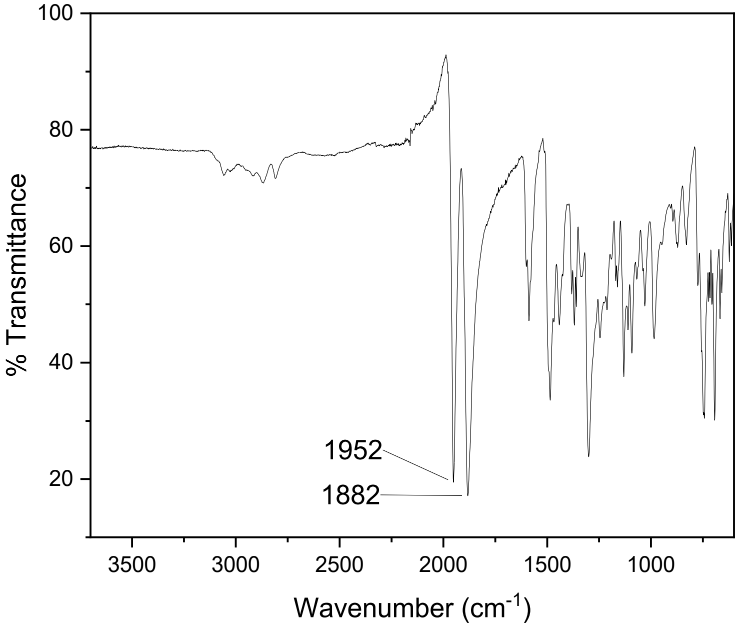

Figure S19. ATR FT-IR spectrum of Fe(^{PhNMe}PDI)(CO)$_2$ (**6**).

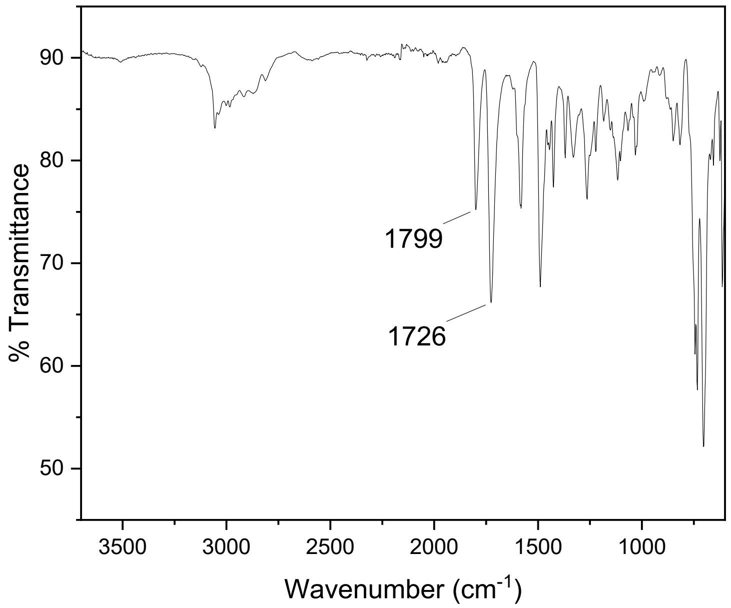

Figure S20. ATR FT-IR spectrum of [Fe(^{PhNMe}PDI)(NO)$_2$][BPh$_4$] (**7**).

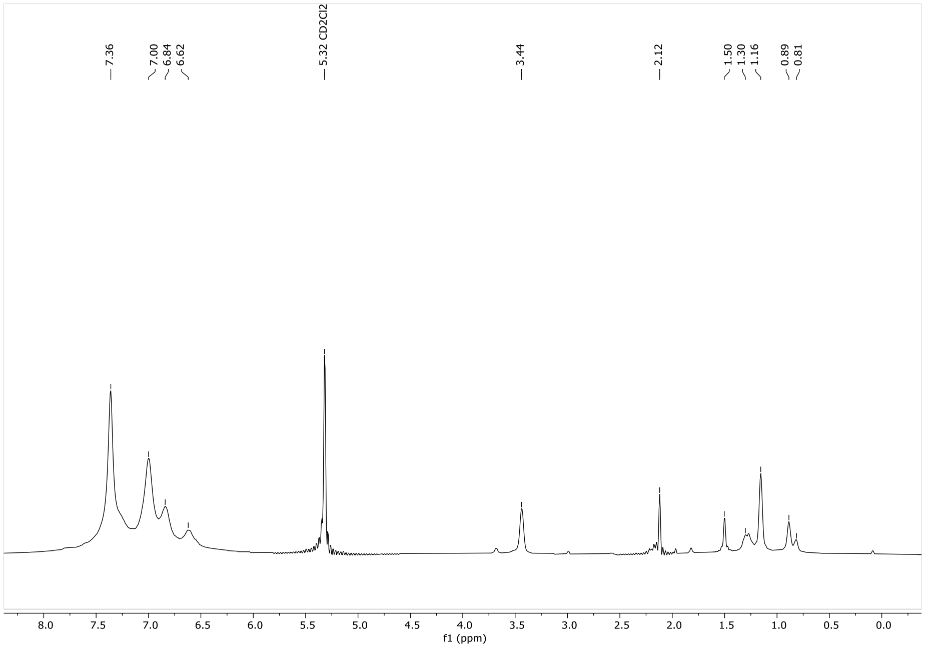

Figure S21. ^{1}H NMR spectrum of $[Fe(^{PhNMe}PDI)(NO)_2][BPh_4]$ (**7**) in CD_2Cl_2.

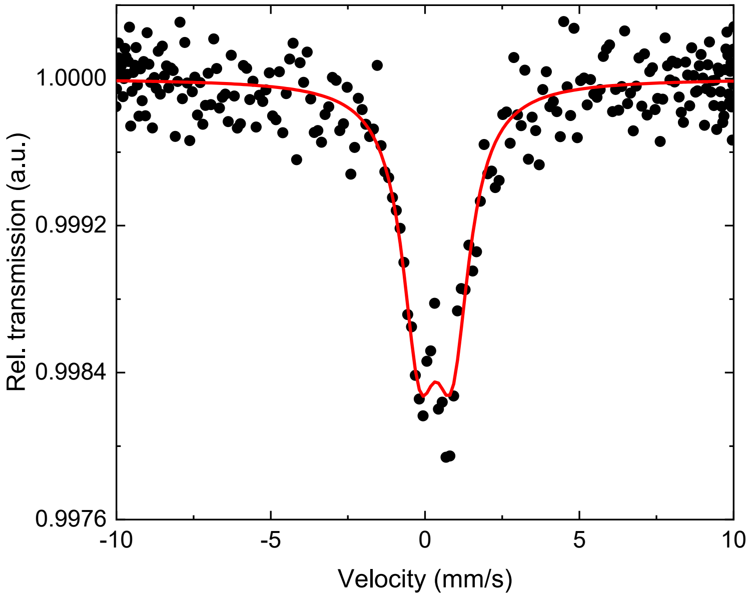

Figure S22. Zero-field Mössbauer spectrum of $[Fe(^{PhNMe}PDI)(NO)_2][BPh_4]$ (**7**). $\delta = 0.45(3)$ mm/s; $\Delta E_Q = 1.05(6)$

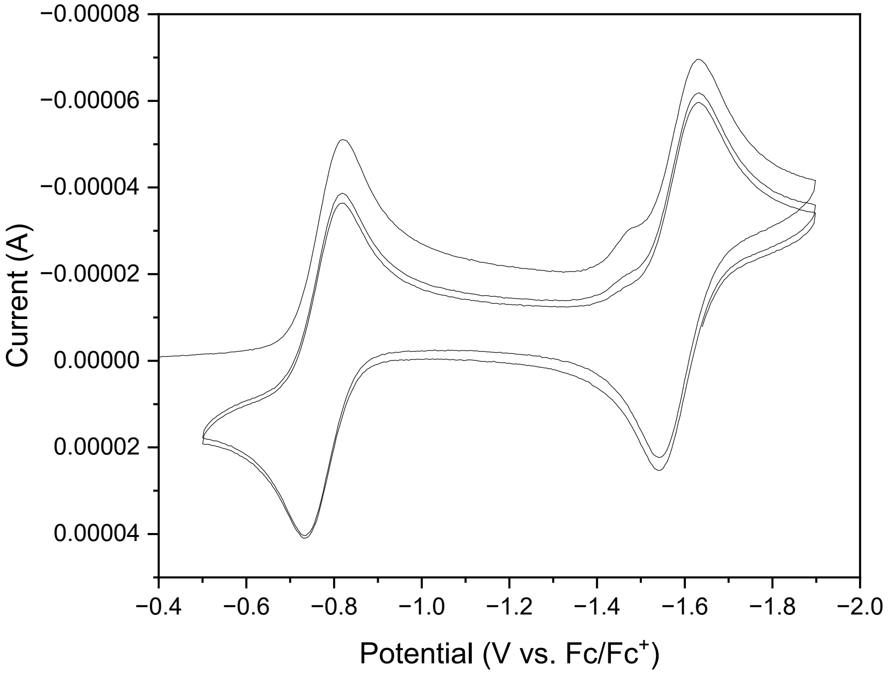

Figure S23. Cyclic voltammetry of [Fe(^{PhNMe}PDI)(NO)$_2$][BPh$_4$] (**7**). 1 mM in MeCN; glassy carbon WE, Pt wire CE, and Ag/AgNO$_3$ in MeCN RE, 100 mM TBAPF$_6$ electrolyte.

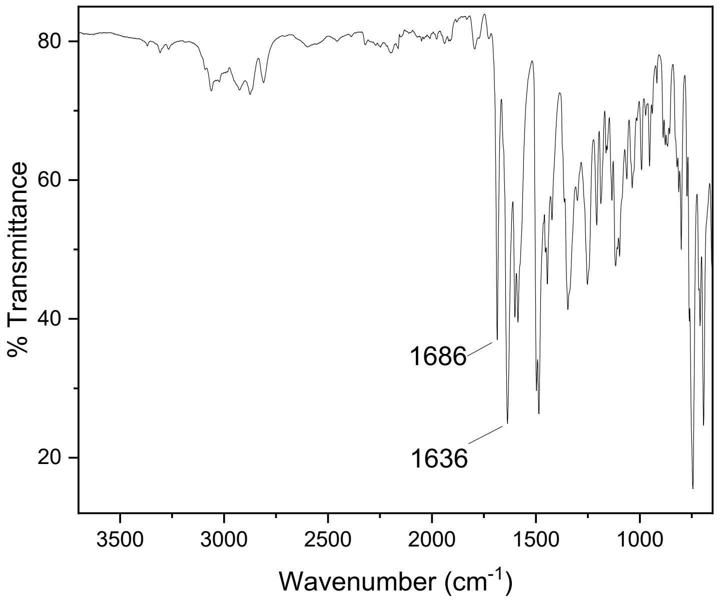

Figure S24. ATR FT-IR spectrum of Fe(^{PhNMe}PDI)(NO)$_2$ (**8**)

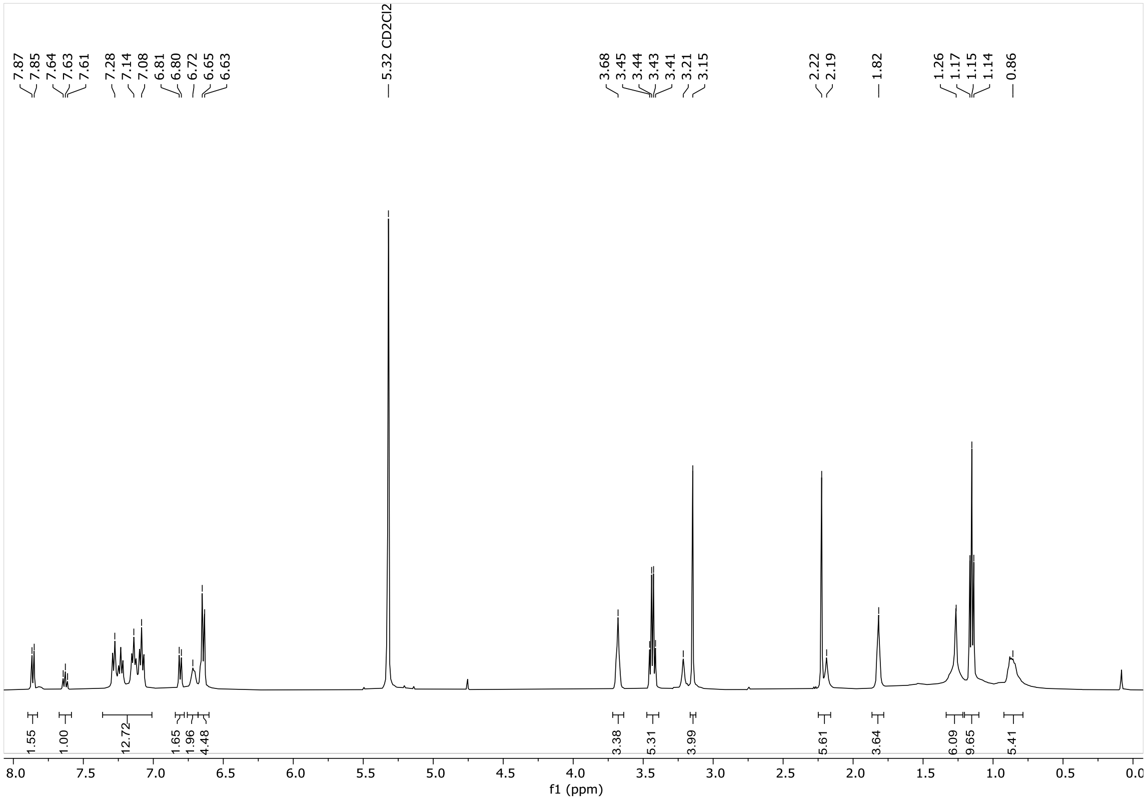

Figure S25. ^{1}H NMR spectrum of Fe(^{PhNMe}PDI)$(NO)_2$ (**8**) in CD_2Cl_2.

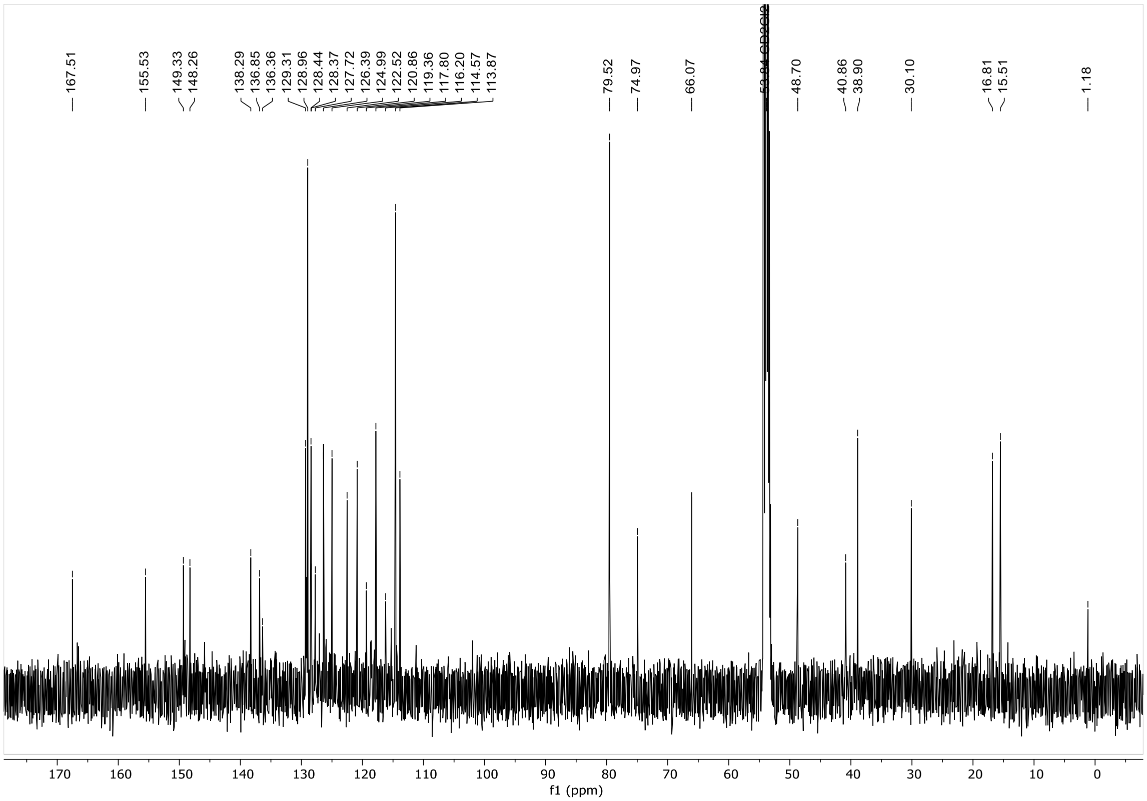

Figure S26. ^{13}C NMR Spectrum of Fe(^{PhNMe}PDI)$(NO)_2$ (**8**) in CD_2Cl_2.

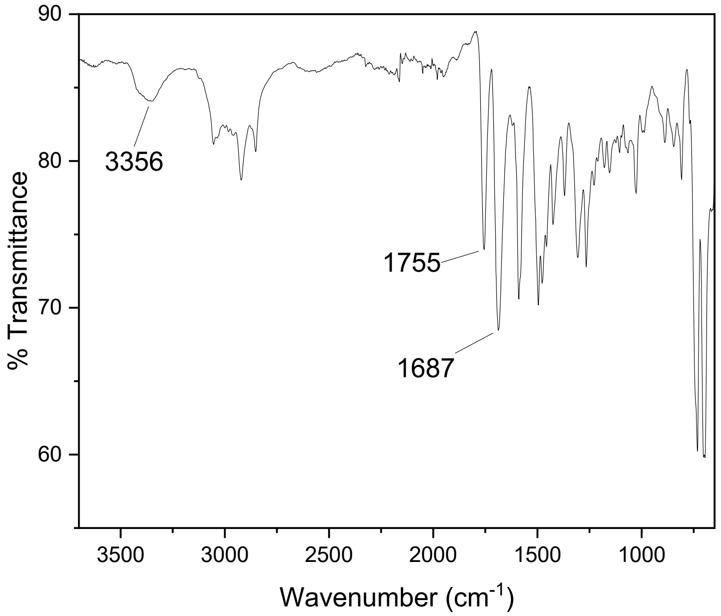

Figure S27. ATR FT-IR spectrum of $[Fe(^{PhNH}PDI)(^{15}NO)_2][BPh_4]$ (**3-^{15}N**).

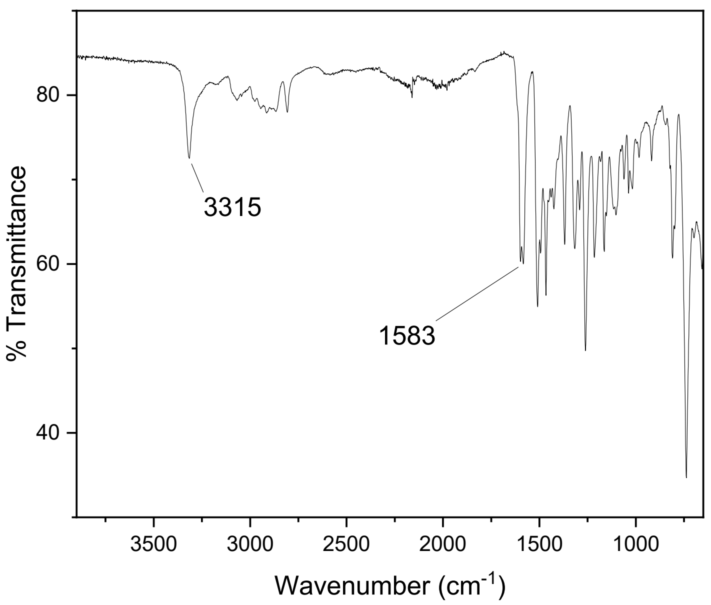

Figure S28. ATR FT-IR spectrum of $Fe(^{MeNH}PDI)Cl_2$ (**11**).

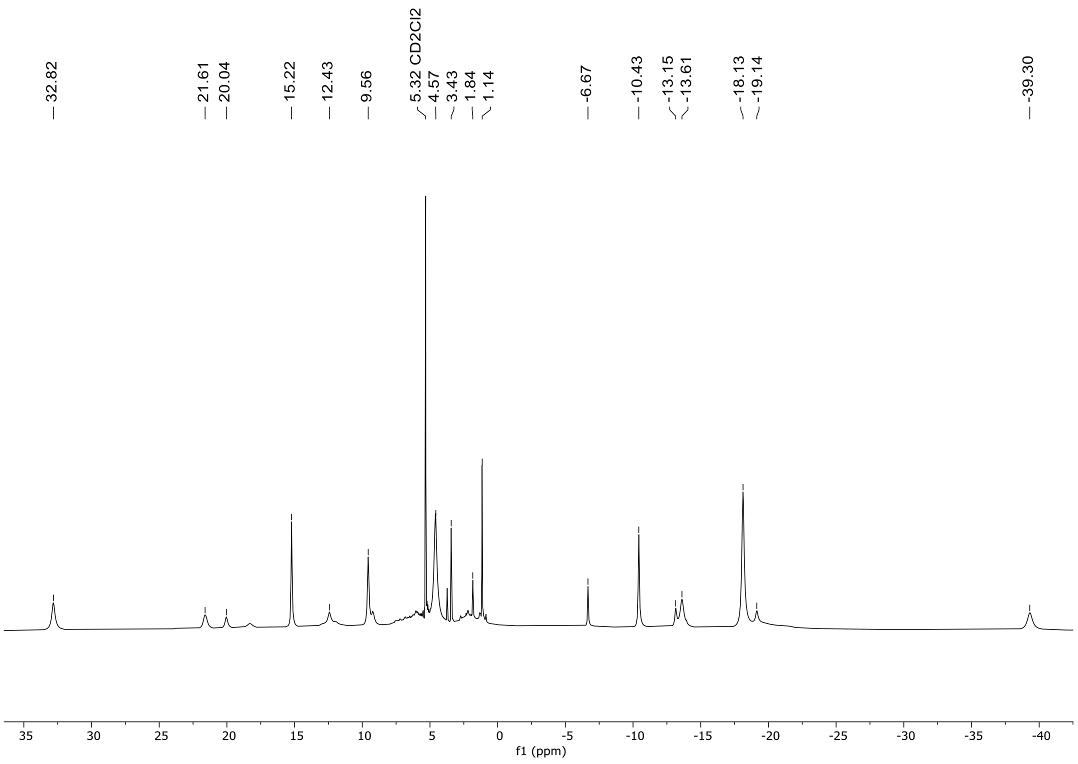

Figure S29. ${}^{1}H$ NMR spectrum of $Fe(^{MeNH}PDI)Cl_2$ (**11**) in CD_2Cl_2.

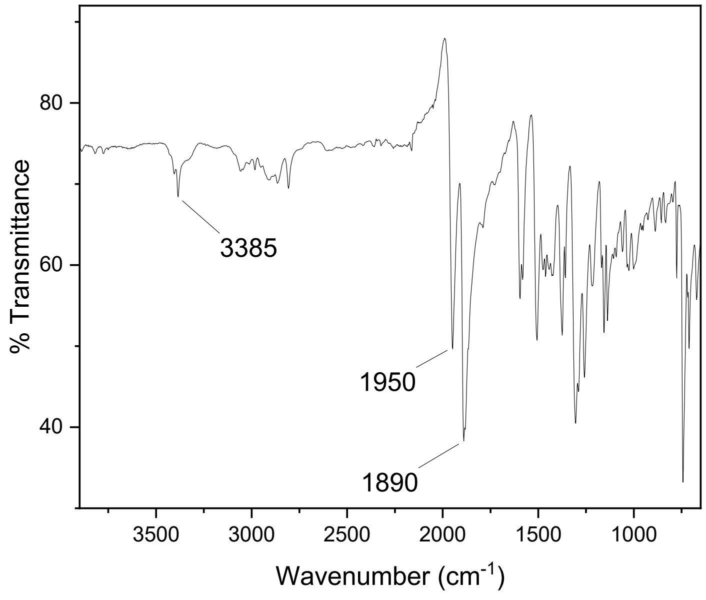

Figure S30. ATR FT-IR spectrum of $Fe(^{MeNH}PDI)(CO)_2$ (**12**).

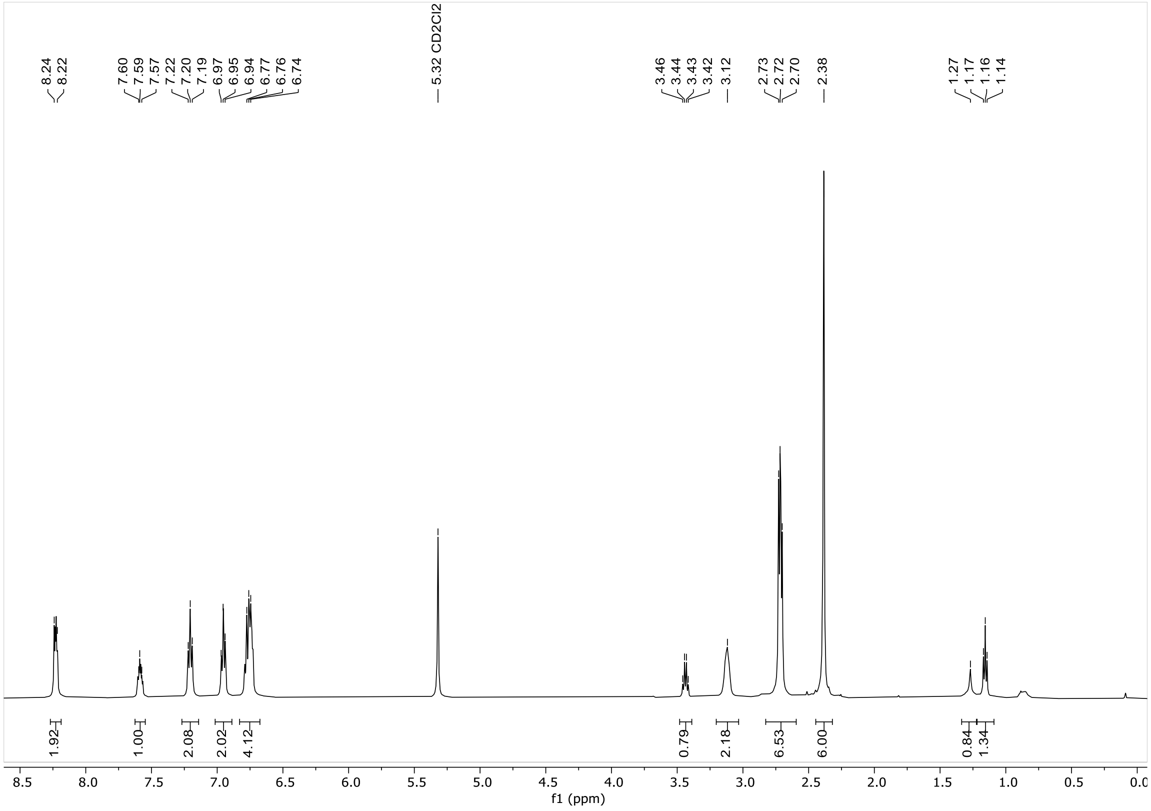

Figure S31. ^{1}H NMR spectrum of Fe(^{MeNH}PDI)$(CO)_2$ (**12**) in CD_2Cl_2.

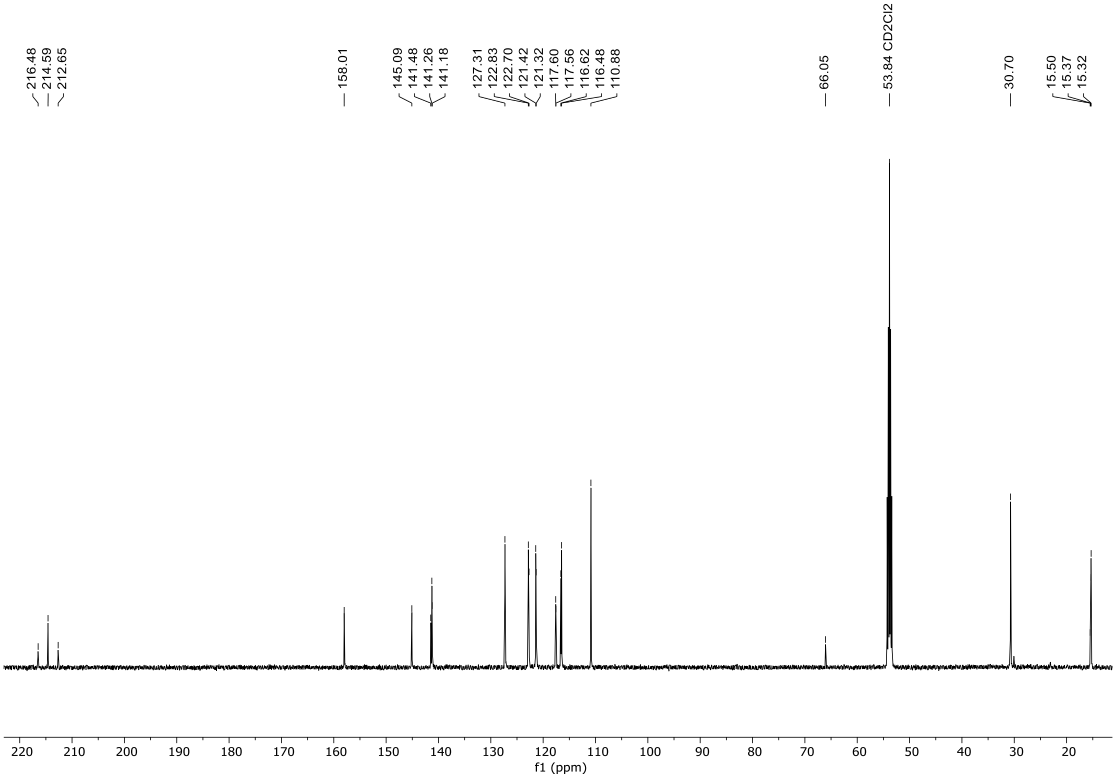

Figure S32. ^{13}C NMR spectrum of Fe(^{MeNH}PDI)$(CO)_2$ (**12**) in CD_2Cl_2.

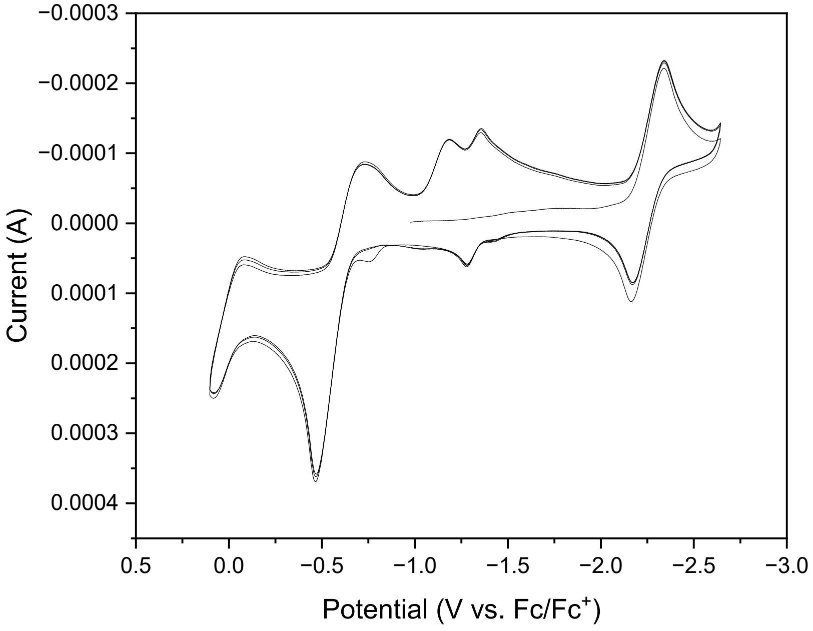

Figure S33. Cyclic voltammogram of Fe(^{MeNH}PDI)(CO)$_2$ (**12**). 1 mM in in MeCN; glassy carbon WE, Pt wire CE, and Ag/AgNO$_3$ in MeCN RE, 100 mM TBAPF$_6$ electrolyte.

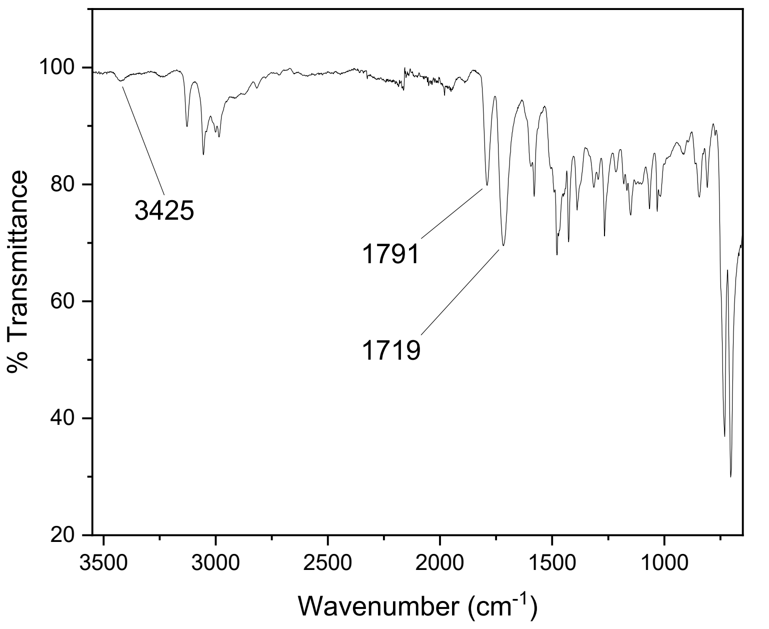

Figure S34. ATR FTIR spectrum of [Fe(^{MeNH}PDI)(NO)$_2$][BPh$_4$] (**13**).

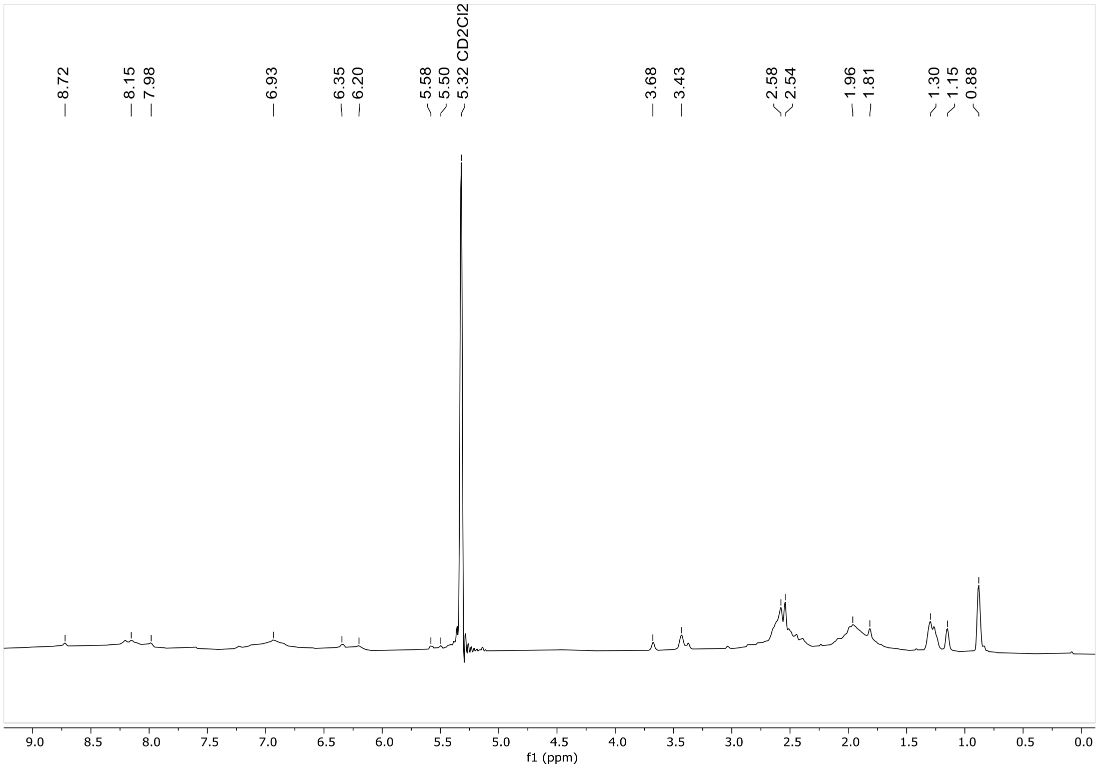

Figure S35. ^{1}H NMR spectrum of [Fe(^{MeNH}PDI)(NO)$_2$][BPh$_4$] (**13**) in CD_2Cl_2.

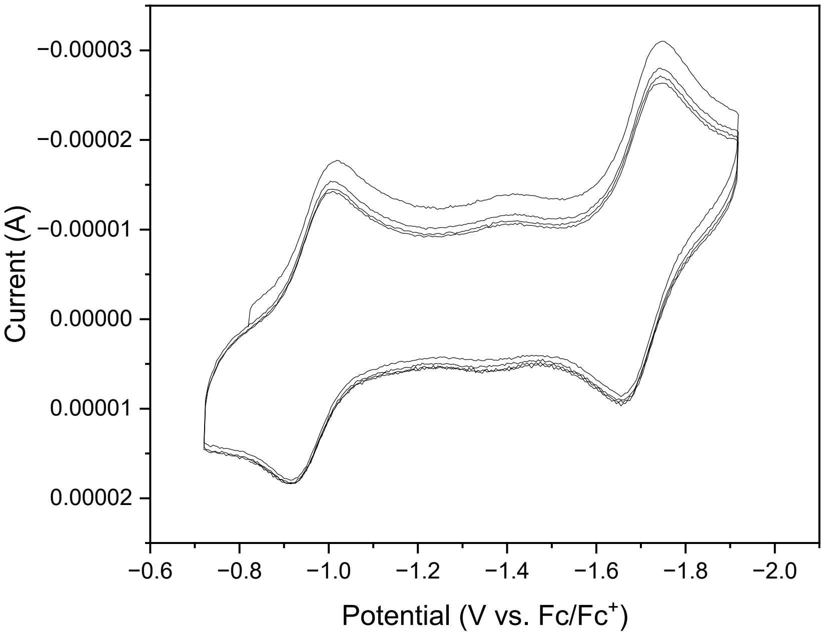

Figure S36. Cyclic voltammogram of [Fe(^{MeNH}PDI)(NO)$_2$][BPh$_4$] (**13**). 1 mM in in MeCN; glassy carbon WE, Pt wire CE, and Ag/$AgNO_3$ in MeCN RE, 100 mM $TBAPF_6$ electrolyte.

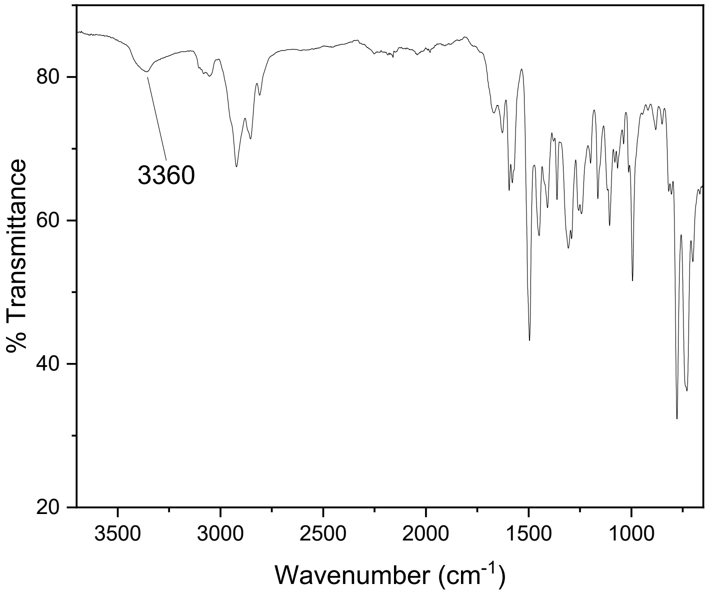

Figure S37. ATR FT-IR of blue product from the N-N coupling reaction of Fe(^{MeNH}PDI)$(NO)_2$ (**13**).

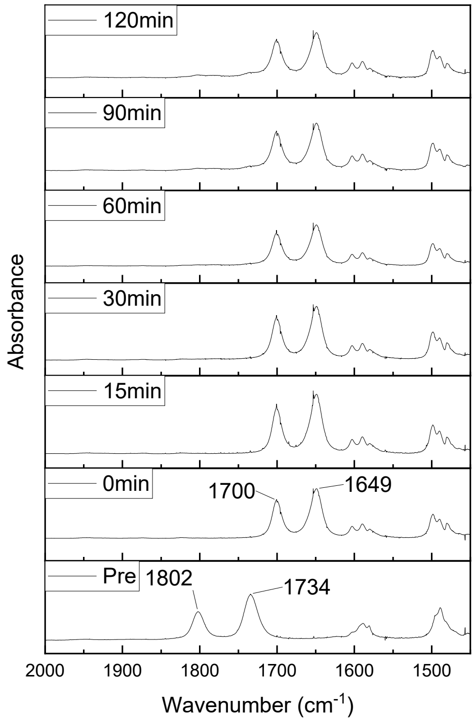

Figure S38. Solution phase FT-IR analysis of [Fe(^{PhNMe}PDI)$(NO)_2$][BPh_4] (**7**) reduced with Cobaltocene.

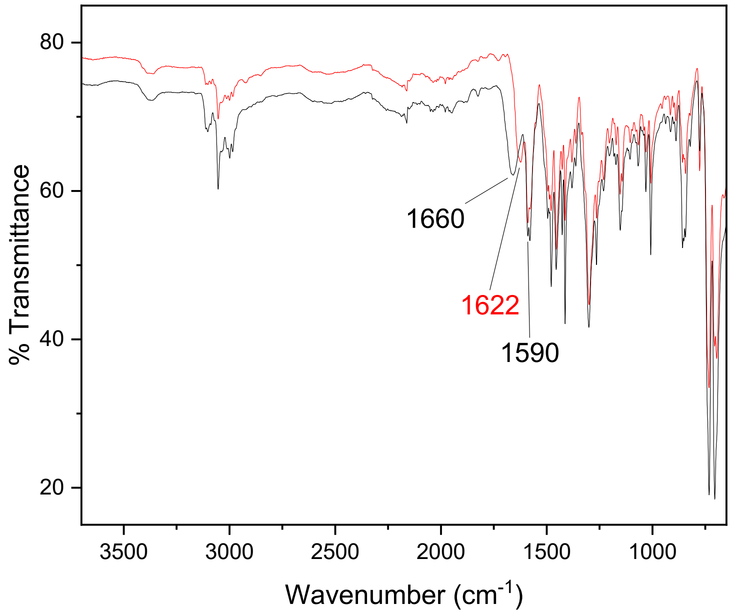

Figure S39. Overlayed ATR FT-IR spectra of crude reaction mixture from N-N coupling reaction of $[Fe(^{PhNH}PDI)(NO)_2][BPh_4]$ (**3**) (black curve) and $[Fe(^{PhNH}PDI)(^{15}NO)_2][BPh_4]$ (**3-^{15}N**) (red curve).

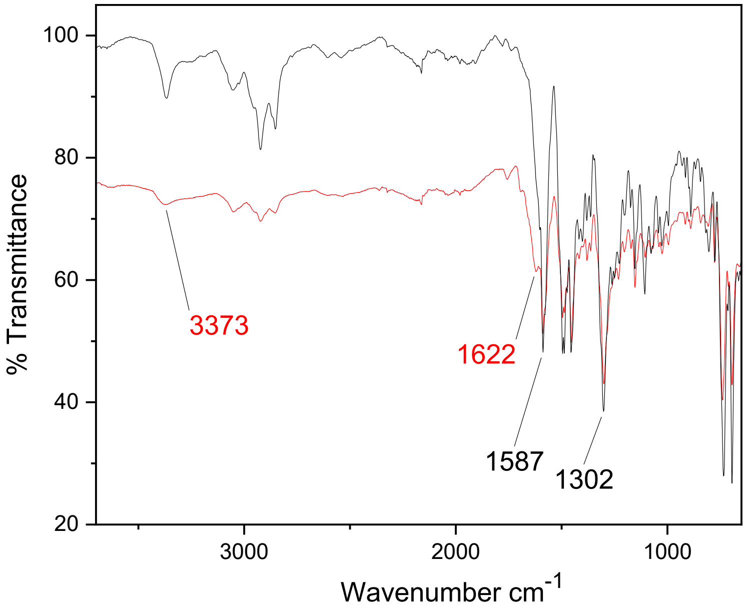

Figure S40. Overlayed ATR FT-IR spectra of $[Fe(^{PhNH}PDI)(NO)_2][BPh_4]$ (**3**) (black curve) and $[Fe(^{PhNH}PDI)(^{15}NO)_2][BPh_4]$ (**3-^{15}N**) (red curve) reduction with Cobaltocene.

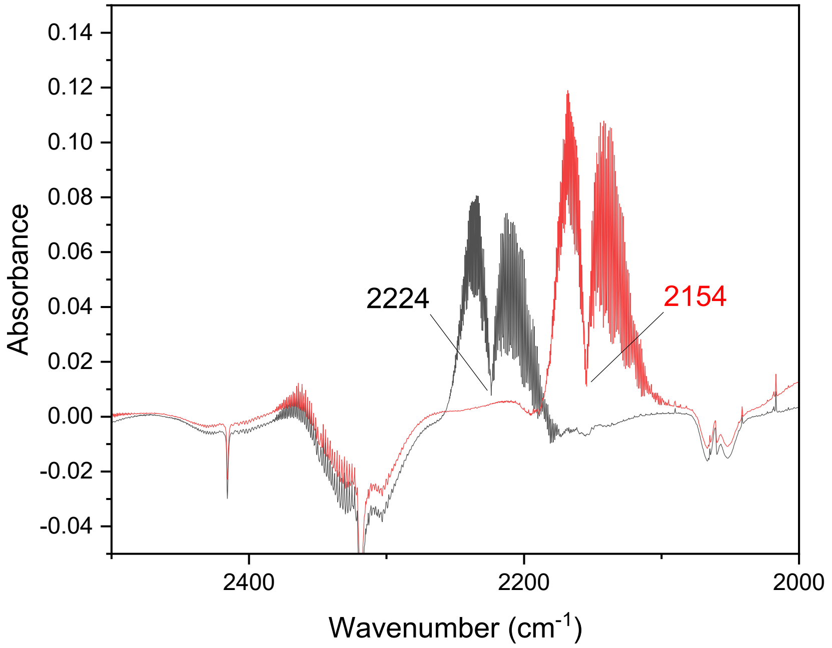

Figure S41. Overlayed headspace FT-IR spectra of [Fe(^{PhNH}PDI)(NO)$_2$][BPh$_4$] (**3**) (black curve) and [Fe(^{PhNH}PDI)(^{15}NO)$_2$][BPh$_4$] (**3-^{15}N**) (red curve) reduction with Cobaltocene.

[Fe(^{PhNH}PDI)(NO)$_2$][BPh$_4$] (3) and [Fe(^{PhNH}PDI)(^{15}NO)$_2$][BPh$_4$] (3-^{15}N) Mixed Isotope Reduction with Cobaltocene. The procedure follows the same method for *in-situ* formation of Fe(^{PhNH}PDI)(NO)$_2$ (**4**) but instead of only adding [Fe(^{PhNH}PDI)(NO)$_2$][BPh$_4$] (**3**), both [Fe(^{PhNH}PDI)(^{15}NO)$_2$][BPh$_4$] (**3-^{15}N**) (0.0250 g, 0.0268 mmol) and (**3**) (0.0250 g, 0.0269 mmol) were utilized. The solution was removed *in vacuo* after confirming the decomposition of the nitrosyl peaks via solution phase FT-IR. This was confirmed via color change from red to blue and $^{15}N_2O$ in the headspace. The blue solid was filtered through celite with about 15 mL of Et_2O. Slow evaporation yielded blue solid that displays ν_{NH}. FT-IR: ν_{N2O} = 2224 (formed by (**3**)), 2202 (formed by mixed isotope), 2180 (formed by mixed isotope), and 2155 cm^{-1} (formed by (**3-^{15}N**)).

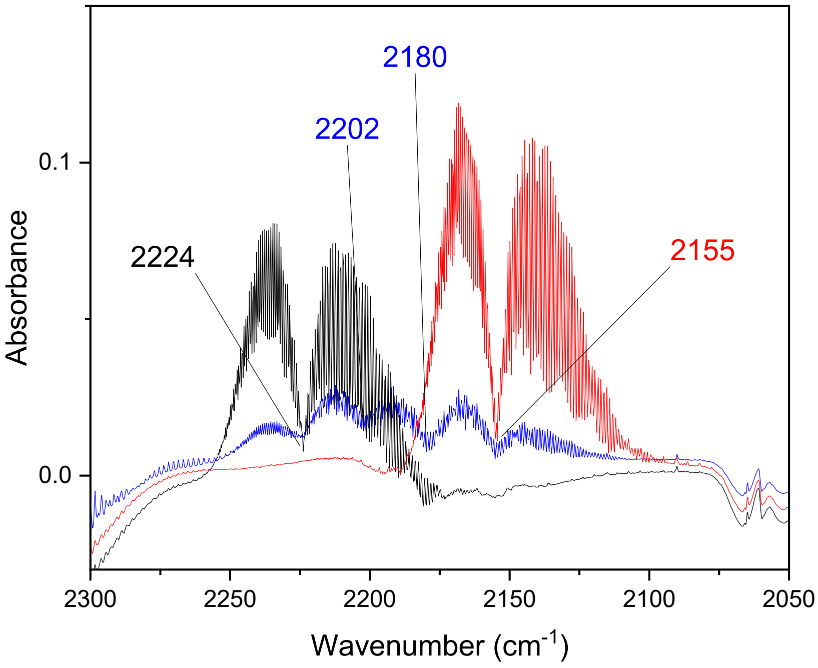

Figure S42. Overlayed headspace FT-IR spectra of [Fe(^{PhNH}PDI)(NO)$_2$][BPh$_4$] (**3**) (black curve), [Fe(^{PhNH}PDI)(^{15}NO)$_2$][BPh$_4$] (**3-^{15}N**) (red curve), and [Fe(^{PhNH}PDI)(NO)$_2$][BPh$_4$] (**3**) & [Fe(^{PhNH}PDI)(^{15}NO)$_2$][BPh$_4$] (3-^{15}N) Mixed Isotope (blue curve) reduction with Cobaltocene.

Reaction of Fe(^{PhNMe}PDI)(NO)$_2$ (8) with excess Ph$_2$NH. In a nitrogen-filled glovebox, (**8**) (0.0260 g, 0.0271 mmol) was added to a J-young NMR tube and dissolved in CD_2Cl_2. After collecting its ^{1}H NMR spectrum, the tube was brought back into the glovebox and an excess amount of Ph$_2$NH (0.0184 g, 0.109 mmol) was added. Subsequently, ^{1}H NMR spectra were collected at 0 minutes, 30 minutes, and 12 hours into the reaction. As **Figure S43** shows, headspace analysis was collected after 12 hours and an insignificant amount of N_2O was detected. **Figure S44** supports this by the absence of a H_2 resonance in the ^{1}H NMR spectrum.

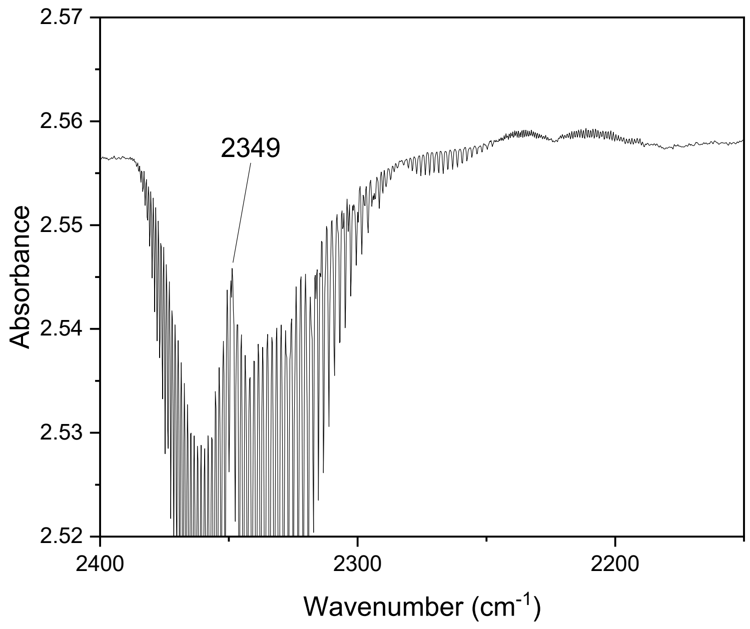

Figure S43. Headspace FT-IR of the reaction of Fe(^{PhNMe}PDI)(NO)$_2$ (**8**) with the addition of excess Ph$_2$NH. Spectrum was collected 12 hours after the reaction began. Inverted peak at 2349 cm^{-1} indicates presence of CO_2 in the background that was not present in the reaction headspace.

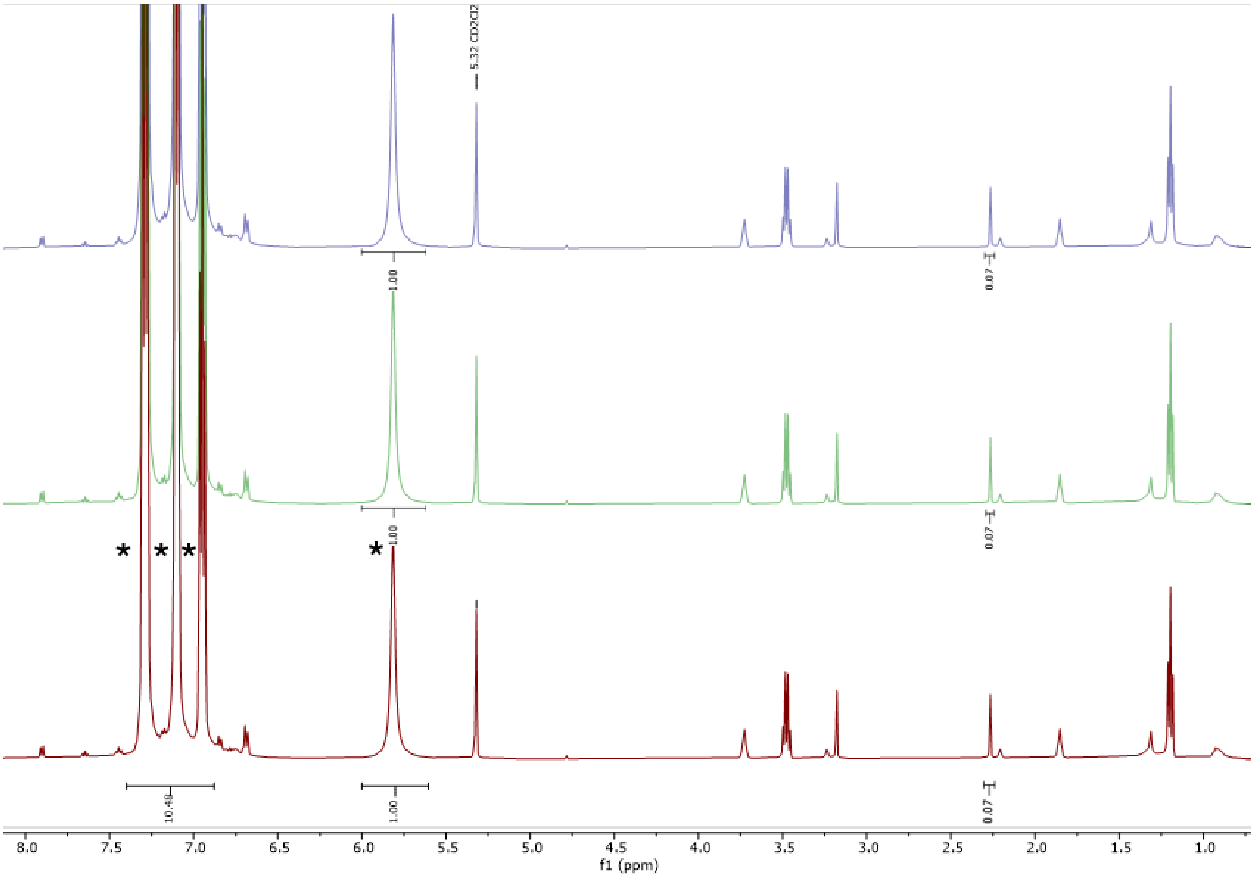

Figure S44. ^{1}H NMR spectra of the reaction of Fe(^{PhNMe}PDI)(NO)$_2$ **(8)** with excess Ph_2NH. From the bottom, a spectrum was collected when Ph_2NH was added (red), 30 minutes (green), and 12 hours (blue) into the reaction. Ph_2NH peaks marked with *.

Scheme 1: Reaction of Fe(^{PhNMe}PDI)(NO)$_2$ (**8**) with excess Ph_2NH.

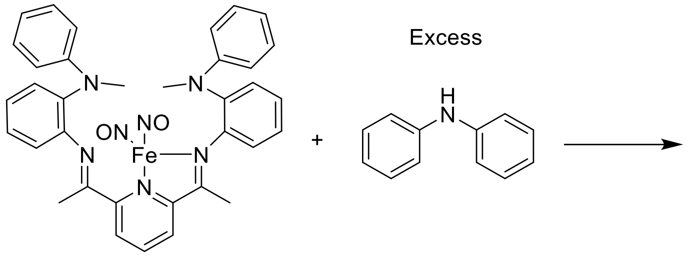

Reaction of Fe(^{PhNMe}PDI)(NO)$_2$ (8) with excess 9,10-Dihydroanthracene (DHA). In a J-Young NMR tube, **8** (0.0100 g, 0.0104 mmol) was dissolved in CD_2Cl_2. After collecting an ^{1}H NMR spectrum, an excess amount of DHA (0.00752 g, 0.0417 mmol) was added. Subsequently, ^{1}H NMR spectra were collected at 0 minutes, 60 minutes, and 12 hours into the reaction. Due to the consistent DHA resonances, **Figure S45** indicates that the nitrosyl groups of **8** stayed intact, meaning that N_2O was not formed. Additionally, the resonance for H_2 was not present.

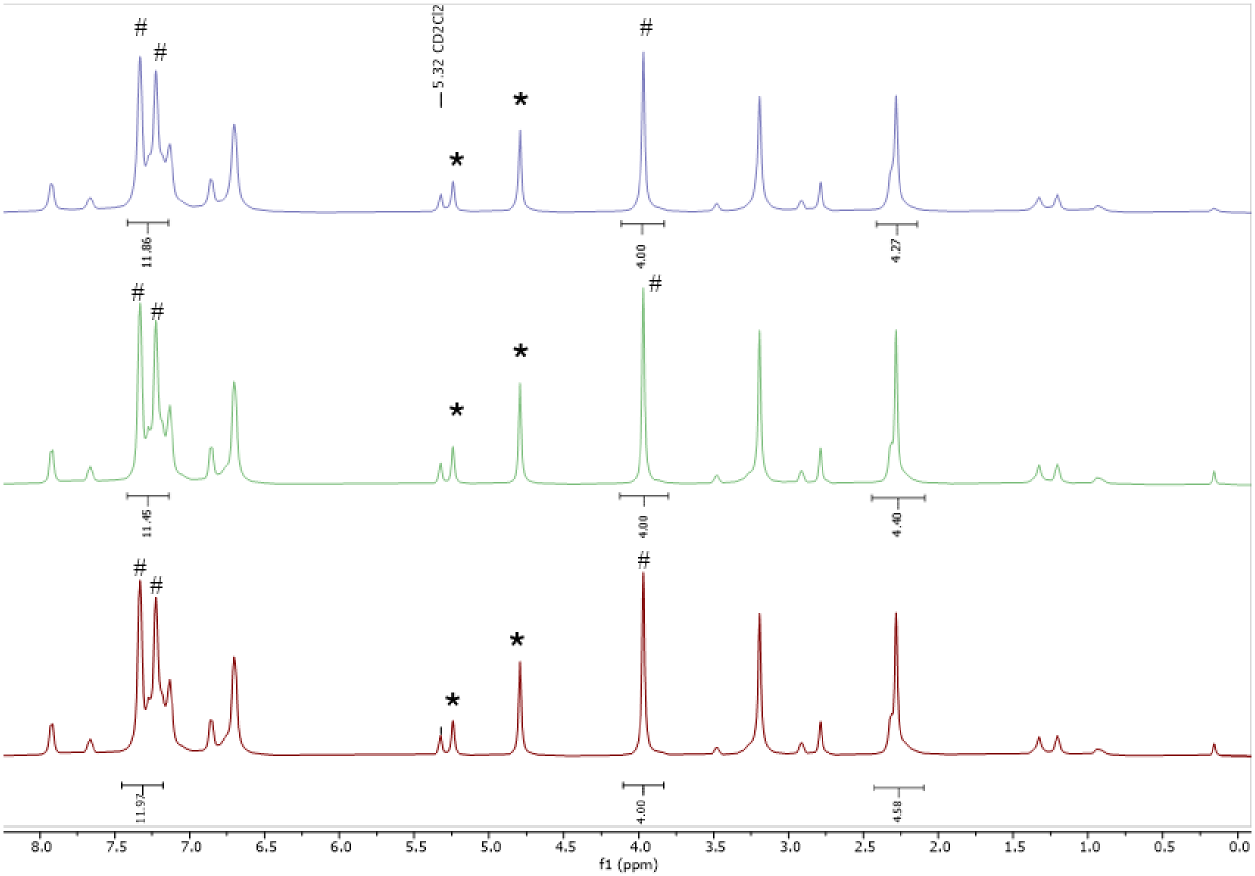

Figure S45. ^{1}H NMR spectra of the reaction of Fe(^{PhNMe}PDI)(NO)$_2$ (**8**) with excess DHA. From bottom to top a spectrum when DHA was added, 0 minutes (red), 60 minutes (green), and 12 hours (blue) into the experiment. DHA peaks marked with # and impurities marked with *.

Scheme 2: Reaction of Fe(^{PhNMe}PDI)(NO)$_2$ (**8**) with excess DHA.

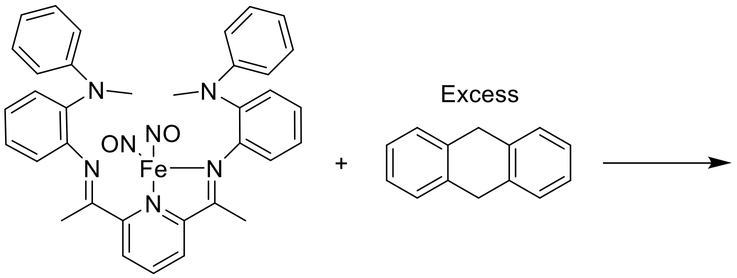

Protonation of Fe(didpa)(NO)2 (9) with [HNEt3][BPh4] acid. In a 20 mL scintillation vial, Fe(didpa)(NO)$_2$ (**9**) (0.0500 g, 0.0566 mmol) was dissolved in five mL of CH_2Cl_2 and was capped with a size 33 Suba-seal septum. In another scintillation vial, two equivalents of [HNEt3][BPh4] acid (0.0477 g, 0.113 mmol) was dissolved in five mL of CH_2Cl_2 and injected into the vial with Fe(didpa)(NO)$_2$ (**9**) and stirred overnight. The reaction was monitored via solution phase FT-IR and headspace was collected 12 hours into the reaction and analyzed via gas phase FT-IR. After headspace was collected, the solution was removed *in vacuo* and filtered through celite yielding **[Fe(didpa)(NO)$_2$]$^+$** (**10$^+$**). As **Figure S46** indicates, Fe(didpa)(NO)$_2$ (**9**) was oxidized to **[Fe(didpa)(NO)$_2$]$^+$** (**10$^+$**) and was not protonated. Additionally, N_2O was not detected in the gas phase FT-IR.

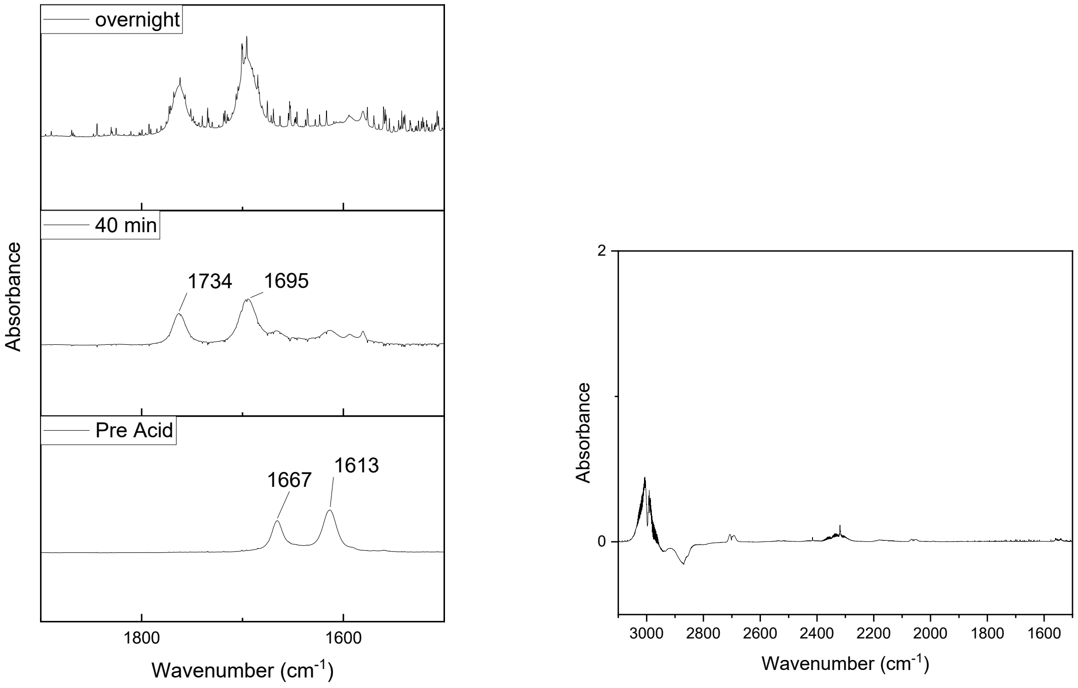

Figure S46. Solution phase FT-IR of protonation of (**9**) with [HNEt$_3$][BPh$_4$] acid (left). Headspace analysis via gas phase FT-IR collected after 12 hours into the reaction (right). Peaks at 1667 and 1613 cm^{-1} represent the nitrosyl groups of (**9**) and as shown in the figure, the peaks shift to 1734 and 1695 cm^{-1} in result of oxidation.

Scheme 3: Protonation of Fe(didpa)(NO)$_2$ (**9**) with [HNEt$_3$][BPh$_4$].

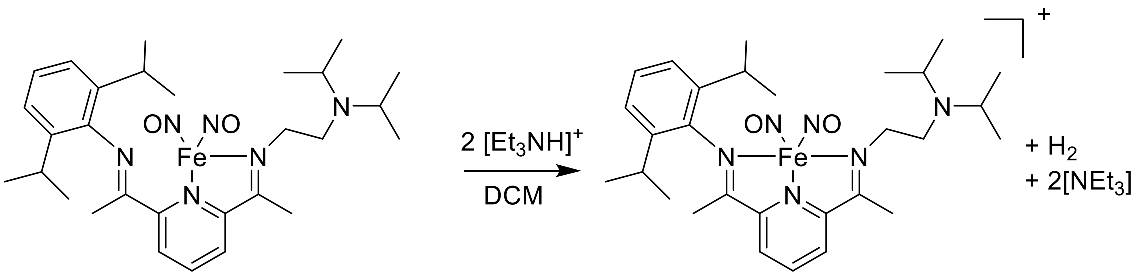

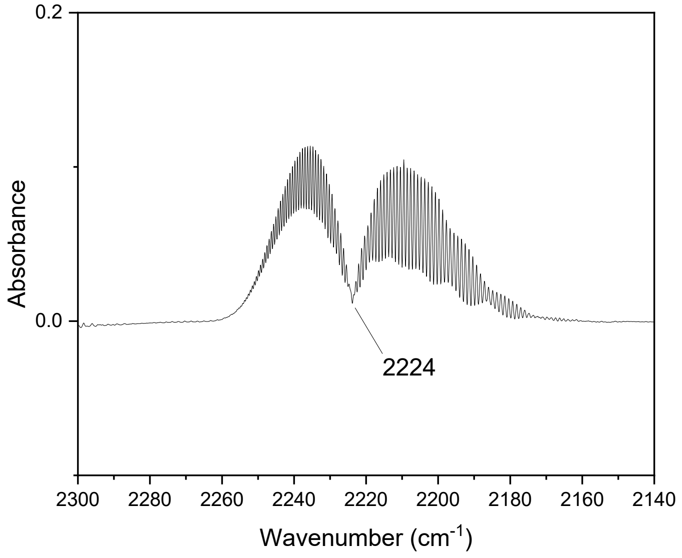

Figure S47. Gas phase FT-IR headspace analysis of [Fe(^{MeNH}PDI)(NO)$_2$][BPh$_4$] (**13**) reduced with cobaltocene.

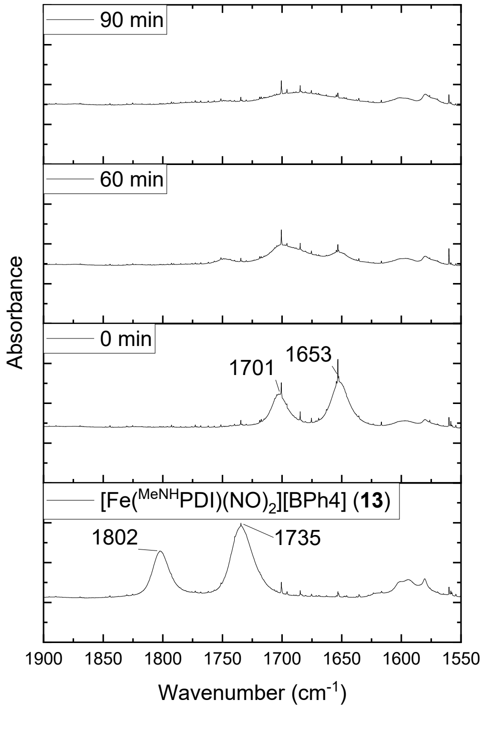

Figure S48. Solution phase FT-IR analysis of [Fe(^{MeNH}PDI)(NO)$_2$][BPh$_4$] (**13**) reduced with cobaltocene.

Reduction of [Fe(^{PhND}PDI)(NO)$_2$][BPh$_4$] (3ND) with cobaltocene. The deuterated analogue, [Fe(^{PhND}PDI)(NO)$_2$][BPh$_4$] (**3ND**) was prepared by stirring [Fe(^{PhNH}PDI)(NO)$_2$][BPh$_4$] (**3**) (0.1 g, 0.107mmol) in 10 mL of deuterated acetonitrile and one mL of D_2O for 48 hours. The mixture was dried via vacuum. Once dried, the solid was rinsed with ether to assure the removal of residual D_2O and dried overnight at 40 °C. This is confirmed by **Figure S49** showing the removal of the NH peak and the formation of the ND peak. FT-IR: ν_{ND} = 2497 cm^{-1}. $\nu_{N=O}$ = 1791, 1720 cm^{-1}

The reduction of [Fe(^{PhND}PDI)(NO)$_2$][BPh$_4$] (**3ND**) (0.05 g, 0.0536 mmol) follows the same method for the *in-situ* formation of Fe(^{PhNH}PDI)(NO)$_2$ (**4**). The reaction was monitored via solution phase FT-IR. The solvent was removed *in vacuo* after confirming the decomposition of the nitrosyl peaks via solution phase FT-IR. This was also confirmed via color change from red to blue. Following the reduction of [Fe(^{PhND}PDI)(NO)$_2$][BPh$_4$] (**3ND**), [Fe(^{PhNH}PDI)(NO)$_2$][BPh$_4$] (**3**) (0.0500 g, 0.0537 mmol) was then reduced with cobaltocene in the same manner within the next hour to assure consistent environmental variables. As **Figure S50** shows, the reaction rate of [Fe(^{PhND}PDI)(NO)$_2$][BPh$_4$] (**3ND**) did not change in comparison to [Fe(^{PhNH}PDI)(NO)$_2$][BPh$_4$] (**3**), indicating that N-N coupling is not likely facilitated by hydrogen atom transfer.

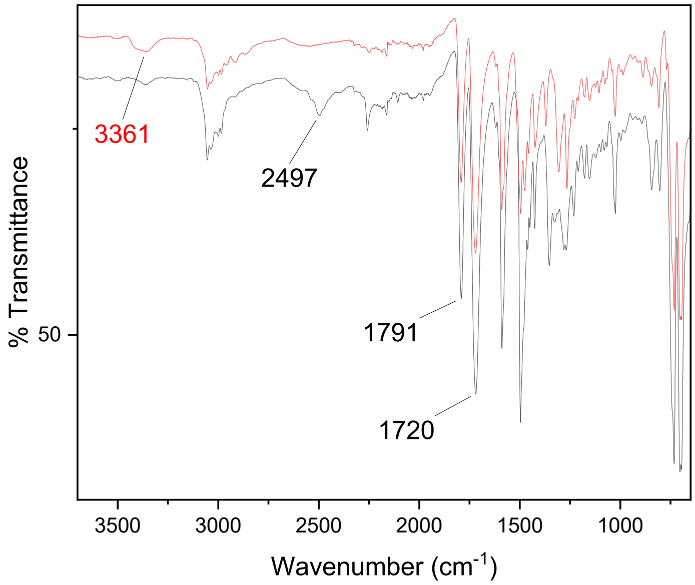

Figure S49. Overlaid ATR FT-IR of [Fe(^{PhNH}PDI)(NO)$_2$][BPh$_4$] (**3**) and [Fe(^{PhND}PDI)(NO)$_2$][BPh$_4$] (**3ND**)

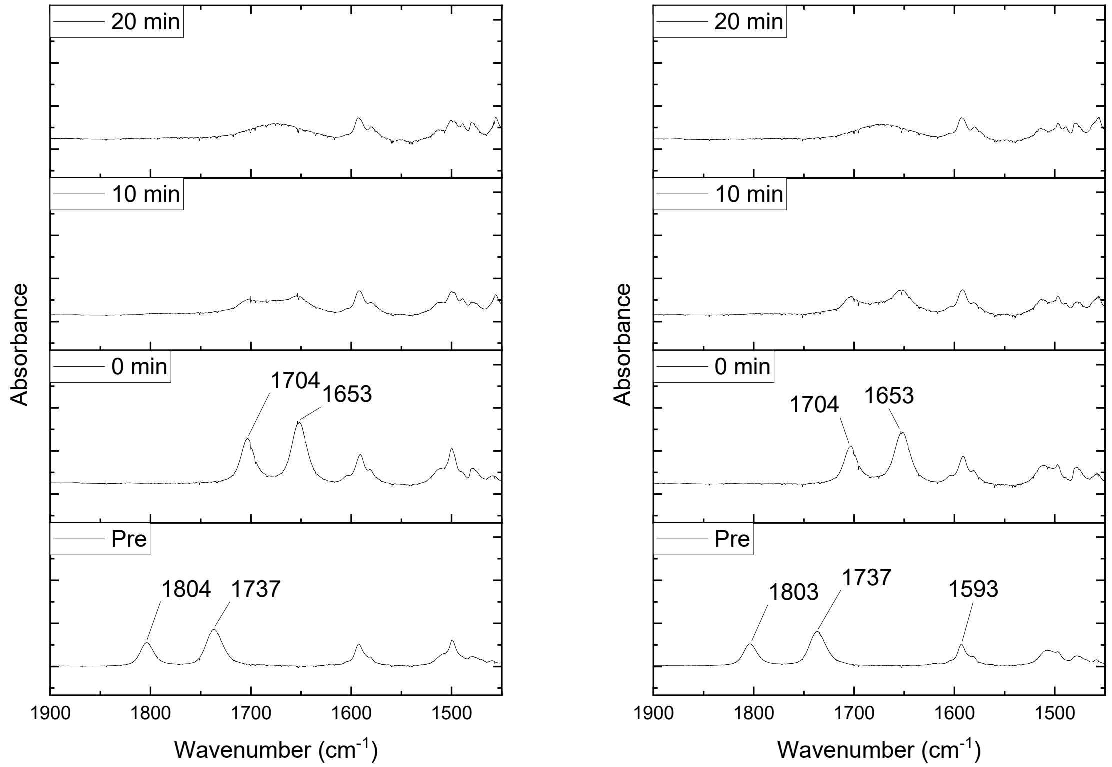

Figure S50. Solution phase FT-IR analysis of [Fe(^{PhND}PDI)(NO)$_2$][BPh$_4$] (**3ND**) (left) and [Fe(^{PhNH}PDI)(NO)$_2$][BPh$_4$] (**3**) (right) reduced with cobaltocene.

Reaction of [Fe(^{PhNMe}PDI)(NO)$_2$][BPh$_4$] (8) with MAP. To ensure that [Fe(^{PhNH}PDI)(NO)$_2$][BPh$_4$] (**3**) alone will not react with the MAP ligand, an NMR time study was completed using [Fe(^{PhNMe}PDI)(NO)$_2$][BPh$_4$] (**8**). In a J-Young NMR tube, [Fe(^{PhNMe}PDI)(NO)$_2$][BPh$_4$] (**8**) (0.00660 g, 0.0103 mmol) was dissolved in CD_2Cl_2. After collecting the ^{1}H NMR spectrum to ensure purity, two molar equivalents of the MAP ligand (0.0058 g, 0.0206 mmol) were added. Subsequently, ^{1}H NMR spectra were collected at 0 and 60 minutes into the reaction. An additional two molar equivalents of the MAP ligand (0.0058 g, 0.0206 mmol) were added after 60 minutes, resulting in a four molar equivalent excess. 12 hours into the reaction another ^{1}H NMR spectrum was taken. **Figure S50** does not show any new resonances, suggesting the lack of reactivity between [Fe(^{PhNMe}PDI)(NO)$_2$][BPh$_4$] (**8**) and MAP ligand. This is further supported by **Table S1**, comparing the integrations of resonance A and B (the resonances are marked in Table S1).

Reduction of [Fe(^{PhNH}PDI)(NO)$_2$][BPh$_4$] (3) and MAP with cobaltocene. In a 20 mL scintillation vial equipped with a stir bar and septa, [Fe(^{PhNH}PDI)(NO)$_2$][BPh$_4$] (**3**) (0.0500 g, 0.0537 mmol) and MAP ligand (0.0603 g, 0.2149 mmol) was dissolved in five mL of CH_2Cl_2. In another vial, cobaltocene (0.0102 g, 0.0537 mmol) was dissolved in five mL of CH_2Cl_2. A solution phase FT-IR of the [Fe(^{PhNH}PDI)(NO)$_2$][BPh$_4$] (**3**) and MAP ligand mixture were collected. Both vials were left in a -35 °C freezer for 30 minutes. The vials were taken out of the freezer and the cobaltocene solution was immediately syringed into the [Fe(^{PhNH}PDI)(NO)$_2$][BPh$_4$] (**3**) and MAP ligand solution dropwise while stirring vigorously, producing a bright red solution. After 12 hours, the headspace was collected and

analyzed via gas phase FT-IR. A solution phase FT-IR spectrum of the reaction mixture was also collected. The reaction was run at room temperature, 22-24 °C. As **Figure S52** shows, $[Fe(^{PhNH}PDI)(NO)_2][BPh_4]$ (**3**) reduced in the presence of the MAP ligand does not form N_2O, suggesting that the iron nitrosyl unit is labile, preventing the facilitation of N-N coupling. **Figure S53** shows that $[Fe(^{PhNH}PDI)(NO)_2][BPh_4]$ (**3**) was reduced to $[Fe(^{PhNH}PDI)(NO)_2]$ (**4**) but the nitrosyl bands stay intact, supporting **Figure S52** results.

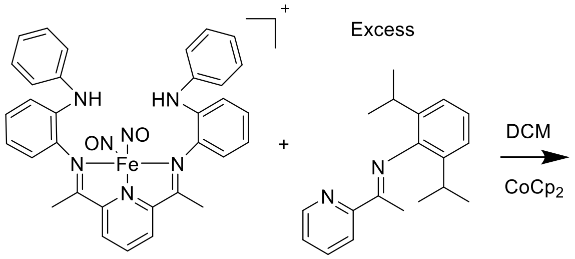

Scheme 4. Reduction of $[Fe(^{PhNH}PDI)(NO)_2][BPh_4]$ (**3**) and MAP with cobaltocene.

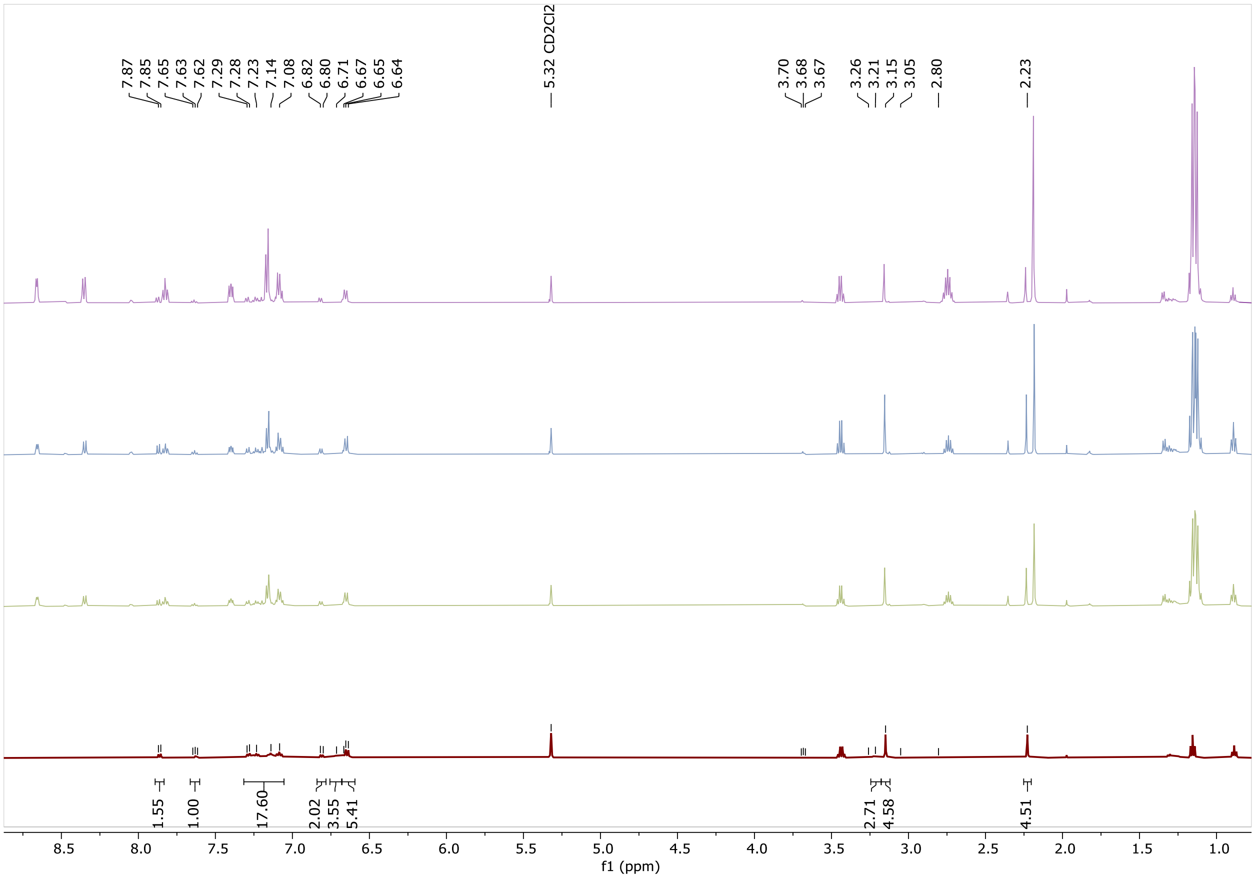

Figure S51. Staked ^1H NMR spectra of $Fe(^{PhNMe}PDI)(NO)_2$ (**8**) with MAP ligand. From the bottom is the ^1H NMR spectrum of, $Fe(^{PhNMe}PDI)(NO)_2$ (**8**) alone, 0 minutes into the reaction with two molar equivalents of MAP ligand, 60 minutes into the reaction, 12 hours into the reaction with four molar equivalents of MAP ligand.

Table S1. ^{1}H NMR time study integral table of reaction from **Scheme 4.**

	**Resonance A (2.262-2.211 ppm)** 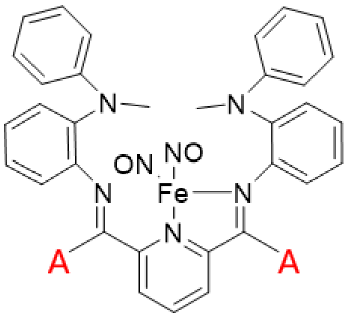	**Resonance B (2.211-2.154 ppm)** 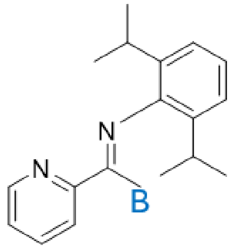
**Pre MAP Ligand**	4.51	N/A
**0 min**	5.22	10.32
**60 min**	5.55	10.92
**Excess overnight**	5.44	27.44

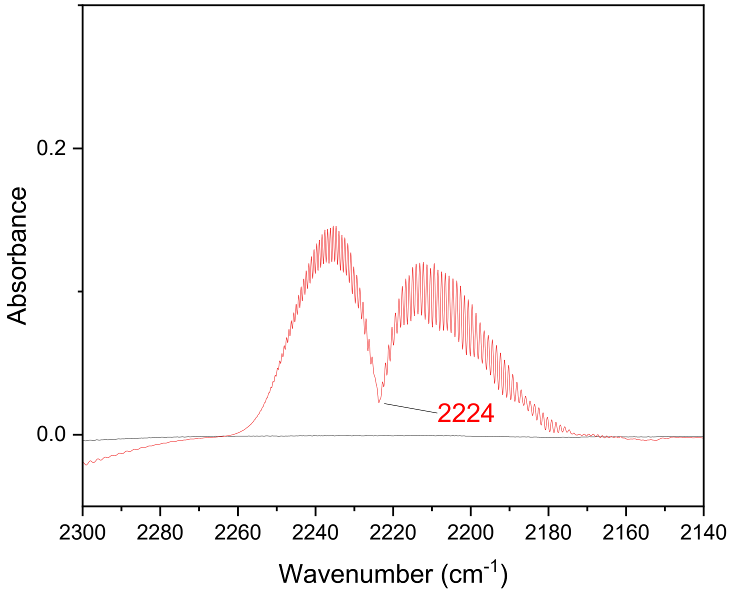

Figure S52. Overlaid gas phase FT-IR headspace analysis of [Fe(^{PhNH}PDI)(NO)$_2$][BPh$_4$] (**3**) with (black curve) and without MAP ligand (red curve) reduced with cobaltocene.

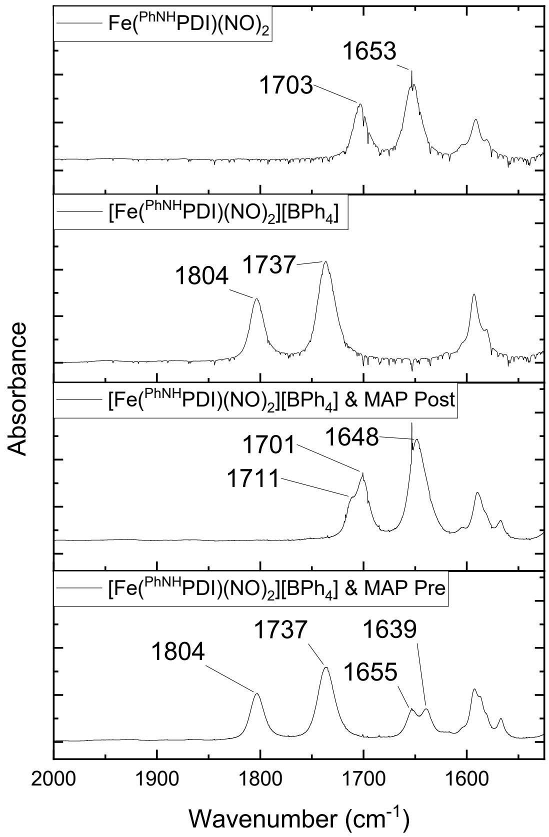

Figure S53. Solution phase FT-IR analysis of [Fe(^{PhNH}PDI)(NO)$_2$][BPh$_4$] (**3**) with MAP ligand reduced with cobaltocene. Solution phase FT-IR of [Fe(^{PhNH}PDI)(NO)$_2$][BPh$_4$] (**3**) and [Fe(^{PhNH}PDI)(NO)$_2$] (**4**).

Reduction of [Fe(^{PhNH}PDI)(NO)$_2$][BPh$_4$] (3) with CoTPP & Cobaltocene. Cobalt(II) meso-tetraphenylporphine (CoTPP) (0.0350 g, 0.0521 mmol) was weighed and dissolved in CH_2Cl_2 and pipetted into a shell vial equipped with a stir bar. A sample of the CoTPP solution was also collected for UV-Vis analysis. In a 20 mL scintillation vial equipped with a stir bar, [Fe(^{PhNH}PDI)(NO)$_2$][BPh$_4$] (**3**) (0.0500 g, 0.0537 mmol) was dissolved in five mL of CH_2Cl_2. In another vial cobaltocene (0.0102 g, 0.0537 mmol) was dissolved in five mL of CH_2Cl_2. The shell vial was carefully put into the vial containing [Fe(^{PhNH}PDI)(NO)$_2$][BPh$_4$] (**3**). Both vials were left in a -35 °C freezer for 30 minutes. The vials were taken out of the freezer and the cobaltocene solution was immediately added into the [Fe(^{PhNH}PDI)(NO)$_2$][BPh$_4$] (**3**) and CoTPP vial dropwise while stirring vigorously. The reaction was run at room temperature, 22-24 °C. After 12 hours of stirring, a sample of the CoTPP solution was collected for UV-Vis analysis. The contents of the shell vial and the reaction mixture were dried separately via vacuum. **Figure S54** does not display the CoTPP(NO) peak at 1693 cm^{-1}. Additionally, **Figure S55** does not contain the CoTPP(NO) peak at 538 nm.

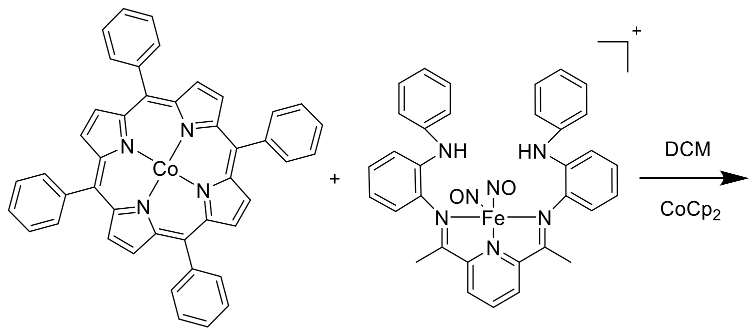

Scheme 5: Reduction of [Fe(^{PhNH}PDI)(NO)$_2$][BPh$_4$] (**3**) with CoTPP & cobaltocene

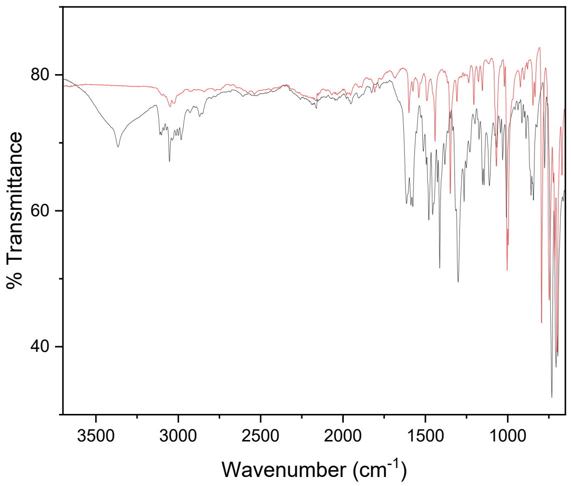

Figure S54. Overlayed ATR FT-IR of CoTPP solution (red curve) and crude [Fe(^{PhNH}PDI)(NO)$_2$][BPh$_4$] (**3**) reduced product (black curve) post reaction.

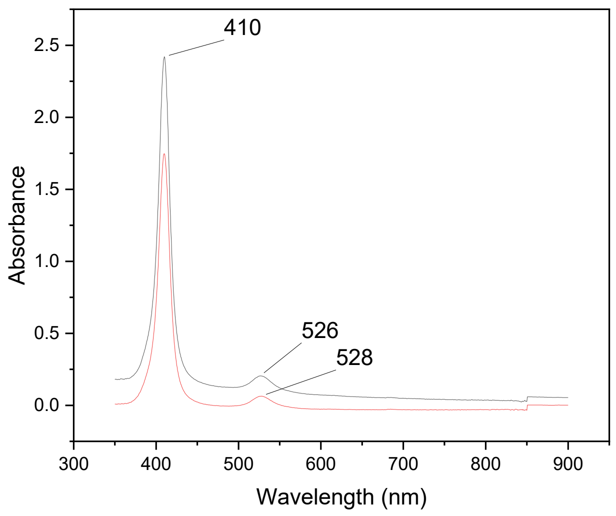

Figure S55. Overlayed UV-Vis of CoTPP before (black curve) and after (red curve) cobaltocene reduction with $[Fe(^{PhNH}PDI)(NO)_2][BPh_4]$ (**3**).

Synthesis of ^{BA}PDI Ligand. The ^{BA}PDI ligand was prepared from a literature adapted procedure.[3,4] A pressure tube containing excess aniline, 2,6-diacetylpyridine (0.400 g, 2.45 mmol), and Na_2SO_4 (4.00 g, 28.1 mmol) was sealed and placed in an oil bath at 100 °C for 12 hours. After which the pressure tube was left to cool to room temperature and filtered via vacuum to remove the Na_2SO_4. The solution collected was then layered in cold methanol and left to crystalize in the freezer at -35 °C overnight. The yellow crystals were collected and spectra matched literature values (0.404 g, 52.5% yield). FT-IR: $\nu_{C=N}$ = 1633 cm^{-1}, ^1H NMR (CD_2Cl_2) δ: 8.36, 8.34, 7.92, 7.90, 7.89, 7.41, 7.39, 7.37, 7.14, 7.13, 7.11, 6.85, 6.84, 2.40 ppm.

Synthesis of $Fe(^{BA}PDI)(NO)_2$ (15). In a 20 mL scintillation vial, [Na(18-crown-6)][$Fe(CO)_3(NO)$] (0.1621 g, 0.3545 mmol) was added and dissolved in five mL of CH_2Cl_2, giving a yellow solution. The vial containing [Na(18-crown-6)][$Fe(CO)_3(NO)$] was left in a -35 °C freezer for 30 minutes. In a 25 mL Schlenk flask equipped with a septa and stir bar, $NOBF_4$ (0.0414 g, 0.3545 mmol) was added. Once the $NOBF_4$ was added to the Schlenk flask the [Na(18-crown-6)][$Fe(CO)_3(NO)$] solution was immediately syringed into the Schlenk flask and static vacuum was applied. The yellow solution immediately turned reddish brown, and the solution was left to stir for 30 minutes. After 30 minutes to ensure the completion of the reaction, solution phase FT-IR was collected to confirm the presence of carbonyl and nitrosyl peaks of $Fe(CO)_2(NO)_2$. Once confirmed, the ^{BA}PDI (0.100 g, 0.319 mmol) dissolved in two mL of CH_2Cl_2 and left in the freezer at -35 °C for 30 minutes. This was then syringed into the flask containing $Fe(CO)_2(NO)_2$, turning the solution reddish purple. The flask was stirred for three hours wilst maintaining static vacuum. After three hours, solution phase FT-IR was used to confirm the presence of $Fe(^{BA}PDI)(NO)_2$ (**15**) and the lack of carbonyl and nitrosyl bands associated with $Fe(CO)_2(NO)_2$. The solution was filtered through an alumina plug to remove salts and concentrated via vacuum. Once, the solution was concentrated to three mL it was layered in pentane for crystallization. **Figure S58** confirms the stability of the $Fe(^{BA}PDI)(NO)_2$ (**15**). FT-IR: ν_{NO} = 1692 and 1632 cm^{-1}, $\nu_{C=N}$ =1592 cm^{-1}.

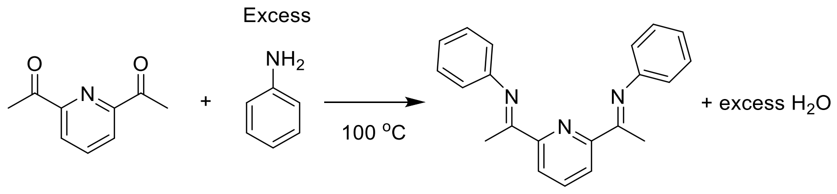

Scheme 6. Synthesis of ^{BA}PDI.

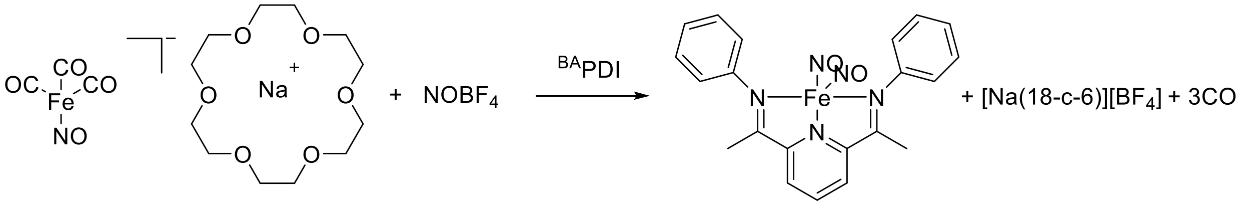

Scheme 7. Synthesis of Fe(^{BA}PDI)$(NO)_2$ (**15**).

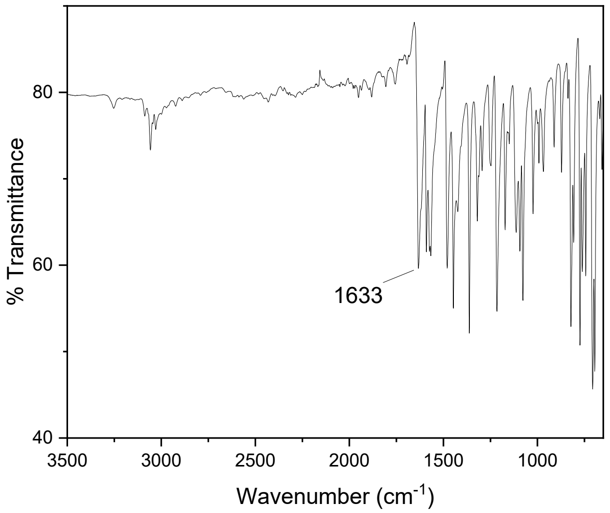

Figure S56. ATR FT-IR of ^{BA}PDI.

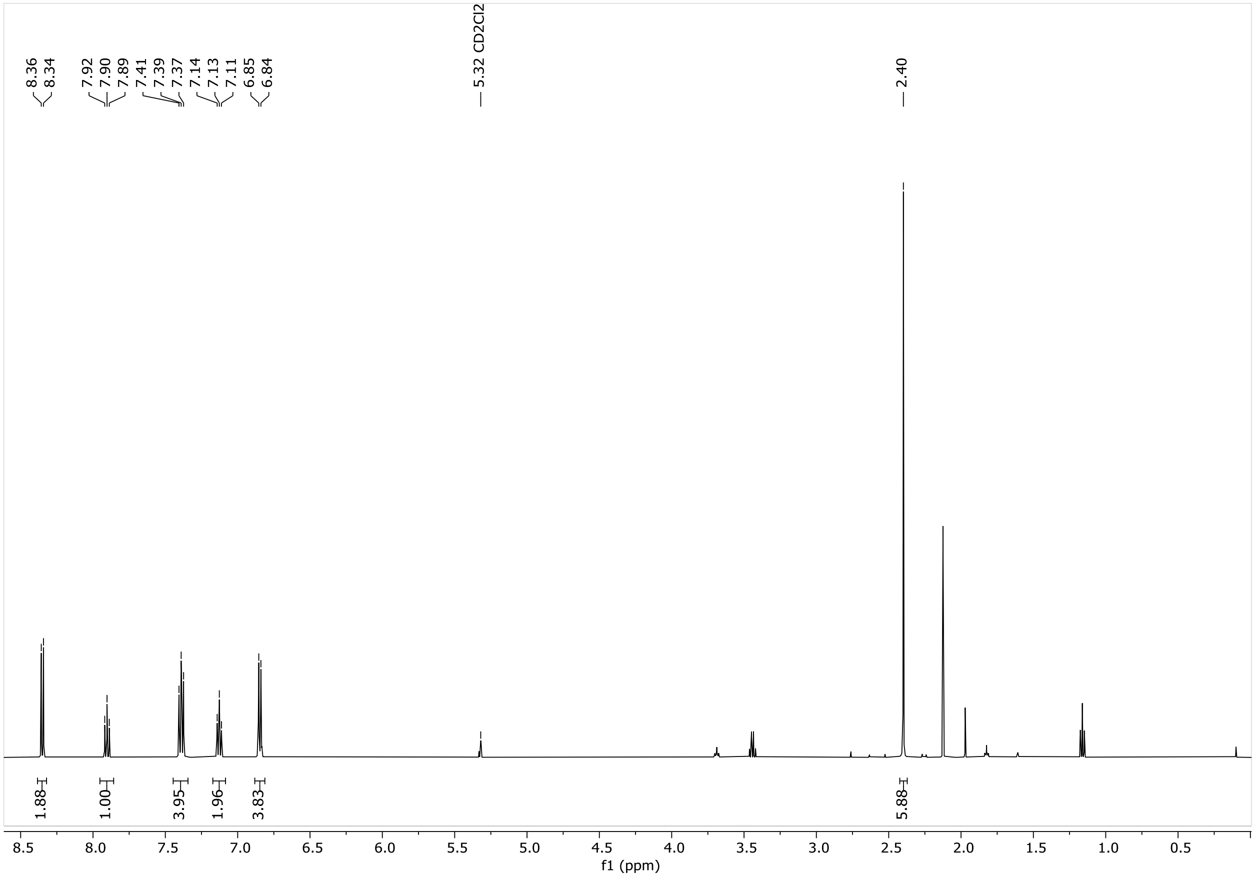

Figure S57. ^{1}H NMR of ^{BA}PDI.

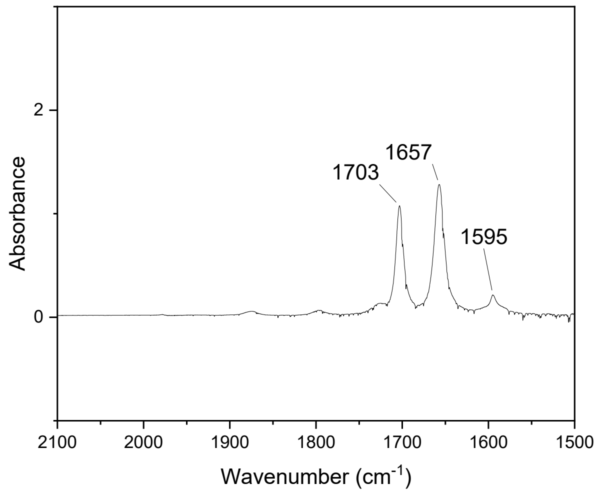

Figure S58. Solution phase FT-IR of Fe(^{BA}PDI)(NO)$_2$ (**15**).

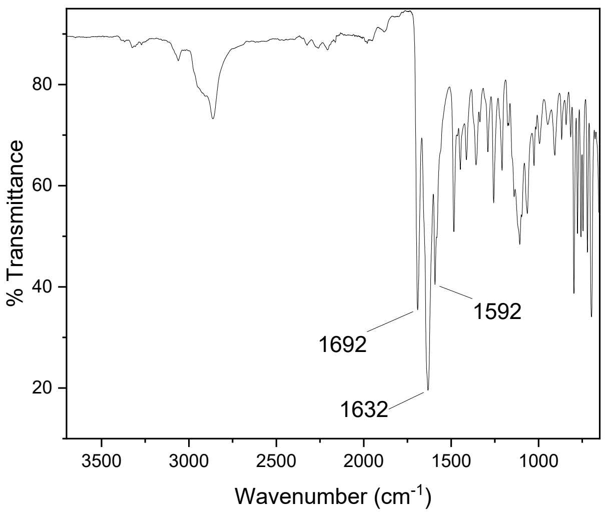

Figure S59. ATR FT-IR of Fe(^{BA}PDI)$(NO)_2$ (**15**).

Crystallography Experimental

X-ray diffraction data for Fe(^{PhNH}PDI)Cl_2 (**1**), Fe(^{PhNH}PDI)$(CO)_2$ (**2**), Fe(^{PhNMe}PDI)Cl_2 (**5**), and Fe(^{MeNH}PDI)Cl_2 (**9**) were collected at 100 K on a Bruker D8 VENTURE Duo Fixed Chi Three-Circle Diffractometer using MoK_α radiation (λ = 0.71073 Å). Data for **1** were integrated with SAINT V8.40B and corrected for absorption using SADABS 2016/2.[5,6] The structure was solved by dual methods with SHELXT and refined by full-matrix least-squares methods against F^2 using SHELXL.[7,8] All non-hydrogen atoms were refined with anisotropic displacement parameters. Hydrogen atoms bound to carbon atoms were placed in geometrically calculated positions and refined using a riding model. Locations of hydrogen atoms bound to nitrogen atoms were identified from difference maps, placed and refined. Many of the N—H bond distances after refinement were unreasonably short or long. All the N—H bond distances were treated with a bond distance restraint (DFIX 0.87 0.02). Isotropic thermal parameters of the placed hydrogen atoms were fixed to 1.2 times the U value of the atoms they are linked to (1.5 times for methyl groups). The structure has void spaces containing disordered solvent molecules. Suitable models were not possible, therefore the Olex2 solvent mask routine was used to account for this electron density and the model is refined against these data. A total of 737 electrons per unit cell were corrected for. Seven reflections with (Iobs-Icalc)/Sigma(W) > 10 were omitted from the refinement (-2 -2 2, -2 -1 4, -1 -1 4, -1 0 4, -1 0 5, 2 2 4, 2 4 1). Calculations and refinement of the structure was carried out using APEX4[9], and Olex2[10] software.

X-ray diffraction data for **2** have been corrected for absorption using SADABS[11] area detector absorption correction program. Using Olex2, the structure was solved with the SHELXT structure solution program using Direct Methods and refined with the SHELXL refinement package using least squares minimization. All non-hydrogen atoms were refined with anisotropic thermal paramo9eters. Most of the hydrogen atoms in the investigated structure were located from difference Fourier maps but finally their positions were placed in geometrically calculated positions and refined using a riding model. Isotropic thermal parameters of the placed hydrogen atoms were fixed to 1.2 times the U value of the atoms they are linked to (1.5 times for methyl groups). Hydrogen atoms connected to nitrogen atoms were located from the difference map, placed, and refined. Some of the phenyl groups displayed some disorder. Each individual ring disorder was modeled over two positions using a PART instruction and tied to a free variable. The disorder model incorporates bond similarity restraints, thermal ellipsoid constraints and restraints. One of the phenyl rings was constrained to be a hexagon due to unreasonable lengthening of bond lengths. PLATON ADDSYMM indicated possible missed symmetry (translation), however closer evaluation of the structure and data indicates it is pseudo symmetry. Calculations and refinement of structures were carried out using APEX4, SHELXTL,[12] and Olex2 software.

All data for **5** were integrated with SAINT V8.40B and corrected for absorption using SADABS 2016/2. The structure was solved by dual methods with SHELXT and refined by full-matrix least-squares methods against F^2 using SHELXL. All non-hydrogen atoms were refined with anisotropic displacement parameters. Hydrogen atoms of the investigated structure were located from difference Fourier maps but finally their positions were placed in geometrically calculated positions and refined using a riding model. Isotropic thermal parameters of the placed hydrogen atoms were fixed to 1.2 times the U value of the atoms they are linked to (1.5 times for methyl groups). Calculations and refinement of the structure was carried out using APEX4, and Olex2 software.

X-ray diffraction data for **9** have been corrected for absorption using SADABS[11] area detector absorption correction program. Using Olex2, the structure was solved with the SHELXT structure solution program using Direct Methods and refined with the SHELXL refinement package using least squares minimization. All non-hydrogen atoms were refined with anisotropic thermal parameters. Hydrogen atoms in the investigated structure were located from difference Fourier maps but finally their positions were placed in geometrically calculated positions and refined using a riding model. Isotropic thermal parameters of the placed hydrogen atoms were fixed to 1.2 times the U value of the atoms they are linked to (1.5 times for methyl groups). Hydrogen atoms connected to heteroatoms were located from the difference map, placed, and refined. Calculations and refinement of structures were carried out using APEX4, SHELXTL, and Olex2 software.

Crystallographic Data for Fe(^{PhNH}PDI)Cl_2 (**1**)**:**
$C_{33}H_{29}Cl_2FeN_5$ (M =622.36, g/mol), triclinic, a = 20.3060(5) Å, b = 22.5265(6) Å, c = 25.0178(6) Å, α =77.7180(10)°, β = 85.5890(10)°, γ = 66.3770(10)°, V = 10244.3(5) Å^3, Z = 10, T = 100 K, μ(MoKα) = 0.521mm^{-1}, ρ_{calc} = 1.009g/cm^3, 253065 reflections measured, 29479 unique (R_{int} = 0.0861, R_{sigma} = 0.0496), R_1 = 0.0456 (I > 2σ(I)), wR_2 = 0.1212 (all data).

Crystallographic Data for Fe(^{PhNH}PDI)$(CO)_2$ (**2**):
$C_{35}H_{29}FeN_5O_2$ (M =607.48 g/mol): triclinic, space group P-1 (no. 2), a = 17.1658(9) Å, b = 18.7691(9) Å, c = 22.0019(12) Å, α = 71.425(2)°, β = 69.776(2)°, γ = 65.741(2)°, V = 5933.8(5) Å^3, Z = 8, T = 100 K, μ(MoKα) = 0.549 mm-1, Dcalc = 1.360 g/cm^3, 2Θmax = 52.786°, 282852 reflections measured, 24100 unique (R_{int} = 0.0802, R_{sigma} = 0.0560), R_1 = 0.0706 (I > 2σ(I), wR_2 = 0.1465 (all data).

Crystallographic Data for Fe(^{PhNMe}PDI)Cl_2 (**5**)**:**
$C_{35}H_{33}Cl_2FeN_5$ (M =650.41, g/mol), triclinic, a = 9.9918(5) Å, b = 13.2928(6) Å, c = 13.3163(6) Å, α =64.342(2)°, β = 80.848(2)°, γ = 82.876(2)°, V = 1570.94(13) Å^3, Z = 2, T = 100 K, μ(MoKα) = 0.683mm^{-1}, ρ_{calc} = 1.375g/cm^3, 85084 reflections measured, 9619 unique (R_{int} = 0.0478, R_{sigma} = 0.0276), R_1 = 0.0410 (I > 2σ(I)) and wR_2 was 0.0977 (all data).

Crystallographic Data for Fe(^{MeNH}PDI)Cl_2 (**9**):
$C_{23}H_{25}Cl_2FeN_5$ (M =498.23 g/mol): monoclinic, space group $P2_1/c$ (no. 14), a = 18.8969(7) Å, b = 15.7547(5) Å, c = 15.8217(6) Å, β = 90.457(2)°, V = 4710.2(3) Å^3, Z = 8, T = 100 K, μ(MoKα) = 0.887 mm^{-1}, $Dcalc$ = 1.405 g/cm^3, 2Θmax =52.838°, 225274 reflections measured, 9650 unique (R_{int} = 0.0967, R_{sigma} = 0.0364), R_1 = 0.0374 (I > 2σ(I)), wR_2 = 0.0826 (all data).

Computational Experimental

Computational Details

The geometry of the four-coordinate Fe(^{PhNH}PDI)$(NO)_2$ (**4***), Fe(^{PhNMe}PDI)$(NO)_2$ (**8***), and Fe(^{MeNH}PDI)NO_2 (**14***) complexes were optimized, via closed-shell Kohn-Sham density functional theory (KS-DFT) at the BLYP/def2-TZVP and PBE-D3/def2-TZVP levels of theory.[13-16] The initial guess structure for Fe(^{PhNH}PDI)$(NO)_2$ (**4***) was generated using the PDI ligand scaffold on the crystallography data for Fe(didpa)$(NO)_2$ (**9**) and adjusting the ligand arm accordingly. Fe(^{PhNMe}PDI)$(NO)_2$ and Fe(^{MeNH}PDI)NO_2 structures were constructed via modification of the optimized structure for Fe(^{PhNH}PDI)$(NO)_2$. Local minima were verified by confirming the absence of imaginary frequencies in harmonic vibrational analysis.

Hydrogen bond interaction energetics for Fe(^{PhNH}PDI)$(NO)_2$ (**4***) and Fe(^{MeNH}PDI)NO_2 (**14***) were assessed through PBE-D3/def2-TZVP relaxed coordinate scans along the shorter of the two hydrogen-bonded N-H distances, starting from the minimum-energy configuration and assessed at regular intervals to beyond 5 Angstroms. All other degrees of freedom are unconstrained during these scans. All geometry optimizations and coordinate scans used the ORCA 4.0.1.2 program package.[17]

Optimized geometries of Fe(^{PhNH}PDI)$(NO)_2$ (**4***), Fe(^{PhNMe}PDI)$(NO)_2$ (**8***), and Fe(^{MeNH}PDI)NO_2 (**14***).

Table S2. Optimized geometric data: comparison of GGA functionals with and without D3 dispersion correction.

	Fe(^{PhNH}PDI)(NO)$_2$ (**4***)		Fe(^{PhNMe}PDI)(NO)$_2$ (**8***)		Fe(^{MeNH}PDI)NO$_2$ (**14***)
	BLYP	PBE-D3	BLYP	PBE-D3	PBE-D3
Bond	Length (Å)	Length (Å)	Length (Å)	Length (Å)	Length (Å)
Fe—(NO)$_\alpha$	1.673	1.656	1.672	1.635	1.656
Fe—(NO)$_\beta$	1.653	1.636	1.651	1.657	1.636
N$_\alpha$—O$_\alpha$	1.194	1.186	1.191	1.184	1.185
N$_A$—N(O)	3.451	3.247	4.787	4.800	3.236
N$_\beta$—O$_\beta$	1.192	1.184	1.191	1.184	1.186
(O)N---H$_A$	2.578	2.367	-	-	2.432
O---H$_A$	3.231	3.044	-	-	3.111
(O)N---H$_B$	3.717	3.422	-	-	2.874
O---H$_B$	3.531	3.257	-	-	2.630
Fe—N$_{pyridyl}$	2.046	1.989	2.062	2.003	1.979
Fe—N$_{imine}$	2.008	1.974	1.999	1.953	1.983
Angle	Angle (°)	Angle (°)	Angle (°)	Angle (°)	Angle (°)
Fe-N$_\alpha$-O$_\alpha$	162.8	163.5	160.8	176.7	163.9
Fe-N$_\beta$-O$_\beta$	177.7	178.0	176.1	161.2	176.5
N$_A$-H---N$_\alpha$(O)	143.7	143.8	-	-	135.2
N$_B$-H---N$_\beta$(O)	143.6	145.0	-	-	131.7

Table S2 Key. The arm labeled α is the arm with greater H-bonding character according to bond length data.

Summary: The optimized N-H---N bond angle for the α hydrogen bond at the PBE-D3/def2-TZVP level is 143.8°, in line with moderate H-bonding.

Hydrogen bond dissociation energy, including dispersion effects

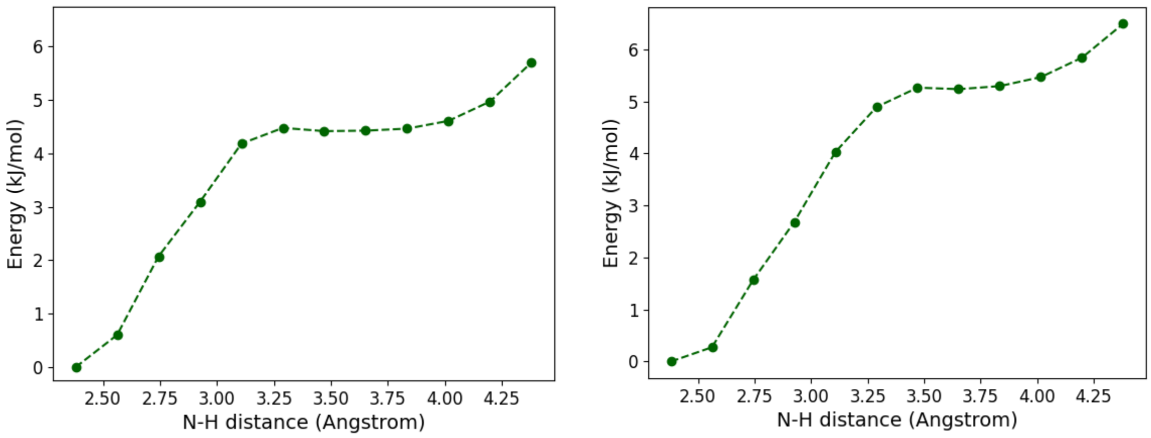

Figure S60. Potential energy curve along the shorter nitrosyl N – amine H hydrogen bond distance in Fe(^{PhNH}PDI)(NO)$_2$ (**4***) (left) and Fe(^{MeNH}PDI)NO$_2$ (**14***) (right), via relaxed scans at the PBE-D3/TZVP level.

72
BIS32, BLYP/def2-TZVP optimized geometry

Fe	-0.142025	0.078666	-0.042796
N	1.903241	0.098635	-0.091674
C	2.721898	1.185745	-0.075712
C	2.199215	2.507118	-0.573026
N	2.323983	3.490274	0.255879
C	2.001011	4.835328	-0.006251
C	2.349498	5.526192	-1.182256
C	2.092755	6.891224	-1.324596
H	2.392884	7.410220	-2.231704
C	1.439895	7.577082	-0.292590
H	1.200417	8.632482	-0.405065
C	1.085985	6.921584	0.887518
C	1.380775	5.554478	1.070797
N	1.037109	4.828649	2.205823
C	0.714472	5.252044	3.511364
C	1.124798	6.483657	4.061689
C	0.786489	6.812485	5.378567
C	0.053350	5.927700	6.176477
C	-0.337741	4.693724	5.639460
C	-0.019685	4.359207	4.323376
H	-0.344534	3.407804	3.906452
H	-0.903671	3.988364	6.245174
H	-0.204783	6.191455	7.199170
H	1.114414	7.767511	5.784573
H	1.731126	7.168690	3.478510
H	1.171287	3.827618	2.095652
H	0.559483	7.464772	1.664676
H	2.869665	4.993178	-1.972742
C	1.673126	2.548530	-1.995018
H	0.923503	3.335935	-2.122233
H	1.237163	1.586839	-2.272439
H	2.498679	2.750454	-2.693008
C	4.038571	1.101941	0.396147
H	4.642165	2.003452	0.420314
C	4.544614	-0.127396	0.832481
H	5.561542	-0.202150	1.209504
C	3.728053	-1.252495	0.774957
H	4.094008	-2.218192	1.109126
C	2.408295	-1.123496	0.305541
C	1.463267	-2.237136	0.223278
N	0.228086	-1.894442	-0.091705
C	-0.819195	-2.870101	-0.219275
C	-1.352993	-3.483408	0.922193
C	-2.399260	-4.403288	0.821638
C	-2.926317	-4.698948	-0.440610
C	-2.421301	-4.080618	-1.585078
H	-2.868375	-4.291412	-2.550891
C	-1.361811	-3.152784	-1.505748
N	-0.836187	-2.479991	-2.616453
C	-1.095529	-2.709945	-3.983982
C	-1.223523	-1.602224	-4.847680
C	-1.407222	-1.788370	-6.219389
C	-1.483680	-3.078246	-6.758706
C	-1.355919	-4.180925	-5.905523

C	-1.155112	-4.007128	-4.533753
H	-1.019895	-4.873753	-3.893074
H	-1.399168	-5.190228	-6.310912
H	-1.639076	-3.222361	-7.825378
H	-1.507496	-0.918711	-6.865689
H	-1.196184	-0.596525	-4.431530
H	-0.482724	-1.551673	-2.398137
H	-3.752745	-5.399935	-0.537804
H	-2.802115	-4.870438	1.716355
H	-0.945550	-3.216505	1.894775
C	1.929224	-3.652777	0.466335
H	1.172249	-4.373395	0.152595
H	2.138360	-3.825512	1.531561
H	2.856574	-3.852677	-0.083264
N	-0.598928	0.623227	1.449258
O	-0.961392	1.043030	2.504776
N	-1.086137	0.691674	-1.280269
O	-1.910833	1.301300	-1.890744

72
BIS32, PBE-D3/TZVP optimized geometry

H	0.221621	0.228129	-0.038571
N	2.546332	0.472930	-0.410011
Fe	2.153211	2.065197	-0.182365
N	1.934714	2.858239	1.628746
C	2.886612	2.956572	2.583837
C	4.016233	1.980858	2.549695
N	5.192871	2.504291	2.493169
C	6.377085	1.760577	2.525703
C	6.651841	0.752917	3.462007
C	7.897033	0.128958	3.502783
H	8.102865	-0.633576	4.254350
C	8.869923	0.484076	2.563698
H	9.836718	-0.021819	2.560115
C	8.623102	1.480013	1.622141
C	7.389050	2.154247	1.599365
N	7.053981	3.126748	0.677374
C	7.881678	3.944582	-0.098251
C	9.210533	4.247395	0.247524
C	9.968710	5.092123	-0.562797
C	9.423940	5.660595	-1.715867
C	8.095418	5.379090	-2.048092
C	7.331521	4.527840	-1.255374
H	6.300141	4.295170	-1.528512
H	7.648657	5.818929	-2.941478
H	10.023980	6.319691	-2.344167
H	10.997609	5.318202	-0.276578
H	9.638361	3.852761	1.167915
H	6.064674	3.367871	0.679964
H	9.380073	1.722152	0.878442
H	5.889663	0.497549	4.198421
C	3.661348	0.522028	2.639121
H	4.416820	-0.102015	2.144045
H	2.678446	0.338905	2.190510
H	3.606404	0.212912	3.695724
C	2.836740	3.947404	3.565554

H	3.648586	4.006108	4.288964
C	1.763747	4.843775	3.586624
H	1.717106	5.631143	4.339586
C	0.759246	4.717968	2.635582
H	-0.085466	5.405609	2.620269
C	0.859455	3.711237	1.663864
C	-0.113734	3.487564	0.609139
N	0.252018	2.587007	-0.282215
C	-0.587583	2.228605	-1.368463
C	-0.909763	3.161941	-2.357941
C	-1.703683	2.806618	-3.445999
C	-2.166623	1.491829	-3.546946
C	-1.840635	0.545400	-2.579787
H	-2.173602	-0.485011	-2.693971
C	-1.046497	0.887108	-1.469701
N	-0.663556	-0.011797	-0.487960
C	-1.105062	-1.323478	-0.291262
C	-0.180137	-2.288127	0.147720
C	-0.593580	-3.590236	0.418202
C	-1.929157	-3.962379	0.245012
C	-2.850675	-3.005313	-0.186603
C	-2.452328	-1.695266	-0.447336
H	-3.189455	-0.949136	-0.743118
H	-3.900830	-3.275925	-0.310503
H	-2.249016	-4.984839	0.448007
H	0.140803	-4.324496	0.753612
H	0.870603	-2.010287	0.255501
H	-2.774457	1.189865	-4.401132
H	-1.945031	3.543684	-4.211812
H	-0.506137	4.171950	-2.268923
C	-1.421752	4.215350	0.601686
H	-2.139990	3.715071	-0.057176
H	-1.302965	5.250564	0.242688
H	-1.838911	4.266198	1.616845
N	3.108071	3.095059	-1.020731
O	3.821526	3.808558	-1.640727
O	3.083740	-0.528463	-0.747603

78

BIS32C, BLYP/def2-TZVP optimized geometry

Fe	-0.607141	0.518657	0.068900
N	1.342671	0.702227	-0.577525
C	2.131944	1.809192	-0.635095
C	1.508820	3.172545	-0.792257
N	1.976638	4.080801	-0.006743
C	1.665003	5.452306	-0.076484
C	1.801088	6.200725	-1.267216
C	1.660202	7.589503	-1.265573
H	1.789833	8.146154	-2.192691
C	1.352418	8.260109	-0.074107
H	1.222359	9.341289	-0.068433
C	1.203987	7.534216	1.109969
C	1.366605	6.138169	1.134306
N	1.210983	5.401447	2.348794
C	1.929252	5.750159	3.505882
C	3.167956	6.434851	3.414649

C	3.898648	6.747423	4.559675
C	3.436629	6.383837	5.833337
C	2.223603	5.696225	5.934062
C	1.473183	5.382871	4.795105
H	0.531935	4.855153	4.915473
H	1.844284	5.399880	6.911517
H	4.013918	6.626119	6.723480
H	4.848504	7.270695	4.453331
H	3.558577	6.708116	2.437789
C	0.041889	4.518354	2.463247
H	-0.308129	4.254137	1.463825
H	-0.791798	5.008814	2.995978
H	0.301164	3.592081	2.989333
H	0.962689	8.046182	2.039700
H	2.074686	5.687763	-2.186770
C	0.487705	3.354076	-1.899253
H	-0.160489	4.215331	-1.709092
H	-0.124213	2.456758	-2.014087
H	1.006623	3.525914	-2.855185
C	3.529566	1.717757	-0.563723
H	4.112552	2.633658	-0.587250
C	4.140029	0.464241	-0.451360
H	5.223648	0.381482	-0.385555
C	3.342632	-0.675178	-0.432379
H	3.792044	-1.660792	-0.351819
C	1.942672	-0.541747	-0.506318
C	1.025842	-1.681151	-0.514145
N	-0.257648	-1.366293	-0.498149
C	-1.296524	-2.337853	-0.657127
C	-2.159421	-2.576659	0.428063
C	-3.217843	-3.478849	0.320681
C	-3.452529	-4.132721	-0.895537
C	-2.624418	-3.877781	-1.989722
H	-2.815348	-4.366407	-2.943181
C	-1.531546	-2.995488	-1.896527
N	-0.710253	-2.728914	-3.042775
C	-0.202660	-3.780355	-3.835560
C	0.153214	-3.573977	-5.190052
C	0.717598	-4.606108	-5.949409
C	0.936850	-5.871040	-5.397858
C	0.580010	-6.088789	-4.059442
C	0.023767	-5.069008	-3.288090
H	-0.241358	-5.269199	-2.253837
H	0.740341	-7.066158	-3.604979
H	1.370421	-6.671333	-5.994250
H	0.977955	-4.410897	-6.989134
H	-0.013805	-2.611191	-5.663645
C	-0.857031	-1.392220	-3.643718
H	-1.163106	-0.684750	-2.870654
H	0.097413	-1.052192	-4.064387
H	-1.616825	-1.384203	-4.444655
H	-4.290114	-4.820790	-0.999476
H	-3.863159	-3.658740	1.178781
H	-1.976378	-2.044358	1.358723
C	1.559981	-3.091255	-0.496369
H	0.750842	-3.811722	-0.357994

H	2.277557	-3.222610	0.325572
H	2.079821	-3.338234	-1.432248
N	-0.611573	0.707699	1.708942
O	-0.694447	0.857878	2.887859
N	-1.923854	1.242974	-0.663246
O	-2.944819	1.840770	-0.804193

78
BIS32C, PBE-D3/def2-TZVP optimized geometry

Fe	0.029367	-0.044147	0.511877
N	1.998332	-0.104778	0.151907
C	2.867192	0.927258	0.048535
C	2.363435	2.247673	-0.431872
N	2.731914	3.268029	0.258764
C	2.457649	4.586760	-0.125906
C	2.900859	5.119484	-1.349891
C	2.773031	6.478946	-1.623808
H	3.144135	6.877877	-2.568853
C	2.171834	7.325957	-0.686985
H	2.055037	8.389199	-0.901266
C	1.706158	6.806546	0.518944
C	1.845524	5.444573	0.818990
N	1.365125	4.900983	2.034024
C	1.675974	5.492153	3.258306
C	2.824669	6.302962	3.399354
C	3.150894	6.863153	4.628802
C	2.360340	6.629694	5.759795
C	1.234961	5.816338	5.632744
C	0.888221	5.253214	4.403972
H	0.000541	4.626208	4.338741
H	0.605468	5.613900	6.501467
H	2.623067	7.069794	6.721895
H	4.047602	7.481108	4.707477
H	3.466547	6.474822	2.535843
C	0.281776	3.931172	1.966654
H	0.234863	3.508177	0.959954
H	-0.696678	4.398218	2.185111
H	0.446232	3.109512	2.677210
H	1.229151	7.455314	1.254879
H	3.399391	4.460587	-2.062358
C	1.555069	2.264759	-1.702113
H	0.872967	3.124425	-1.729165
H	0.983321	1.337475	-1.812755
H	2.232631	2.347723	-2.567164
C	4.217904	0.781901	0.369879
H	4.864114	1.655293	0.299479
C	4.699278	-0.464287	0.782478
H	5.748869	-0.589119	1.050615
C	3.825640	-1.541333	0.837904
H	4.169703	-2.527427	1.146599
C	2.474955	-1.346116	0.507097
C	1.476589	-2.397290	0.501495
N	0.245898	-1.970665	0.274627
C	-0.828146	-2.848600	0.008281
C	-1.964700	-2.795551	0.826814
C	-3.057934	-3.620016	0.579691

C	-3.039940	-4.485438	-0.518334
C	-1.934297	-4.511977	-1.364342
H	-1.926546	-5.158336	-2.243005
C	-0.812020	-3.707271	-1.118943
N	0.292337	-3.701238	-2.009276
C	0.853414	-4.891293	-2.479340
C	1.624692	-4.915242	-3.660084
C	2.225788	-6.098262	-4.093489
C	2.078104	-7.285157	-3.377340
C	1.316002	-7.268531	-2.204189
C	0.715392	-6.097691	-1.756368
H	0.133555	-6.108589	-0.835335
H	1.190269	-8.183061	-1.621472
H	2.546133	-8.206782	-3.723011
H	2.811329	-6.084559	-5.014537
H	1.751336	-4.011458	-4.253286
C	0.562649	-2.457292	-2.719345
H	0.128736	-1.621953	-2.159583
H	1.646400	-2.287321	-2.806508
H	0.123140	-2.462850	-3.732448
H	-3.900280	-5.120847	-0.733202
H	-3.928157	-3.577168	1.235768
H	-1.958393	-2.095548	1.663324
C	1.848621	-3.821955	0.753912
H	0.952942	-4.442886	0.866317
H	2.450472	-3.908236	1.671327
H	2.442433	-4.235544	-0.075500
N	-0.169435	0.353511	2.085558
O	-0.381728	0.637974	3.215144
N	-1.068433	0.692154	-0.487198
O	-1.962361	1.371614	-0.862565

58
BIS33, PBE-D3/def2-TZVP optimized geometry

Fe	0.066735	0.056500	0.078854
N	2.045113	0.017884	0.032494
C	2.862586	1.093750	0.015118
C	2.275770	2.387133	-0.455222
N	2.182825	3.311659	0.431951
C	1.684473	4.591178	0.143368
C	2.240558	5.443701	-0.813698
C	1.782506	6.755454	-0.964247
H	2.242373	7.414409	-1.701136
C	0.739946	7.208667	-0.154064
H	0.370698	8.230003	-0.260451
C	0.163200	6.374589	0.803845
C	0.617395	5.052458	0.972962
N	0.057615	4.176506	1.860184
H	0.537167	3.287958	1.966112
C	-0.936879	4.545673	2.834623
H	-0.589321	5.342548	3.518853
H	-1.188508	3.658554	3.426791
H	-1.861459	4.899146	2.347990
H	-0.648159	6.747771	1.428936
H	3.077321	5.081256	-1.414451

C	1.886566	2.463574	-1.906397
H	1.110582	3.223360	-2.065901
H	1.527746	1.487371	-2.254340
H	2.761509	2.734455	-2.519514
C	4.186622	1.012179	0.443039
H	4.796511	1.914585	0.441900
C	4.692056	-0.215832	0.884770
H	5.720113	-0.293401	1.239453
C	3.866393	-1.332514	0.871478
H	4.228367	-2.298909	1.219974
C	2.540646	-1.199106	0.430969
C	1.575550	-2.282105	0.367702
N	0.357940	-1.904130	0.025866
C	-0.710219	-2.828762	-0.091121
C	-1.142970	-3.572274	1.009934
C	-2.232011	-4.437367	0.910466
C	-2.892958	-4.555700	-0.314571
C	-2.482733	-3.814131	-1.420735
H	-3.020899	-3.909791	-2.363226
C	-1.387061	-2.932593	-1.339050
N	-0.933623	-2.209681	-2.419294
H	-0.367237	-1.405577	-2.151852
C	-1.756438	-2.020256	-3.596942
H	-1.253632	-1.309818	-4.264259
H	-1.880741	-2.966539	-4.145850
H	-2.760402	-1.623299	-3.358244
H	-3.751306	-5.222598	-0.410418
H	-2.566714	-4.999045	1.782314
H	-0.632134	-3.431289	1.964160
C	1.988397	-3.696316	0.633405
H	1.248871	-4.396378	0.229722
H	2.078772	-3.892685	1.713957
H	2.966899	-3.903222	0.178927
N	-0.342692	0.580202	1.573262
O	-0.627970	1.026842	2.634540
N	-0.815574	0.757785	-1.134740
O	-1.570759	1.434607	-1.748497

References:

1) Teprovich, J. A., P. K. Sudhadevi Antharjanam, Prasad, E., Pesciotta, E. N., & Flowers, R. A. (2008). Generation of Sm ^{II} Reductants Using High Intensity Ultrasound. *European Journal of Inorganic Chemistry*, *2008*(32), 5015–5019. https://doi.org/10.1002/ejic.200800876
2) Marks, W. R., Reinheimer, E. W., Seda, T., Zakharov, L. N., & Gilbertson, J. D. (2021b). NO Coupling by Nonclassical Dinuclear Dinitrosyliron Complexes to Form N_2O Dictated by Hemilability. *Inorg. Chem.* **60**, 15901–15909. https://doi.org/10.1021/acs.inorgchem.1c02285
3) Cordeiro, S.; Pereira, L.; Simoes, M. de O.; Marques, M. de F. Synthesis and Evaluation of New Bis(Imino) Pyridine Based Catalysts for Ethylene Polymerization. *Chemistry & Chemical Technology* **2016**, *10* (4), 413–421. https://doi.org/10.23939/chcht10.04.413.
4) Sigen A, et al. Direct C□H Arylation of Unactivated Arenes with Aryl Halides Promoted by Bis(Imino)Pyridine Derivatives. *Asian Journal of Organic Chemistry*, vol. 2, no. 10, 1 Oct. 2013, pp. 857–861, https://doi.org/10.1002/ajoc.201300129. Accessed 24 Dec. 2024.
5) Bruker, *SAINT*, *V8.40B*, Bruker AXS Inc., Madison, Wisconsin, USA.
6) Krause, L., Herbst-Irmer, R., Sheldrick, G. M., & Stalke, D. (2015). Comparison of silver and molybdenum microfocus X-ray sources for single-crystal structure determination. *Journal of Applied Crystallography*, *48*(1), 3–10. https://doi.org/10.1107/s1600576714022985
7) Sheldrick, G. M. (2015). SHELXT– Integrated space-group and crystal-structure determination. *Acta Crystallographica Section a Foundations and Advances*, *71*(1), 3–8. https://doi.org/10.1107/s2053273314026370
8) Sheldrick, George M. “Crystal Structure Refinement WithSHELXL.” *Acta Crystallographica Section c Structural Chemistry*, vol. 71, no. 1, 1 Jan. 2015, pp. 3–8, scripts.iucr.org/cgi-bin/paper?fa3356, https://doi.org/10.1107/s2053229614024218.
9) Bruker (2021). APEX4. Bruker AXS Inc., Madison, Wisconsin, USA.
10) Dolomanov, Oleg V., et al. “OLEX2: A Complete Structure Solution, Refinement and Analysis Program.” *Journal of Applied Crystallography*, vol. 42, no. 2, 24 Jan. 2009, pp. 339–341, https://doi.org/10.1107/s0021889808042726.
11) Sheldrick, G. M. SADABS; Area Detector Absorption Correction; University of Göttingen; Göttingen, Germany, **2001**.
12) Sheldrick, George M. “A Short History of SHELX.” *Acta Crystallographica. Section A, Foundations of Crystallography*, vol. 64, no. Pt 1, 2008, pp. 112–122, www.ncbi.nlm.nih.gov/pubmed/18156677, https://doi.org/10.1107/S0108767307043930.
13) Becke, A. D. “Density-Functional Exchange-Energy Approximation with Correct Asymptotic Behavior.” *Physical Review A*, vol. 38, no. 6, 1 Sept. 1988, pp. 3098–3100, https://doi.org/10.1103/physreva.38.3098.
14) Lee, Chengteh, et al. “Development of the Colle-Salvetti Correlation-Energy Formula into a Functional of the Electron Density.” *Physical Review B*, vol. 37, no. 2, 15 Jan. 1988, pp. 785–789, https://doi.org/10.1103/physrevb.37.785.
15) Weigend, Florian, and Reinhart Ahlrichs. “Balanced Basis Sets of Split Valence, Triple Zeta Valence and Quadruple Zeta Valence Quality for H to Rn: Design and Assessment of Accuracy.” *Physical Chemistry Chemical Physics*, vol. 7, no. 18, 2005, pp. 3297–3305, https://doi.org/10.1039/b508541a.
16) Grimme, Stefan, et al. “A Consistent and Accurate Ab Initio Parametrization of Density Functional Dispersion Correction (DFT-D) for the 94 Elements H-Pu.” *The Journal of Chemical Physics*, vol. 132, no. 15, 21 Apr. 2010, p. 154104, https://doi.org/10.1063/1.3382344.
17) Neese, Frank. “Software Update: The ORCA Program System, Version 4.0.” *WIREs Computational Molecular Science*, vol. 8, no. 1, 17 July 2017, https://doi.org/10.1002/wcms.1327.
